# The Role of Inflammatory Biomarkers in Mediating the Effect of Inflammatory Bowel Disease on nonmalignant Digestive System Diseases: A Multivariable Mendelian Randomized Study

**DOI:** 10.1155/2024/1266139

**Published:** 2024-03-18

**Authors:** Shu Zhou, Qi Sun, Ning Gao, Zekai Hu, Junjun Jia, Jiangwei Song, Guocong Xu, Aiqiang Dong, Weiliang Xia, Jiafeng Wu

**Affiliations:** ^1^Hangzhou Ninth People's Hospital, Hangzhou, China; ^2^Division of Hepatobiliary and Pancreatic Surgery, Department of Surgery, First Affiliated Hospital, School of Medicine, Zhejiang University, Qingchun Road 79, Hangzhou 310003, China; ^3^Key Laboratory of Combined Multi-Organ Transplantation, Ministry of Public Health, First Affiliated Hospital, School of Medicine, Zhejiang University, Qingchun Road 79, Hangzhou 310003, China; ^4^Department of Cardiovascular Surgery, The Second Affiliated Hospital of Zhejiang University School of Medicine, Hangzhou, China

## Abstract

**Background:**

While observation studies have shown a positive correlation between inflammatory bowel disease (IBD) and the risk of nonmalignant digestive system diseases, a definitive causal relationship has not yet been clearly established.

**Methods:**

Mendelian randomization (MR) was employed to investigate the potential causal association between genetic susceptibility to IBD and nonmalignant gastrointestinal diseases. Genetic variants were extracted as instrumental variables (IVs) from a genome-wide association study (GWAS) meta-analysis, which included 12,194 cases of Crohn's disease (CD) and 28,072 control cases of European ancestry. The GWAS for ulcerative colitis (UC) included 12,366 UC and 33,609 control cases of European ancestry. All IVs reached genome-wide significance (GWAS *p* value <5 × 10^−8^). Summary-level data for acute pancreatitis (AP), irritable bowel syndrome (IBS), gastroesophageal reflux disease, cholelithiasis, and CeD (celiac disease) were obtained from the GWAS meta-analysis and the FinnGen dataset. Summary-level data on relevant inflammatory factors were provided by the International Genetic Consortium. Univariate MR analysis was conducted using inverse variance weighting as the primary method for estimating causal effects. Multivariate MR analyses were also performed to detect possible mediators.

**Results:**

Genetic susceptibility to UC was associated with an increased risk of AP (OR = 1.08; 95% CI = 1.03–1.13; *p*=0.002) and IBS odds ratio (OR] = 1.07; 95% confidence interval (CI] = 1.03–1.11; (*p* < 0.001). In terms of potential mediators, interleukin 6 (IL-6) had a driving effect on the association between UC and AP. There was no apparent evidence of increased risk with CD. Meanwhile, genetic susceptibility to CD increases the risk of CeD (OR = 1.14; 95% CI = 1.03–1.25; *p*=0.01).

**Conclusions:**

The evidence suggests that UC is associated with an elevated risk of AP and IBS, and IL-6 may be responsible in AP. CD is associated with an increased risk of developing CeD. Implementing a proactive monitoring program for assessing the risk of gastrointestinal diseases in UC patients, particularly those with elevated IL-6 levels, may be of interest. In addition, the presence of AP and IBS may indicate the presence of UC. Preventing CeD is an essential consideration in the therapeutic management of patients with CD.

## 1. Background

Nonmalignant digestive system diseases (NMDSD) encompass a broad category of noncancerous gastrointestinal, hepatobiliary, and pancreatic disorders, including conditions such as irritable bowel syndrome (IBS), gastroesophageal reflux disease (GERD), acute pancreatitis (AP), celiac disease (CeD), and cholelithiasis [1]. NMDSD imposes a substantial medical and economic burden, and given the severe clinical and societal consequences they entail, it is imperative to elucidate their associated risk factors to facilitate timely treatment and prevention [1].

Numerous epidemiological studies have confirmed a strong correlation between inflammatory bowel disease (IBD) and NMDSD [2–5]. IBD represents a chronic and recurrent disorder of the gastrointestinal tract, with its two primary forms being ulcerative colitis (UC) and Crohn's disease (CD) [6]. Approximately 0.46% of adults in America have had IBD [7], and it affects approximately 6.8 million individuals worldwide [8]. Beyond gastrointestinal symptoms, many IBD patients frequently experience extraintestinal manifestations.

Several investigations have indicated that individuals with IBD are more susceptible to NMDSD [9], likely owing to the sustained inflammatory state and immune activation associated with IBD [10–12]. A meta-analysis, for instance, found a significantly higher prevalence of IBS in patients with IBD compared to controls [13]. Meanwhile, a meta-analysis showed that patients with IBD had a 3.96-fold increased risk of CED compared to the general population [14]. Furthermore, IBD has been linked to an increased risk of cholelithiasis in a cohort study [15]. Moreover, a multicenter study revealed that individuals with IBD were more prone to developing AP [16]. However, it is worth noting that many agents used to treat IBD, such as salicylates, azathioprine, 6-mercaptopurine, and glucocorticosteroids, can also induce NMDSD. The evidence pertaining to the relationship between IBD and the risk of NMDSD is still contradictory and limited. Thus, it is crucial to establish IBD and NMDSD as having a causal relationship.

Mendelian randomization, an emerging technique in medical research, has been used to assess causal relationships between specific exposures and outcomes by utilizing genetic variations as instrumental variables (IVs) [17]. MR is highly effective in mitigating common confounding and reverse causation biases encountered in traditional observational studies [18]. As genetic variants are randomly inherited by offspring during meiosis and conception, MR operates on principles akin to randomized clinical trials (RCTs), which are often considered the gold standard for establishing causal relationships. Consequently, MR has emerged as a pivotal epidemiological method for inferring causality when RCTs are not feasible. In this context, our study aimed to achieve two primary objectives: (1) in this context, our study aimed to achieve two primary objectives; (2) explore the potential links between IBD and clinical inflammatory markers, including interleukin-6 (Il-6), C-reactive protein (CRP), and tumor necrosis factor-*α* (TNF-*α*), employing univariate MR (UVMR). In addition, utilizing multivariate MR (MVMR), we sought to examine whether these inflammatory markers acted as potential mediators in the association between IBD and NMDSD.

## 2. Methods

### 2.1. Study Design

**Figure 1:**
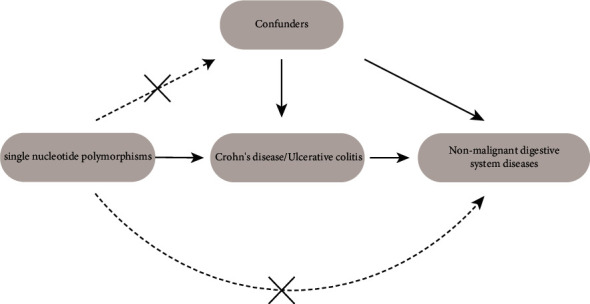
Main design of this study.

In order to examine the potential causal link between IBD and prevalent NMDSD, including AP, IBS, GERD, and cholelithiasis, a UVMR analysis was conducted. The MR study followed a comprehensive procedure, as illustrated in Figure 1, guided by three fundamental assumptions: a. IVs are closely related to IBD; b. IVs must be independent of potential confounding factors; c. IVs should not be relevant to the NMDSD unless by way of IBD [19].

Our study proceeded in several steps. Initially, we examined the link between genetic susceptibility to IBD and NMDSD using UVMR analysis. To delve into potential mechanisms, we explored the association between IBD and clinically common inflammatory biomarkers. Subsequently, we conducted MVMR to investigate the mediating role of inflammatory factors in the relationship between genetic susceptibility to IBD and NMDSD. It is important to note that our study exclusively utilized publicly available summary-level data from genome-wide association studies (GWAS), obviating the need for additional ethical permissions.

We performed the analysis of this study according to MR guidelines [20]. All analyses were conducted with R software (4.1.2), using the “TwoSampleMR,” “MRPRESSO,” and “Mr. Rap” packages.

### 2.2. Data Source

We obtained summary data for each phenotype from various sources, as detailed in Table 1. Summary statistics for IBD were acquired from a GWAS meta-analysis involving 59,957 participants, primarily of European ancestry. This dataset included 12,194 CD and 12,366 UC patients [21]. All cases in this study were diagnosed through recognized radiological, endoscopic, and histopathological evaluations that met the clinical diagnostic criteria for IBD.

Summary-level statistics data for AP (FinnGen data field: K11_ACUTPANC), cholelithiasis (FinnGen data field: K11_CHOLELITH), and IBS (FinnGen data field: K11_IBS) were obtained from the FinnGen Consortium [22], and all participants were of European ancestry (AP cases/controls: 3,022/195,144; cholelithiasis: 19,023/195,144; IBS: 4,605/182,423). GERD (cases/controls: 129,080/473,524) and Ced (cases/controls: 11,812/11,837) were derived from publicly available summary-level GWAS [23, 24].

Summary-level statistics for IL-6 were obtained from the Systematic and Combined AnaLysis of Olink Proteins (SCALLOP) consortium, which included 21,758 participants of European ancestry [25]. The GWAS meta-analysis of CRP included 204,402 European individuals [26]. TNF-*α* was derived from a GWAS of 3,454 Finns [27].

### 2.3. Selection

We only included single nucleotide polymorphisms (SNPs) that demonstrated genome-wide significance (*p* value <5 × 10^−8^). SNPs with lower minor allele frequencies (MAF < 0.01) were removed. Further, linkage disequilibrium (LD, *r*^2^ > 0.01) among the selected SNPs was assessed to screen out independent SNPs as IVs. We harmonized summary statistics of exposures and outcomes and excluded palindromic SNPs. The beta and standard error (SE) coefficients of these SNPs were scaled by the log-transformed odds of IBD. We performed strength assessments for the screened SNPs to avoid weak instrumental bias. If *F* > 10, it indicated that the association between the IVs and IBD was sufficiently robust, and the likelihood of weak instrumental bias affecting the results' reliability was minimized [28].

### 2.4. Statistical Analysis

For UVMR analysis, the inverse variance weighting (IVW) method was used as the primary analysis method. The choice of analytical model depended on the presence or absence of heterogeneity in the sensitivity analysis. If heterogeneity existed, the random-effects IVW method was selected, and if there was no heterogeneity, the fixed-effects IVW method was opted [29].

To bolster our causal inference, we also conducted supplementary analyses using various MR models, including the MR-robust adjusted profile score (RAPS) [30], weighted median analysis [31], maximum-likelihood estimation [32], MR-Egger method [33], and MR-pleiotropy residual sum and outlier (MR-PRESSO) [34]. Although IVW provides the most accurate estimates, horizontal polymorphism and heterogeneity may affect its accuracy. MR-RAPS is an extension of the IVW method that can increase statistical validity and effectively address the issue of horizontal pleiotropy by including numerous weak IVs [30]. In the weighted median analysis, over half of the weights were derived from effective SNPs [31]. The MR-Egger method detects horizontal pleiotropy by means of intercept tests [33]. However, for most cases, the statistical power of this analysis is weak. The MR-PRESSO method reduces the effect of horizontal pleiotropy by identifying and removing outliers [34]. Each of these analytical approaches was selected based on its unique assumptions and ability to produce unbiased causal estimates. You can find more details on each method in Additional file 1: Table S1. When examining the association between IBD and NMDSD, a causal relationship was considered significant at a threshold of *p* < 0.0125 (Bonferroni-corrected, 0.05 for 4 outcomes).

Given that horizontal pleiotropy can interfere with MR estimation to some extent, a sensitivity analysis was performed (Table 2). We evaluated heterogeneity using the Cochran's Q-test. Potential heterogeneity was present when *p* < 0.05. The MR-Egger intercept was utilized to detect horizontal pleiotropy. Excessive horizontal pleiotropy indicates that this analysis violates the fundamental assumptions of MR analysis, making the results less reliable. To ensure that the results were not driven by strong correlations of individual SNPs, we performed a leave-one-out analysis (Additional file 2: Figures S1–S3).

In consideration of the potential associations between inflammatory factors and NMDSD, we conducted MVMR to explore whether the observed associations were influenced by clinically common inflammatory markers [35]. This approach allows us to dissect the total effect of IBD on NMDSD, estimated through our UVMR, into two components: the direct effect and the indirect effect mediated by the inflammatory markers. To calculate the proportion of the effect mediated by inflammatory factors, we divided the direct effect by the total effect and subtracted it from 1. Notably, this method assumes that the medium is a continuous variable to eliminate any bias in the estimation of mediating effects [36].

## 3. Results

There was an association between susceptibility to UC and an increased risk of IBS and AP. Furthermore, interleukin-6 (IL-6) played a mediating role in these associations. Furthermore, genetic susceptibility to CD disease is associated with a higher risk of CeD. The F-statistic for all IVs was greater than 10, suggesting that the reliability of the results was unlikely to have been affected by weak instrumental bias (additional file 1: Table S2). The summary data for all SNPs used for statistical analysis are shown in additional file 1 Tables S3–S13.

### 3.1. UVMR Analysis

**Figure 2:**
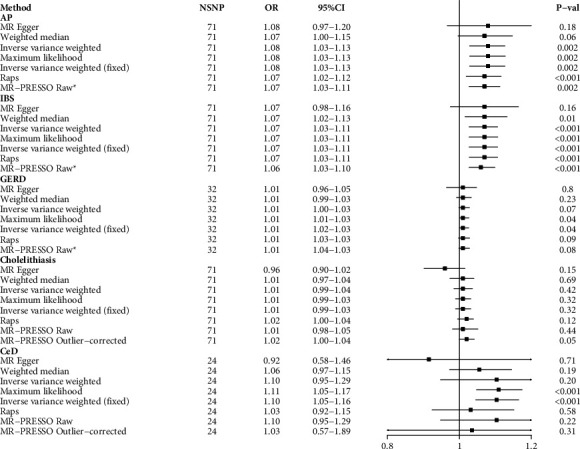
The causal association between ulcerative colitis and nonmalignant digestive system diseases; AP, acute pancreatitis; IBS, irritable bowel syndrome; GERD, gastroesophageal reflux disease; SNP, single nucleotide polymorphisms; OR, odds ratio; raps, robustly adjusted profile score; CeD, celiac disease; ^*∗*^no outlier was detected.

As shown in Figure 2, genetic susceptibility to UC was strongly associated with a high risk of IBS (odds ratio [OR] = 1.07; 95% confidence interval [CI] = 1.03–1.11; *p* < 0.002) and AP (OR = 1.08; 95% CI = 1.03–1.13; *p*=0.002), but not cholelithiasis (OR = 1.01; 95% CI = 0.97–1.04; *p*=0.42),CeD (OR = 1.10; 95% CI = 0.95–1.29; *p*=0.20), or GERD (OR = 1.01; 95% CI = 1.02–1.03; *p*=0.04).

The causal estimates obtained from the different MR statistical models were generally consistent, except for the MR-Egger which produced causal estimates with lower precision. As described previously, in most cases, the MR-Egger method does not provide sufficient statistical power [33]. No outliers were found by MR-PRESSO analysis of IBS and AP, further indicating the robustness of the results. Cochran's Q-test revealed no heterogeneity in the analysis of the association of UC with AP, IBS, and GERD; thus, the fixed-effects IVW method was used as the “gold standard” for this analysis. Heterogeneity was detected in the association of UC with cholelithiasis; thus, the random-effects model is considered as the gold standard (Figure 2). MR-Egger intercepts revealed weak horizontal pleiotropy. In addition, the leave-one-out method suggested that the results of causal estimation were not mediated by a single SNP (Additional file 2: Figures S1–S3). Forest plots and funnel plots were used to visualize the heterogeneity analysis (Additional file 2: Figures S1–S6).

**Figure 3:**
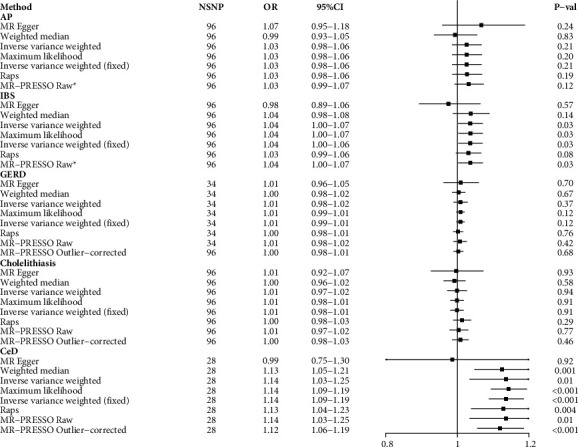
The causal association between Crohn's disease and nonmalignant digestive system diseases; AP, acute pancreatitis; IBS, irritable bowel syndrome; GERD, gastroesophageal reflux disease; SNP, single nucleotide polymorphisms; OR, odds ratio; raps, robustly adjusted profile score; CeD, celiac disease; ^*∗*^no outlier was detected.

In addition, genetic susceptibility to CD was associated with an elevated risk of CeD (OR = 1.14; 95% CI = 1.03–1.25; *p*=0.01), but not with other NMDSD (Figure 3).

**Figure 4:**
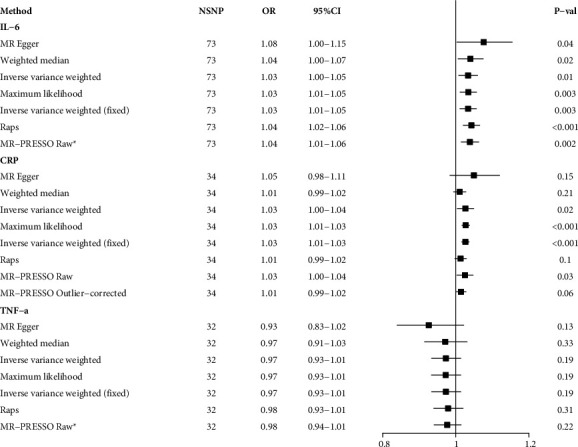
The causal association between ulcerative colitis and inflammatory factors IL-6, interleukin 6; CRP, C-reactive protein; TNF-*α*, tumor necrosis factor-*α*; SNP, single nucleotide polymorphisms.

**Figure 5:**
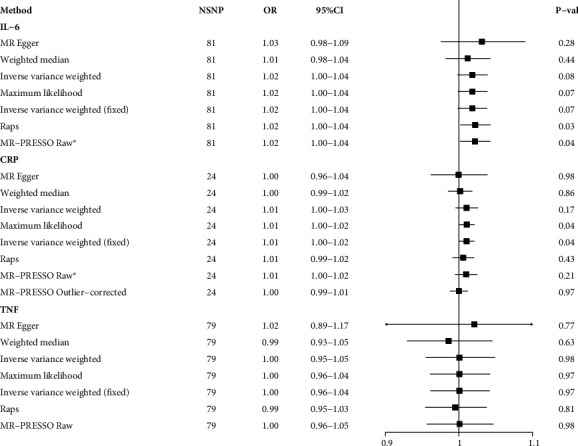
The causal association between Crohn's disease and inflammatory factors; IL-6, interleukin 6; CRP, C-reactive protein; TNF-*α*, tumor necrosis factor-*α*; SNP, single nucleotide polymorphisms.

In terms of the inflammatory markers, genetic susceptibility to UC was associated with elevated levels of IL-6 and CRP (Figure 4). The MR-PRESSO method detected the presence of outliers in the analysis of CRP, and the correlation was altered after removal of these outliers. Therefore, to ensure the reliability of the results, CRP was not included in the subsequent mediation analysis. We detected heterogeneity in the analysis of IL-6 but no horizontal pleiotropy (Table 2). After removal of the outliers identified by the MR-PRESSO method, the correlations remained consistent. Moreover, multiple MR analysis models yielded consistent analytical results, indicating that this result was sufficiently reliable. In addition, we did not find a correlation between genetic susceptibility to UC and TNF-*α* (Figure 4). There was no evidence that genetic susceptibility to CD associated with IL-6, CRP, and TNF levels (Figure 5).

### 3.2. MVMR Analysis

The negative effect of genetic susceptibility to UC on AP (OR = 1.07; 95% CI = 1.02–1.11; *p*=0.002) which was observed in the UVMR analysis was diminished after adjusting for genetically predicted IL-6 levels using MVMR analysis. Furthermore, the association between UC and IBS (OR = 1.07; 95% CI = 1.02–1.10; *p* < 0.001) was only slightly changed in the MVMR analysis **(**Table 3**)**.

## 4. Discussion

Our results show CD may increase the risk of CeD. Furthermore, this study is the first to examine the causal relationship between IBD and specific types of NMDSD. Our study revealed that UC is associated with a higher risk of AP and IBS. In addition, genetic susceptibility to UC was associated with elevated IL-6 levels. Further MVMR analysis showed that IL-6 had an important driving effect on the association between UC and AP. These results shed light on the potential mechanisms underlying the increased risk of AP in individuals with UC and the role of IL-6 in this relationship.

Recent years have seen a growing volume of observational studies linking IBD to the risk of NMDSD [37]. For example, a Swedish cohort study showed that people with CD were at a 4.36% higher risk of CeD than normal people. [38]. Moreover, one observational study found patients with pancreatitis had a 10-fold increased risk of IBD compared with controls [39]. A meta-analysis showed that the pooled prevalence of IBS in patients with IBD was significantly higher than in the general population [13]. However, these studies could not clearly establish causality because the relationships remained largely associative rather than causal.

Our results on UC can be attributed to several potential mechanisms. Firstly, patients afflicted by UC often exhibit visceral hypersensitivity, a phenomenon that persists even during periods of remission [40–42]. This heightened visceral sensitivity is recognized as a fundamental pathogenic mechanism underlying IBS [43]. Notably, this phenomenon may be linked to an increased release of proinflammatory cytokines [44]. Furthermore, individuals with UC tend to have elevated levels of circulating markers, such as IL-6 [45]. According to a study conducted on guinea pigs, it functions as a neuromodulator, potentially exacerbating bowel symptoms in cases of IBS. Moreover, inhibition of the IL-6 receptors can ameliorate IBS-like symptoms by decreasing the expression of the T-type calcium channel Ca_v_ 3.2, which plays a central role in visceral pain [46]. In addition, the diminished quality of life often experienced by individuals with UC could conceivably contribute to the development of IBS [47, 48].

Recent evidence has shown that there is a robust link between AP and IL-6, which exhibits high expression in UC [49, 50]. AP is a sudden inflammation of the pancreatic tissue, caused by the activation of several inflammatory mediators [51]. Earlier reports have substantiated that human IL-6 exacerbates cerulein-induced AP in murine models. Furthermore, the signal transducer and activator of transcription-3 (STAT-3) assume a pivotal role in triggering the inflammatory response [52]. The application of anti-IL-6 antibodies has shown promise in mitigating severe AP by inhibiting IL-6 and quelling the activation of STAT-3 [51, 53]. In addition, previous data suggests that IL-6 trans-signaling may play an essential role in AP-related acute lung injury in mouse models [54]. In a cross-sectional investigation, pancreatic duct abnormalities were found in approximately 16% of cases with UC [55]. These abnormalities might contribute to the pathogenesis and pain of AP [56]. Previous studies have shown antibodies to Saccharomyces cerevisiae (ASCA) manifest at elevated titers in patients with CD but not in patients with UC or general population [57]. Elevated ASCA titers may respond to increased intestinal permeability, and the occurrence of CeD is strongly associated with increased intestinal mucosal permeability defects. Besides, diet cannot be ignored in the development of CeD. In a cohort study, a high-inflammatory-potential dietary pattern was shown to be associated with CD but not with UC [58]. This high-inflammatory potential diet in CD patients may increase the risk of CeD.

This study substantiates the proposition that inflammation plays a pivotal role in driving the susceptibility to IB) and AP in individuals with UC, with IL-6 emerging as a mediating factor in these connections. Our findings advocate for vigilant monitoring of UC in patients diagnosed with AP, particularly those exhibiting heightened IL-6 levels. Such an approach could facilitate the early detection and diagnosis of UC. Furthermore, our results signify the importance of proactively conducting endoscopic examinations in individuals diagnosed with IBS. This strategy is paramount in the realm of precision medicine, given the considerable divergence in treatment approaches for UC and IBS in clinical practice [59, 60]. Although UC remains without a cure, early intervention holds the potential to decelerate disease progression and alleviate symptoms [61]. By doing so, we can alleviate the healthcare burden on society and empower patients through timely detection and intervention.

This MR study evaluating the association between genetic susceptibility to IBD and NMDSD boasts several significant strengths. Firstly, MR analysis's inherent characteristics have effectively mitigated the influence of confounding variables and reverse causality on the study outcomes. Meanwhile, multiple UVMR analysis models and sensitivity analyses were used to assess whether this study violated MR assumptions. Secondly, the summary-level statistics obtained from large-scale GWASs have substantially bolstered their statistical robustness. Furthermore, the acquisition of data pertaining to exposures, mediators, and outcomes from nonoverlapping GWASs has further minimized potential biases [62]. Finally, the application of mediated MR analysis has notably reduced the impact of unobservable confounding factors, a common challenge in traditional mediated analyses [63]. Through the use of MVMR analysis, this study effectively unraveled the causal influence of specific inflammatory factors on the connection between genetic susceptibility to UC and NMDSD.

Nonetheless, it is essential to acknowledge the limitations of this study. First, the potential biological role of the SNPs included in this study remains incompletely elucidated. Therefore, horizontal pleiotropy cannot be completely eliminated. Fortunately, the MVMR models based on different assumptions yielded consistent conclusions, and sensitivity analyses did not detect horizontal pleiotropy. Second, this MR analysis was performed based on data from participants of European ancestry. Consequently, the generalizability of the results to other ethnic populations might be limited. Third, due to sample size limitations, we examined only three common inflammatory factors.

## 5. Conclusions

We found that people with CD are more likely to suffer from CeD. Furthermore, our research highlights a substantial connection between genetic susceptibility to UC and an elevated risk of AP and IBS. IL-6 appears to be a key driver of the association between UC and AP. These results underscore the importance of proactive monitoring of UC patients for gastrointestinal diseases, particularly in cases where IL-6 levels are elevated. Additionally, mitigating inflammation could serve as an effective preventive measure against gastrointestinal disorders in individuals with UC. The presence of these disorders may also serve as an indicator of underlying UC.

## Figures and Tables

**Table 1:** Overview of the studies and consortia.

Traits	Data sources	Sample size (case/control)	Ancestry	Adjustments in the GWAS
UC	de Lange et al.	12,366/33,609	European	The first ten genetic principal components
CD	de Lange et al.	12,194/28,072	European	The first ten genetic principal components
AP	FinnGen	3,022/195,144	European	Age, sex, and the first ten genetic principal components
IBS	FinnGen	4,605/182,423	European	Age, sex, and the first ten genetic principal components
GERD	Ong et al.	129,080/473,524	European	Age, genetic sex, the first ten principal components, and cryptic relatedness
Cholelithiasis	FinnGen	19,023/195,144	European	Age, sex, and the first ten genetic principal components
CeD	Trynka et al.	11,812/11,837	European	Age, sex, and the first ten genetic principal components
IL-6	SCALLOP	21,758	European	Age and sex
CRP	Ligthart et al.	204,402	European	Age, sex, and population substructure
TNF-*α*	Cardiovascular risk in young finns study and the FINRISK studies	8293	European	Age, sex, body mass index, and the first ten genetic principal components

UC, ulcerative colitis; CD, Crohn's disease, AP, acute pancreatitis; IBS, irritable bowel syndrome; GERD, gastroesophageal reflux disease; CeD, celiac disease; IL-6, interleukin 6; CRP, C-reactive protein; TNF-*α*, tumor necrosis factor-*α*; SCALLOP, systematic and combined analysis of olink proteins.

**Table 2:** Pleiotropy and heterogeneity test of the UC/CD IVs from nonmalignant digestive system diseases GWAS.

		Pleiotropy test			Heterogeneity test			
		MR-egger			MR-egger		IVW	
		Intercept	SE	Pval	Q	Q_Pval	Q	Q_Pval
UC	AP	−6.28*E* − 05	0.009	0.99	58.04	0.82	58.04	0.85
	IBS	9.82*E* − 05	0.008	0.99	60.97	0.74	60.97	0.77
	GERD	0.001	0.003	0.70	39.30	0.12	39.50	0.14
	Cholelithiasis	0.010	0.005	0.05	99.76	0.009	105.40	0.004
	CeD	0.032	0.038	0.41	177.60	2.57*E* − 26	183.27	6.06*E* − 27
	IL-6	−0.007	0.006	0.23	92.81	0.04	94.71	0.04
	CRP	−0.004	0.005	0.47	162.55	2.10*E* − 19	165.24	1.61*E* − 19
	TNF-*α*	0.009	0.008	0.28	64.56	0.63	65.72	0.62

CD	AP	−0.007	0.009	0.44	95.59	0.43	96.21	0.45
	IBS	0.011	0.007	0.15	95.45	0.44	97.63	0.41
	GERD	−1.70*E* − 04	0.004	0.97	103.61	1.76*E* − 09	103.61	3.22*E* − 09
	Cholelithiasis	3.60*E* − 04	0.006	0.95	215.61	1.51*E* − 11	215.62	2.30*E* − 11
	CeD	0.026	0.025	0.29	125.16	6.16*E* − 15	130.66	1.49*E* − 15
	IL-6	−0.002	0.004	0.63	84.37	0.32	84.61	0.34
	CRP	0.004	0.003	0.55	50.76	0.0005	51.62	0.0006
	TNF-*α*	−0.003	0.011	0.76	105.64	0.017	105.77	0.02

IVW, inverse variance weighting; UC, ulcerative colitis; AP, acute pancreatitis; IBS, irritable bowel syndrome; GERD, gastroesophageal reflux disease; IL-6, interleukin 6; CRP, C-reactive protein; TNF-*α*, tumor necrosis factor-*α*; CD, Crohn's disease; GWAS, genome-wide significant; CeD, celiac disease.

**Table 3:** MVMR analysis of the effect of ulcerative colitis on acute pancreatitis and irritable bowel syndrome.

Outcome	OR	95% CI	*p* value	Mediation effect (%)
Acute pancreatitis				
IVW, crude	1.08	1.03–1.13	0.002	
Adjusted for IL-6	1.07	1.02–1.11	0.002	9.44
Irritable bowel syndrome				
IVW, crude	1.07	1.03–1.11	<0.001	
Adjusted for IL-6	1.07	1.02–1.10	<0.001	0.55

OR, odds ratio; IVW, inverse variance weighting; IL-6, interleukin 6.

## Data Availability

The datasets used and/or analysed during the current study available from the corresponding author on reasonable request.

## Supplementary Materials

Supplementary Materials Additional file 1: Table S1. The differences between the various methods. Table S2. The F-statistic in instrumental variable strength analysis. Table S3. Summary information on the SNPs used as genetic instruments for the CD in MR study in acute pancreatitis. Table S4. Summary information on the SNPs used as genetic instruments for the CD in MR study in irritable bowel syndrome. Table S5. Summary information on the SNPs used as genetic instruments for the CD in MR study in gastroesophageal reflux disease. Table S6. Summary information on the SNPs used as genetic instruments for the CD in MR study in cholelithiasis. Table S7. Summary information on the SNPs used as genetic instruments for the CD in MR study in celiac disease. Table S8. Summary information on the SNPs used as genetic instruments for the UC in MR study in acute pancreatitis. Table S9. Summary information on the SNPs used as genetic instruments for the UC in MR study in irritable bowel syndrome. Table S10. Summary information on the SNPs used as genetic instruments for the UC in MR study in gastroesophageal reflux disease. Table S11. Summary information on the SNPs used as genetic instruments for the UC in MR study in Cholelithiasis. Table S12. Summary information on the SNPs used as genetic instruments for the UC in MR study in Interleukin-6. Table S13. Summary information on the SNPs used as genetic instruments for the UC in MR study in c-reactive protein. Table S14. Summary information on the SNPs used as genetic instruments for the UC in MR study in Tumor necrosis factor-*α*. Table S15. Summary information on the SNPs used as genetic instruments for the UC in MR study in celiac disease. Additional file 2: Figure S1 Leave-one-out analysis and forest plots for causal effect of Crohn's disease on outcome. Figure S2. Leave-one-out analysis and forest plots for causal effect of ulcerative colitis on outcome. Figure S3. Leave-one-out analysis and forest plots for causal effect of ulcerative colitis on interleukin-6, c-reactive protein and Tumor necrosis factor-*α*. Figure S4. Funnel plot for causal effect of Crohn's disease on outcome; Figure S5 Funnel plot for causal effect of ulcerative colitis on outcome. Figure S6. Funnel plot for causal effect of ulcerative colitis on interleukin-6, c-reactive protein, and Tumor necrosis factor-*α*.
