# Supplementary material for: The Role of Inflammatory Biomarkers in Mediating the Effect of Inflammatory Bowel Disease on nonmalignant Digestive System Diseases: A Multivariable Mendelian Randomized Study
Source: Can J Gastroenterol Hepatol. 2024 Mar 18;2024:1266139. doi: 10.1155/2024/1266139 (PMC10963109; doi:10.1155/2024/1266139)
Supplement: Supplementary Materials — Additional file 1: Table S1. The differences between the various methods. Table S2. The F-statistic in instrumental variable strength analysis. Table S3. Summary information on the SNPs used as genetic instruments for the CD in MR study in acute pancreatitis. Table S4. Summary information on the SNPs used as genetic instruments for the CD in MR study in irritable bowel syndrome. Table S5. Summary information on the SNPs used as genetic instruments for the CD in MR study in gastroesophageal reflux disease. Table S6. Summary information on the SNPs used as genetic instruments for the CD in MR study in cholelithiasis. Table S7. Summary information on the SNPs used as genetic instruments for the CD in MR study in celiac disease. Table S8. Summary information on the SNPs used as genetic instruments for the UC in MR study in acute pancreatitis. Table S9. Summary information on the SNPs used as genetic instruments for the UC in MR study in irritable bowel syndrome. Table S10. Summary information on the SNPs used as genetic instruments for the UC in MR study in gastroesophageal reflux disease. Table S11. Summary information on the SNPs used as genetic instruments for the UC in MR study in Cholelithiasis. Table S12. Summary information on the SNPs used as genetic instruments for the UC in MR study in Interleukin-6. Table S13. Summary information on the SNPs used as genetic instruments for the UC in MR study in c-reactive protein. Table S14. Summary information on the SNPs used as genetic instruments for the UC in MR study in Tumor necrosis factor-α. Table S15. Summary information on the SNPs used as genetic instruments for the UC in MR study in celiac disease. Additional file 2: Figure S1 Leave-one-out analysis and forest plots for causal effect of Crohn's disease on outcome. Figure S2. Leave-one-out analysis and forest plots for causal effect of ulcerative colitis on outcome. Figure S3. Leave-one-out analysis and forest plots for causal effect of ulcerative colitis on inter [file 1266139.f1.zip › Additional files1 (1).docx]

Table S1 The differences between the various methods

| methods | differences |
| --- | --- |
| Inverse variance weighting with multiplicative random effects | Inverse variance weighting regressed instrument-outcome associations on instrument-exposure associations for the instruments, weighted by the inverse of the variance of the instrument-outcome association, with the intercept constrained to 0. Inverse variance weighting gives valid causal estimate when all instruments are valid instruments, i.e., instruments related to exposures; instrument independent of confounders, and exclusion restriction assumption. Inverse variance weighting will produce valid causal estimate in the presence of balanced horizontal pleiotropy and the instrument strength independent of direct effect (InSIDE) assumption is satisfied. |
| MR-Egger | MR-Egger method is similar to inverse variance weighting, but the intercept is not constrained to 0. As such, the MR-intercept test evaluates whether there is presence of overall horizontal pleiotropy. This method gives valid causal estimate even when all instruments are invalid. However, it relies on InSIDE assumption, which could be violated if the instruments are related to the confounders of exposure-outcome association. |
| Weighted median method | The weighted median method takes the weighted median of the Wald ratio as the causal estimate, weighted by the instrument-exposure association. Weighted median method gives valid causal estimate when more than 50% of the information is derived from valid instruments. |
| MR-PRESSO | MR-PRESSO provides a statistical test of (a) the presence of unbalanced horizontal pleiotropy (Global test), (b) generates a causal effect estimate corrected for unbalanced horizontal pleiotropy by outlier removal (Outlier corrected estimate), and (c) provides a statistical test comparing the estimate before and after the outlier removal (Distortion test). |

Table S2 The F-statistic in instrumental variable strength analysis

| Traits | | R^2^  (%) | *F-* statistic  (total) |
| --- | --- | --- | --- |
| UC |  |  |  |
|  | AP | 0.037415238 | 25.12998626 |
|  | IBS | 0.037406601 | 25.12396004 |
|  | GERD | 0.017407079 | 25.43385543 |
|  | Cholelithiasis | 0.037415369 | 25.13007768 |
|  | CeD | 3.8 | 75.51714 |
| CD |  |  |  |
|  | AP | 0.062161921 | 27.73424508 |
|  | IBS | 0.062158283 | 27.73251451 |
|  | GERD | 0.03126881 | 38.1935454 |
|  | Cholelithiasis | 0.062166898 | 27.73661311 |
|  | CeD | 7.03 | 108.6361 |

UC, Ulcerative colitis；AP, Acute pancreatitis; IBS Irritable, bowel syndrome; GERD, Gastroesophageal reflux disease；CeD celiac disease;

Table S3 Summary information on the SNPs used as genetic instruments for the CD in MR study in acute pancreatitis

| SNP | chr | os | A1 | A2 | Exposure (CD) | | | Outcome (AP) | | |
| --- | --- | --- | --- | --- | --- | --- | --- | --- | --- | --- |
|  |  |  |  |  | β | Se | *P* | β | Se | *P* |
| rs10052709 | 5 | 158760477 | G | C | -0.141 | 0.0242 | 5.76E-09 | 0.0498 | 0.0482 | 0.3022 |
| rs10055349 | 5 | 40441718 | A | G | 0.1734 | 0.019 | 5.59E-20 | -0.0164 | 0.0316 | 0.6041 |
| rs10114470 | 9 | 117547772 | C | T | 0.1687 | 0.0177 | 1.76E-21 | 0.039 | 0.0316 | 0.2175 |
| rs1012636 | 6 | 20674811 | T | G | 0.1291 | 0.0198 | 7.01E-11 | 0.0259 | 0.0299 | 0.3868 |
| rs10822050 | 10 | 64438771 | C | T | 0.1827 | 0.0162 | 2.35E-29 | -0.0111 | 0.0271 | 0.6834 |
| rs10884966 | 10 | 112185596 | A | G | 0.1131 | 0.0171 | 4.13E-11 | -0.0156 | 0.0276 | 0.5727 |
| rs11236797 | 11 | 76299649 | A | C | 0.176 | 0.0161 | 8.51E-28 | 0.0526 | 0.0269 | 0.050841 |
| rs114802258 | 1 | 160831855 | T | C | -0.2245 | 0.0384 | 5.11E-09 | -0.0483 | 0.056 | 0.3887 |
| rs1148246 | 10 | 35496626 | T | C | -0.1323 | 0.0167 | 2.09E-15 | 0.0613 | 0.0274 | 0.02518 |
| rs1157509 | 5 | 158786110 | G | A | 0.1519 | 0.0224 | 1.26E-11 | -0.0042 | 0.0368 | 0.9098 |
| rs11677002 | 2 | 28614401 | C | T | -0.1124 | 0.0163 | 4.57E-12 | 0.0399 | 0.0267 | 0.1348 |
| rs11683692 | 2 | 145509615 | C | T | -0.2144 | 0.038 | 1.75E-08 | -0.0072 | 0.0516 | 0.889 |
| rs11965964 | 6 | 33020604 | T | C | 0.3044 | 0.0529 | 8.83E-09 | 0.1037 | 0.1654 | 0.5305 |
| rs1250573 | 10 | 81042475 | A | G | -0.1522 | 0.0179 | 1.92E-17 | -0.0153 | 0.0275 | 0.578901 |
| rs1260326 | 2 | 27730940 | C | T | -0.1053 | 0.0161 | 6.32E-11 | 0.0388 | 0.0278 | 0.1627 |
| rs12936409 | 17 | 38043649 | T | C | 0.1426 | 0.016 | 4.31E-19 | 0.0073 | 0.0265 | 0.7831 |
| rs1297264 | 21 | 16816017 | G | A | -0.1769 | 0.0163 | 1.59E-27 | 0.0223 | 0.0271 | 0.4104 |
| rs13107325 | 4 | 103188709 | T | C | 0.2006 | 0.0284 | 1.66E-12 | 0.0928 | 0.1128 | 0.4106 |
| rs1321859 | 6 | 91011673 | T | C | -0.1049 | 0.0172 | 1.18E-09 | 0.0247 | 0.0312 | 0.4292 |
| rs1373904 | 13 | 44475398 | G | A | 0.141 | 0.0189 | 9.11E-14 | 0.0335 | 0.033 | 0.3098 |
| rs142770866 | 19 | 10525372 | A | G | 0.1753 | 0.0292 | 1.99E-09 | -0.0153 | 0.0489 | 0.753999 |
| rs144309607 | 19 | 10492274 | T | C | -0.3712 | 0.047 | 2.69E-15 | -0.0126 | 0.0771 | 0.8707 |
| rs145126485 | 16 | 50918662 | C | A | 0.5236 | 0.0431 | 6.40E-34 | -0.032 | 0.0842 | 0.703399 |
| rs145568234 | 6 | 32247045 | G | T | 0.8602 | 0.0633 | 4.31E-42 | 0.3604 | 0.2456 | 0.1422 |
| rs151175749 | 2 | 173424407 | G | C | 0.2483 | 0.0428 | 6.72E-09 | 0.0032 | 0.079 | 0.9675 |
| rs1583792 | 2 | 198900288 | T | C | -0.0882 | 0.016 | 3.26E-08 | 0.0004 | 0.0269 | 0.9879 |
| rs1633043 | 6 | 29732394 | G | A | -0.1266 | 0.0219 | 7.11E-09 | 0.0016 | 0.033 | 0.9611 |
| rs181826 | 5 | 141526057 | A | C | 0.1162 | 0.0167 | 3.24E-12 | 0.0337 | 0.0279 | 0.2263 |
| rs1870148 | 10 | 82271341 | A | G | 0.1351 | 0.0206 | 5.44E-11 | -0.0202 | 0.0314 | 0.5199 |
| rs1887428 | 9 | 4984530 | C | G | -0.166 | 0.0169 | 8.54E-23 | 0.0019 | 0.0273 | 0.9435 |
| rs194746 | 14 | 69282887 | T | C | 0.0975 | 0.0161 | 1.24E-09 | -0.0281 | 0.0265 | 0.2893 |
| rs1990684 | 16 | 50427554 | T | C | -0.1556 | 0.028 | 2.73E-08 | 0.0509 | 0.0369 | 0.1682 |
| rs2002695 | 10 | 30805480 | G | A | -0.1293 | 0.0189 | 8.31E-12 | 0.0347 | 0.029 | 0.2313 |
| rs2021511 | 16 | 11344903 | T | C | -0.1082 | 0.0182 | 2.63E-09 | -0.0755 | 0.03 | 0.0117 |
| rs2076756 | 16 | 50756881 | G | A | 0.385 | 0.0174 | 1.80E-108 | -0.0205 | 0.0368 | 0.5783 |
| rs2110735 | 2 | 103050925 | G | A | -0.1372 | 0.0185 | 1.20E-13 | 0.0052 | 0.0337 | 0.8781 |
| rs212408 | 6 | 159470242 | T | G | -0.1136 | 0.0167 | 9.12E-12 | -0.0119 | 0.029 | 0.681 |
| rs2143178 | 22 | 39660829 | C | T | -0.2087 | 0.0223 | 6.84E-21 | -0.0141 | 0.0414 | 0.733701 |
| rs2188962 | 5 | 131770805 | T | C | 0.2004 | 0.016 | 5.59E-36 | -0.0184 | 0.0285 | 0.5175 |
| rs2284553 | 21 | 34776695 | G | A | 0.1277 | 0.0165 | 1.14E-14 | 0.0208 | 0.0275 | 0.4496 |
| rs2476601 | 1 | 114377568 | G | A | 0.2312 | 0.0286 | 6.44E-16 | 0.046 | 0.0372 | 0.2171 |
| rs2523989 | 6 | 30078275 | T | C | -0.1693 | 0.0254 | 2.46E-11 | 0.0058 | 0.0379 | 0.878 |
| rs2581828 | 3 | 53133149 | G | C | -0.0941 | 0.0162 | 6.46E-09 | 0.0207 | 0.0273 | 0.4492 |
| rs2675670 | 10 | 75655628 | C | G | 0.1074 | 0.0161 | 2.89E-11 | -0.0068 | 0.0266 | 0.7973 |
| rs28999107 | 12 | 6493100 | T | G | 0.1083 | 0.0178 | 1.06E-09 | 0.0074 | 0.0269 | 0.784 |
| rs2948542 | 17 | 25856486 | G | A | 0.1016 | 0.0163 | 5.15E-10 | -0.0314 | 0.0274 | 0.2516 |
| rs3091315 | 17 | 32593665 | G | A | -0.1579 | 0.0182 | 3.76E-18 | 0.014 | 0.028 | 0.6175 |
| rs3122605 | 1 | 206955041 | A | G | -0.1748 | 0.0227 | 1.24E-14 | -0.0012 | 0.0366 | 0.9729 |
| rs3132558 | 6 | 31105466 | G | C | -0.1296 | 0.0186 | 3.31E-12 | -0.0698 | 0.03 | 0.01985 |
| rs34635748 | 12 | 40824663 | T | C | 0.4794 | 0.0504 | 1.95E-21 | 0.0554 | 0.0815 | 0.4972 |
| rs34687326 | 1 | 159799910 | A | G | -0.1649 | 0.0288 | 1.06E-08 | -0.0272 | 0.0414 | 0.5114 |
| rs35171809 | 6 | 167432766 | G | A | 0.1566 | 0.0159 | 9.07E-23 | 0.0185 | 0.0268 | 0.490399 |
| rs35730213 | 1 | 200874229 | C | G | -0.1166 | 0.0181 | 1.17E-10 | -0.0398 | 0.0324 | 0.2192 |
| rs3761158 | 20 | 44634912 | A | G | -0.1098 | 0.0165 | 2.65E-11 | -0.0104 | 0.0267 | 0.696 |
| rs3812609 | 9 | 139408892 | T | C | -0.1443 | 0.0247 | 4.96E-09 | -0.0829 | 0.0423 | 0.05024 |
| rs3816234 | 2 | 234185999 | A | G | 0.2704 | 0.0162 | 1.51E-62 | -0.024 | 0.0266 | 0.3675 |
| rs401775 | 6 | 31931137 | C | T | 0.2023 | 0.0203 | 2.17E-23 | 0.0561 | 0.0444 | 0.206 |
| rs4077515 | 9 | 139266496 | T | C | 0.1848 | 0.0162 | 3.14E-30 | -0.0063 | 0.0268 | 0.8148 |
| rs42861 | 16 | 28494421 | G | A | 0.1243 | 0.0167 | 8.87E-14 | -0.0138 | 0.0266 | 0.605701 |
| rs4343432 | 2 | 25161236 | G | A | 0.1123 | 0.0162 | 3.50E-12 | -0.0129 | 0.0272 | 0.6346 |
| rs4380956 | 8 | 126529074 | A | G | 0.132 | 0.0165 | 1.15E-15 | 0.0239 | 0.0296 | 0.4194 |
| rs4486887 | 16 | 50677571 | T | C | -0.1686 | 0.0172 | 1.37E-22 | 0.0251 | 0.0323 | 0.4372 |
| rs4655709 | 1 | 67903316 | A | G | 0.1224 | 0.0183 | 2.46E-11 | -0.0169 | 0.0363 | 0.641201 |
| rs4705885 | 5 | 130423559 | A | G | 0.1598 | 0.0174 | 4.21E-20 | -0.057 | 0.0303 | 0.059431 |
| rs4807570 | 19 | 1123652 | A | G | 0.1811 | 0.0193 | 6.03E-21 | 0.0384 | 0.0314 | 0.2209 |
| rs4821544 | 22 | 37258503 | C | T | 0.0966 | 0.0171 | 1.76E-08 | -0.0278 | 0.0327 | 0.3947 |
| rs492602 | 19 | 49206417 | G | A | 0.1084 | 0.0162 | 2.33E-11 | -0.0371 | 0.0274 | 0.1757 |
| rs55946629 | 2 | 43851246 | A | C | 0.1755 | 0.0231 | 2.85E-14 | 0.0001 | 0.049 | 0.9986 |
| rs56116661 | 3 | 188401160 | T | C | -0.1312 | 0.0212 | 5.67E-10 | -0.0137 | 0.0318 | 0.666801 |
| rs5754100 | 22 | 21916166 | C | T | 0.1687 | 0.0206 | 3.02E-16 | 0.0587 | 0.0291 | 0.04373 |
| rs59145923 | 16 | 50849720 | C | G | -0.1717 | 0.0309 | 2.72E-08 | -0.0111 | 0.0524 | 0.8318 |
| rs59926756 | 16 | 82879772 | A | G | 0.1062 | 0.0176 | 1.74E-09 | 0.032 | 0.0279 | 0.2507 |
| rs6062496 | 20 | 62329099 | A | G | 0.1223 | 0.0167 | 2.62E-13 | -0.0009 | 0.0278 | 0.9743 |
| rs61839660 | 10 | 6094697 | T | C | 0.1468 | 0.0261 | 1.98E-08 | -0.0785 | 0.067 | 0.2413 |
| rs62126620 | 19 | 33753200 | A | G | 0.144 | 0.0201 | 8.61E-13 | -0.0052 | 0.037 | 0.8874 |
| rs62228374 | 22 | 39698078 | A | G | 0.3164 | 0.0557 | 1.36E-08 | -0.0692 | 0.0722 | 0.338 |
| rs62324212 | 4 | 123560939 | A | C | 0.106 | 0.0163 | 8.02E-11 | 0.033 | 0.027 | 0.2222 |
| rs6416647 | 16 | 10965597 | C | T | 0.1007 | 0.0178 | 1.46E-08 | -0.0177 | 0.0282 | 0.5316 |
| rs6451494 | 5 | 40411291 | C | T | 0.2605 | 0.0166 | 8.26E-56 | -0.0022 | 0.0268 | 0.934 |
| rs6584282 | 10 | 101286495 | G | A | -0.1658 | 0.016 | 3.44E-25 | -0.0111 | 0.0265 | 0.674899 |
| rs6704109 | 1 | 172857050 | T | C | 0.1748 | 0.0181 | 5.10E-22 | -0.0117 | 0.0293 | 0.6887 |
| rs6740847 | 2 | 182308352 | G | A | -0.104 | 0.0161 | 9.72E-11 | -0.0021 | 0.0271 | 0.9388 |
| rs6808936 | 3 | 141109321 | G | A | 0.0904 | 0.0161 | 1.93E-08 | -0.0072 | 0.0266 | 0.7865 |
| rs7198678 | 16 | 50922656 | T | A | -0.1398 | 0.0229 | 1.08E-09 | -0.0382 | 0.0323 | 0.2372 |
| rs7206852 | 16 | 50395168 | A | T | -0.1287 | 0.0223 | 7.71E-09 | -0.0644 | 0.04 | 0.1075 |
| rs72743461 | 15 | 67441750 | A | C | 0.1684 | 0.0187 | 2.26E-19 | 0.0528 | 0.0301 | 0.079559 |
| rs72748445 | 5 | 40245696 | A | C | -0.1369 | 0.0181 | 4.31E-14 | -0.0134 | 0.0321 | 0.6767 |
| rs72798422 | 16 | 50866917 | C | T | 0.5495 | 0.0382 | 6.05E-47 | 0.0137 | 0.073 | 0.8508 |
| rs73243877 | 4 | 26047616 | G | A | 0.1164 | 0.0212 | 4.12E-08 | -0.0196 | 0.0381 | 0.6075 |
| rs744166 | 17 | 40514201 | G | A | -0.1142 | 0.0162 | 1.80E-12 | -0.0168 | 0.0268 | 0.5324 |
| rs7517847 | 1 | 67681669 | G | T | -0.3447 | 0.0165 | 5.84E-97 | -0.0328 | 0.0266 | 0.2177 |
| rs755374 | 5 | 158829294 | T | C | 0.1969 | 0.0174 | 1.38E-29 | 0.0267 | 0.029 | 0.3575 |
| rs7563433 | 2 | 231095678 | C | T | 0.1525 | 0.02 | 2.14E-14 | -0.0065 | 0.0392 | 0.8673 |
| rs7608697 | 2 | 61204641 | C | A | 0.1229 | 0.0163 | 4.03E-14 | -0.0065 | 0.0274 | 0.8113 |
| rs76532080 | 16 | 50488249 | T | C | 0.2939 | 0.0352 | 6.96E-17 | 0.0724 | 0.0782 | 0.355 |
| rs7753014 | 6 | 21441035 | G | C | -0.0989 | 0.0163 | 1.39E-09 | -0.04 | 0.0265 | 0.1307 |
| rs77566919 | 12 | 113163656 | A | G | -0.1089 | 0.0185 | 4.13E-09 | 0.0262 | 0.0312 | 0.4005 |
| rs79832570 | 8 | 145097720 | C | T | 0.2234 | 0.0344 | 8.90E-11 | 0.0568 | 0.0479 | 0.236 |
| rs80244186 | 13 | 42917861 | C | T | 0.1246 | 0.0226 | 3.66E-08 | 0.0482 | 0.0416 | 0.2473 |
| rs80262450 | 18 | 12818922 | A | G | 0.2268 | 0.0244 | 1.34E-20 | 0.0507 | 0.0407 | 0.2131 |
| rs9276772 | 6 | 32774291 | G | C | -0.1857 | 0.0269 | 5.15E-12 | 0.0513 | 0.0491 | 0.2961 |
| rs938650 | 8 | 129552540 | A | G | -0.1747 | 0.0247 | 1.65E-12 | 0.0043 | 0.0415 | 0.9177 |
| rs9482770 | 6 | 127443092 | C | T | 0.0987 | 0.0162 | 1.01E-09 | 0.0948 | 0.0268 | 0.000393 |
| rs9501109 | 6 | 31392118 | G | A | 0.1381 | 0.0209 | 3.84E-11 | -0.0781 | 0.0379 | 0.03931 |
| rs9501641 | 6 | 32450319 | T | C | 0.3027 | 0.0432 | 2.57E-12 | -0.026 | 0.073 | 0.721601 |
| rs9637870 | 5 | 150227615 | A | G | 0.2558 | 0.0275 | 1.33E-20 | 0.0313 | 0.0478 | 0.5128 |
| rs9656588 | 7 | 50306780 | C | T | 0.1183 | 0.0173 | 8.73E-12 | 0.0106 | 0.0292 | 0.716101 |
| rs9836291 | 3 | 49697459 | A | G | 0.1722 | 0.017 | 3.77E-24 | -0.0035 | 0.0269 | 0.8966 |

CD, Crohn's disease; AP, Acute pancreatitis; MR Mendelian randomization

Table S4 Summary information on the SNPs used as genetic instruments for the CD in MR study in irritable bowel syndrome

| SNP | chr | pos | A1 | A2 | Exposure (CD) | | | Outcome (IBS) | | |
| --- | --- | --- | --- | --- | --- | --- | --- | --- | --- | --- |
|  |  |  |  |  | β | Se | *P* | β | Se | *P* |
| rs10052709 | 5 | 158760477 | G | C | -0.141 | 0.0242 | 5.76E-09 | -0.0139 | 0.0393 | 0.723601 |
| rs10055349 | 5 | 40441718 | A | G | 0.1734 | 0.019 | 5.59E-20 | 0.0007 | 0.0257 | 0.9794 |
| rs10114470 | 9 | 117547772 | C | T | 0.1687 | 0.0177 | 1.76E-21 | -0.0125 | 0.0256 | 0.6254 |
| rs1012636 | 6 | 20674811 | T | G | 0.1291 | 0.0198 | 7.01E-11 | 0.0044 | 0.0244 | 0.8553 |
| rs10822050 | 10 | 64438771 | C | T | 0.1827 | 0.0162 | 2.35E-29 | 0.0387 | 0.0221 | 0.0801 |
| rs10884966 | 10 | 112185596 | A | G | 0.1131 | 0.0171 | 4.13E-11 | 0.0276 | 0.0224 | 0.2179 |
| rs11236797 | 11 | 76299649 | A | C | 0.176 | 0.0161 | 8.51E-28 | 0.0257 | 0.0219 | 0.2401 |
| rs114802258 | 1 | 160831855 | T | C | -0.2245 | 0.0384 | 5.11E-09 | 0.0197 | 0.0454 | 0.6648 |
| rs1148246 | 10 | 35496626 | T | C | -0.1323 | 0.0167 | 2.09E-15 | 0.028 | 0.0223 | 0.2081 |
| rs1157509 | 5 | 158786110 | G | A | 0.1519 | 0.0224 | 1.26E-11 | 0.0038 | 0.0298 | 0.8981 |
| rs11677002 | 2 | 28614401 | C | T | -0.1124 | 0.0163 | 4.57E-12 | 0.0003 | 0.0217 | 0.9892 |
| rs11683692 | 2 | 145509615 | C | T | -0.2144 | 0.038 | 1.75E-08 | -0.0148 | 0.042 | 0.723901 |
| rs11965964 | 6 | 33020604 | T | C | 0.3044 | 0.0529 | 8.83E-09 | 0.0797 | 0.133 | 0.5489 |
| rs1250573 | 10 | 81042475 | A | G | -0.1522 | 0.0179 | 1.92E-17 | 0.0054 | 0.0224 | 0.8101 |
| rs1260326 | 2 | 27730940 | C | T | -0.1053 | 0.0161 | 6.32E-11 | -0.047 | 0.0226 | 0.038 |
| rs12936409 | 17 | 38043649 | T | C | 0.1426 | 0.016 | 4.31E-19 | -0.0223 | 0.0216 | 0.3005 |
| rs1297264 | 21 | 16816017 | G | A | -0.1769 | 0.0163 | 1.59E-27 | -0.0109 | 0.022 | 0.619599 |
| rs13107325 | 4 | 103188709 | T | C | 0.2006 | 0.0284 | 1.66E-12 | -0.0433 | 0.0907 | 0.6331 |
| rs1321859 | 6 | 91011673 | T | C | -0.1049 | 0.0172 | 1.18E-09 | -0.0085 | 0.0252 | 0.737801 |
| rs1373904 | 13 | 44475398 | G | A | 0.141 | 0.0189 | 9.11E-14 | 0.0055 | 0.0269 | 0.8389 |
| rs142770866 | 19 | 10525372 | A | G | 0.1753 | 0.0292 | 1.99E-09 | 0.0681 | 0.0398 | 0.08679 |
| rs144309607 | 19 | 10492274 | T | C | -0.3712 | 0.047 | 2.69E-15 | 0.0248 | 0.0631 | 0.6939 |
| rs145126485 | 16 | 50918662 | C | A | 0.5236 | 0.0431 | 6.40E-34 | -0.0244 | 0.0682 | 0.7202 |
| rs145568234 | 6 | 32247045 | G | T | 0.8602 | 0.0633 | 4.31E-42 | 0.3404 | 0.1955 | 0.08163 |
| rs151175749 | 2 | 173424407 | G | C | 0.2483 | 0.0428 | 6.72E-09 | -0.084 | 0.0643 | 0.1911 |
| rs1583792 | 2 | 198900288 | T | C | -0.0882 | 0.016 | 3.26E-08 | -0.0204 | 0.0219 | 0.3521 |
| rs1633043 | 6 | 29732394 | G | A | -0.1266 | 0.0219 | 7.11E-09 | 0.0314 | 0.0269 | 0.2419 |
| rs181826 | 5 | 141526057 | A | C | 0.1162 | 0.0167 | 3.24E-12 | 0.0014 | 0.0227 | 0.9518 |
| rs1870148 | 10 | 82271341 | A | G | 0.1351 | 0.0206 | 5.44E-11 | 0.0894 | 0.0256 | 0.000486 |
| rs1887428 | 9 | 4984530 | C | G | -0.166 | 0.0169 | 8.54E-23 | 0.0235 | 0.0222 | 0.2892 |
| rs194746 | 14 | 69282887 | T | C | 0.0975 | 0.0161 | 1.24E-09 | 0.0087 | 0.0216 | 0.687 |
| rs1990684 | 16 | 50427554 | T | C | -0.1556 | 0.028 | 2.73E-08 | -0.007 | 0.0301 | 0.8148 |
| rs2002695 | 10 | 30805480 | G | A | -0.1293 | 0.0189 | 8.31E-12 | 0.0204 | 0.0236 | 0.389 |
| rs2021511 | 16 | 11344903 | T | C | -0.1082 | 0.0182 | 2.63E-09 | 0.0119 | 0.0244 | 0.6257 |
| rs2076756 | 16 | 50756881 | G | A | 0.385 | 0.0174 | ###### | 0.0244 | 0.03 | 0.4157 |
| rs2110735 | 2 | 103050925 | G | A | -0.1372 | 0.0185 | 1.20E-13 | 0.0378 | 0.0273 | 0.1665 |
| rs212408 | 6 | 159470242 | T | G | -0.1136 | 0.0167 | 9.12E-12 | -0.0497 | 0.0236 | 0.03517 |
| rs2143178 | 22 | 39660829 | C | T | -0.2087 | 0.0223 | 6.84E-21 | -0.0131 | 0.0335 | 0.6951 |
| rs2188962 | 5 | 131770805 | T | C | 0.2004 | 0.016 | 5.59E-36 | -0.0012 | 0.0231 | 0.9576 |
| rs2284553 | 21 | 34776695 | G | A | 0.1277 | 0.0165 | 1.14E-14 | -0.0061 | 0.0223 | 0.783401 |
| rs2476601 | 1 | 114377568 | G | A | 0.2312 | 0.0286 | 6.44E-16 | -0.0536 | 0.0302 | 0.07544 |
| rs2523989 | 6 | 30078275 | T | C | -0.1693 | 0.0254 | 2.46E-11 | -0.0057 | 0.0308 | 0.8523 |
| rs2581828 | 3 | 53133149 | G | C | -0.0941 | 0.0162 | 6.46E-09 | -0.0435 | 0.0222 | 0.0499 |
| rs2675670 | 10 | 75655628 | C | G | 0.1074 | 0.0161 | 2.89E-11 | 0.0351 | 0.0216 | 0.1044 |
| rs28999107 | 12 | 6493100 | T | G | 0.1083 | 0.0178 | 1.06E-09 | 0.0421 | 0.0218 | 0.05375 |
| rs2948542 | 17 | 25856486 | G | A | 0.1016 | 0.0163 | 5.15E-10 | 0.0297 | 0.0223 | 0.1834 |
| rs3091315 | 17 | 32593665 | G | A | -0.1579 | 0.0182 | 3.76E-18 | -0.0232 | 0.0228 | 0.3084 |
| rs3122605 | 1 | 206955041 | A | G | -0.1748 | 0.0227 | 1.24E-14 | 0.0107 | 0.0299 | 0.7217 |
| rs3132558 | 6 | 31105466 | G | C | -0.1296 | 0.0186 | 3.31E-12 | -0.056 | 0.0244 | 0.02197 |
| rs34635748 | 12 | 40824663 | T | C | 0.4794 | 0.0504 | 1.95E-21 | 0.0193 | 0.0676 | 0.7752 |
| rs34687326 | 1 | 159799910 | A | G | -0.1649 | 0.0288 | 1.06E-08 | 0.0318 | 0.0335 | 0.3423 |
| rs35171809 | 6 | 167432766 | G | A | 0.1566 | 0.0159 | 9.07E-23 | 0.0181 | 0.0218 | 0.4067 |
| rs35730213 | 1 | 200874229 | C | G | -0.1166 | 0.0181 | 1.17E-10 | -0.0277 | 0.0263 | 0.2923 |
| rs3761158 | 20 | 44634912 | A | G | -0.1098 | 0.0165 | 2.65E-11 | -0.0208 | 0.0217 | 0.3369 |
| rs3812609 | 9 | 139408892 | T | C | -0.1443 | 0.0247 | 4.96E-09 | -0.024 | 0.0341 | 0.4823 |
| rs3816234 | 2 | 234185999 | A | G | 0.2704 | 0.0162 | 1.51E-62 | 0.0043 | 0.0217 | 0.8435 |
| rs401775 | 6 | 31931137 | C | T | 0.2023 | 0.0203 | 2.17E-23 | 0.0926 | 0.0361 | 0.01027 |
| rs4077515 | 9 | 139266496 | T | C | 0.1848 | 0.0162 | 3.14E-30 | -0.0189 | 0.0218 | 0.3863 |
| rs42861 | 16 | 28494421 | G | A | 0.1243 | 0.0167 | 8.87E-14 | 0.0356 | 0.0216 | 0.099669 |
| rs4343432 | 2 | 25161236 | G | A | 0.1123 | 0.0162 | 3.5E-12 | 0.0123 | 0.0222 | 0.5798 |
| rs4380956 | 8 | 126529074 | A | G | 0.132 | 0.0165 | 1.15E-15 | -0.0116 | 0.024 | 0.630199 |
| rs4486887 | 16 | 50677571 | T | C | -0.1686 | 0.0172 | 1.37E-22 | -0.039 | 0.0263 | 0.1376 |
| rs4655709 | 1 | 67903316 | A | G | 0.1224 | 0.0183 | 2.46E-11 | -0.0014 | 0.0294 | 0.9623 |
| rs4705885 | 5 | 130423559 | A | G | 0.1598 | 0.0174 | 4.21E-20 | 0.0034 | 0.0245 | 0.8899 |
| rs4807570 | 19 | 1123652 | A | G | 0.1811 | 0.0193 | 6.03E-21 | 0.0103 | 0.0256 | 0.6886 |
| rs4821544 | 22 | 37258503 | C | T | 0.0966 | 0.0171 | 1.76E-08 | -0.0191 | 0.0265 | 0.4709 |
| rs492602 | 19 | 49206417 | G | A | 0.1084 | 0.0162 | 2.33E-11 | 0.0334 | 0.0222 | 0.1328 |
| rs55946629 | 2 | 43851246 | A | C | 0.1755 | 0.0231 | 2.85E-14 | 0.0567 | 0.0394 | 0.1503 |
| rs56116661 | 3 | 188401160 | T | C | -0.1312 | 0.0212 | 5.67E-10 | -0.0562 | 0.0258 | 0.02907 |
| rs5754100 | 22 | 21916166 | C | T | 0.1687 | 0.0206 | 3.02E-16 | -0.0442 | 0.0237 | 0.06269 |
| rs59145923 | 16 | 50849720 | C | G | -0.1717 | 0.0309 | 2.72E-08 | 0.0009 | 0.0426 | 0.984 |
| rs59926756 | 16 | 82879772 | A | G | 0.1062 | 0.0176 | 1.74E-09 | 0.0121 | 0.0227 | 0.5955 |
| rs6062496 | 20 | 62329099 | A | G | 0.1223 | 0.0167 | 2.62E-13 | 0.0266 | 0.0225 | 0.2376 |
| rs61839660 | 10 | 6094697 | T | C | 0.1468 | 0.0261 | 1.98E-08 | 0.0246 | 0.0543 | 0.6505 |
| rs62126620 | 19 | 33753200 | A | G | 0.144 | 0.0201 | 8.61E-13 | 0.0089 | 0.0301 | 0.7681 |
| rs62228374 | 22 | 39698078 | A | G | 0.3164 | 0.0557 | 1.36E-08 | -0.0301 | 0.059 | 0.61 |
| rs62324212 | 4 | 123560939 | A | C | 0.106 | 0.0163 | 8.02E-11 | -0.0126 | 0.022 | 0.5659 |
| rs6416647 | 16 | 10965597 | C | T | 0.1007 | 0.0178 | 1.46E-08 | 0.0022 | 0.0229 | 0.9236 |
| rs6451494 | 5 | 40411291 | C | T | 0.2605 | 0.0166 | 8.26E-56 | 0.0235 | 0.0218 | 0.2795 |
| rs6584282 | 10 | 101286495 | G | A | -0.1658 | 0.016 | 3.44E-25 | -0.0133 | 0.0215 | 0.5366 |
| rs6704109 | 1 | 172857050 | T | C | 0.1748 | 0.0181 | 5.1E-22 | 0.0123 | 0.0239 | 0.605701 |
| rs6740847 | 2 | 182308352 | G | A | -0.104 | 0.0161 | 9.72E-11 | -0.0307 | 0.022 | 0.1624 |
| rs6808936 | 3 | 141109321 | G | A | 0.0904 | 0.0161 | 1.93E-08 | -0.0046 | 0.0216 | 0.8335 |
| rs7198678 | 16 | 50922656 | T | A | -0.1398 | 0.0229 | 1.08E-09 | 0.0288 | 0.0263 | 0.2732 |
| rs7206852 | 16 | 50395168 | A | T | -0.1287 | 0.0223 | 7.71E-09 | 0.044 | 0.0325 | 0.1755 |
| rs72743461 | 15 | 67441750 | A | C | 0.1684 | 0.0187 | 2.26E-19 | -0.0094 | 0.0245 | 0.7008 |
| rs72748445 | 5 | 40245696 | A | C | -0.1369 | 0.0181 | 4.31E-14 | 0.0091 | 0.0261 | 0.725499 |
| rs72798422 | 16 | 50866917 | C | T | 0.5495 | 0.0382 | 6.05E-47 | -0.0512 | 0.0594 | 0.3888 |
| rs73243877 | 4 | 26047616 | G | A | 0.1164 | 0.0212 | 4.12E-08 | -0.0016 | 0.0308 | 0.9579 |
| rs744166 | 17 | 40514201 | G | A | -0.1142 | 0.0162 | 1.8E-12 | 0.0069 | 0.0218 | 0.752899 |
| rs7517847 | 1 | 67681669 | G | T | -0.3447 | 0.0165 | 5.84E-97 | -0.0156 | 0.0216 | 0.4711 |
| rs755374 | 5 | 158829294 | T | C | 0.1969 | 0.0174 | 1.38E-29 | -0.0145 | 0.0236 | 0.5373 |
| rs7563433 | 2 | 231095678 | C | T | 0.1525 | 0.02 | 2.14E-14 | -0.002 | 0.0317 | 0.9507 |
| rs7608697 | 2 | 61204641 | C | A | 0.1229 | 0.0163 | 4.03E-14 | 0.0235 | 0.0223 | 0.2911 |
| rs76532080 | 16 | 50488249 | T | C | 0.2939 | 0.0352 | 6.96E-17 | 0.0281 | 0.0627 | 0.653501 |
| rs7753014 | 6 | 21441035 | G | C | -0.0989 | 0.0163 | 1.39E-09 | -0.0155 | 0.0215 | 0.4708 |
| rs77566919 | 12 | 113163656 | A | G | -0.1089 | 0.0185 | 4.13E-09 | 0.0029 | 0.0252 | 0.9087 |
| rs79832570 | 8 | 145097720 | C | T | 0.2234 | 0.0344 | 8.9E-11 | 0.0052 | 0.0393 | 0.8942 |
| rs80244186 | 13 | 42917861 | C | T | 0.1246 | 0.0226 | 3.66E-08 | 0.0239 | 0.034 | 0.482 |
| rs80262450 | 18 | 12818922 | A | G | 0.2268 | 0.0244 | 1.34E-20 | 0.008 | 0.033 | 0.8093 |
| rs9276772 | 6 | 32774291 | G | C | -0.1857 | 0.0269 | 5.15E-12 | 0.0269 | 0.0397 | 0.4982 |
| rs938650 | 8 | 129552540 | A | G | -0.1747 | 0.0247 | 1.65E-12 | 0.0558 | 0.0338 | 0.0992 |
| rs9482770 | 6 | 127443092 | C | T | 0.0987 | 0.0162 | 1.01E-09 | -0.0351 | 0.0217 | 0.1052 |
| rs9501109 | 6 | 31392118 | G | A | 0.1381 | 0.0209 | 3.84E-11 | 0.0166 | 0.0309 | 0.590999 |
| rs9501641 | 6 | 32450319 | T | C | 0.3027 | 0.0432 | 2.57E-12 | -0.0921 | 0.0593 | 0.1201 |
| rs9637870 | 5 | 150227615 | A | G | 0.2558 | 0.0275 | 1.33E-20 | -0.0126 | 0.0388 | 0.745 |
| rs9656588 | 7 | 50306780 | C | T | 0.1183 | 0.0173 | 8.73E-12 | -0.0022 | 0.0237 | 0.9254 |
| rs9836291 | 3 | 49697459 | A | G | 0.1722 | 0.017 | 3.77E-24 | -0.0234 | 0.022 | 0.2867 |

; CD, Crohn's disease IBS Irritable, bowel syndrome; MR Mendelian randomization

Table S5 Summary information on the SNPs used as genetic instruments for the CD in MR study in Gastroesophageal reflux disease

| SNP | chr | pos | A1 | A2 | Exposure (CD) | | | Outcome (GERD) | | |
| --- | --- | --- | --- | --- | --- | --- | --- | --- | --- | --- |
|  |  |  |  |  | β | Se | *P* | β | Se | *P* |
| rs10052709 | 5 | 158760477 | G | C | -0.141 | 0.0242 | 5.76E-09 | 8.17E-05 | 0.006901 | 0.990556 |
| rs10114470 | 9 | 117547772 | C | T | 0.1687 | 0.0177 | 1.76E-21 | 0.012401 | 0.00516 | 0.016251 |
| rs10884966 | 10 | 112185596 | A | G | 0.1131 | 0.0171 | 4.13E-11 | 0.001636 | 0.005045 | 0.745762 |
| rs1157509 | 5 | 158786110 | G | A | 0.1519 | 0.0224 | 1.26E-11 | -0.00174 | 0.006399 | 0.785315 |
| rs11683692 | 2 | 145509615 | C | T | -0.2144 | 0.038 | 1.75E-08 | 0.008473 | 0.011193 | 0.449082 |
| rs12041056 | 1 | 67627260 | T | C | 0.1284 | 0.0163 | 3.75E-15 | -0.00199 | 0.004894 | 0.684491 |
| rs1260326 | 2 | 27730940 | C | T | -0.1053 | 0.0161 | 6.32E-11 | -0.00381 | 0.004922 | 0.438897 |
| rs13107325 | 4 | 103188709 | T | C | 0.2006 | 0.0284 | 1.66E-12 | 0.070144 | 0.009183 | 2.2E-14 |
| rs1321859 | 6 | 91011673 | T | C | -0.1049 | 0.0172 | 1.18E-09 | 0.003562 | 0.005052 | 0.480772 |
| rs1373904 | 13 | 44475398 | G | A | 0.141 | 0.0189 | 9.11E-14 | 0.000287 | 0.005956 | 0.961515 |
| rs181826 | 5 | 141526057 | A | C | 0.1162 | 0.0167 | 3.24E-12 | 0.010389 | 0.004942 | 0.035524 |
| rs1870148 | 10 | 82271341 | A | G | 0.1351 | 0.0206 | 5.44E-11 | -0.00373 | 0.005906 | 0.527157 |
| rs2076756 | 16 | 50756881 | G | A | 0.385 | 0.0174 | ###### | -0.00308 | 0.00556 | 0.579931 |
| rs2110735 | 2 | 103050925 | G | A | -0.1372 | 0.0185 | 1.20E-13 | -0.00891 | 0.005781 | 0.123411 |
| rs2143178 | 22 | 39660829 | C | T | -0.2087 | 0.0223 | 6.84E-21 | 0.008649 | 0.006511 | 0.18408 |
| rs2188962 | 5 | 131770805 | T | C | 0.2004 | 0.016 | 5.59E-36 | -0.00933 | 0.004854 | 0.054727 |
| rs2284553 | 21 | 34776695 | G | A | 0.1277 | 0.0165 | 1.14E-14 | 0.004389 | 0.004907 | 0.371131 |
| rs2476601 | 1 | 114377568 | G | A | 0.2312 | 0.0286 | 6.44E-16 | -0.01426 | 0.007932 | 0.072151 |
| rs2581828 | 3 | 53133149 | G | C | -0.0941 | 0.0162 | 6.46E-09 | 0.012488 | 0.004891 | 0.010672 |
| rs2838517 | 21 | 45613825 | C | T | -0.1456 | 0.0162 | 2.03E-19 | -0.00369 | 0.004959 | 0.456487 |
| rs2948542 | 17 | 25856486 | G | A | 0.1016 | 0.0163 | 5.15E-10 | -0.00222 | 0.004888 | 0.649835 |
| rs3091315 | 17 | 32593665 | G | A | -0.1579 | 0.0182 | 3.76E-18 | -0.00617 | 0.00538 | 0.251571 |
| rs3132558 | 6 | 31105466 | G | C | -0.1296 | 0.0186 | 3.31E-12 | -0.00407 | 0.005552 | 0.463243 |
| rs3850378 | 14 | 88417517 | C | T | 0.199 | 0.0267 | 8.31E-14 | 0.010059 | 0.008138 | 0.216462 |
| rs4077515 | 9 | 139266496 | T | C | 0.1848 | 0.0162 | 3.14E-30 | -0.00299 | 0.004866 | 0.538202 |
| rs4380956 | 8 | 126529074 | A | G | 0.132 | 0.0165 | 1.15E-15 | 0.006042 | 0.004951 | 0.22226 |
| rs4486887 | 16 | 50677571 | T | C | -0.1686 | 0.0172 | 1.37E-22 | -0.01177 | 0.005187 | 0.023304 |
| rs4821544 | 22 | 37258503 | C | T | 0.0966 | 0.0171 | 1.76E-08 | -0.00185 | 0.005223 | 0.723329 |
| rs5754100 | 22 | 21916166 | C | T | 0.1687 | 0.0206 | 3.02E-16 | -0.0093 | 0.006189 | 0.13275 |
| rs6416647 | 16 | 10965597 | C | T | 0.1007 | 0.0178 | 1.46E-08 | -0.00446 | 0.005319 | 0.401615 |
| rs6451494 | 5 | 40411291 | C | T | 0.2605 | 0.0166 | 8.26E-56 | 0.007752 | 0.004918 | 0.114939 |
| rs6740847 | 2 | 182308352 | G | A | -0.104 | 0.0161 | 9.72E-11 | 0.003013 | 0.004856 | 0.53496 |
| rs6808936 | 3 | 141109321 | G | A | 0.0904 | 0.0161 | 1.93E-08 | -0.00997 | 0.004837 | 0.039304 |
| rs7198678 | 16 | 50922656 | T | A | -0.1398 | 0.0229 | 1.08E-09 | -0.00646 | 0.006613 | 0.328754 |
| rs7206852 | 16 | 50395168 | A | T | -0.1287 | 0.0223 | 7.71E-09 | 0.004754 | 0.006626 | 0.473114 |
| rs744166 | 17 | 40514201 | G | A | -0.1142 | 0.0162 | 1.80E-12 | 0.004153 | 0.004855 | 0.392332 |
| rs7517847 | 1 | 67681669 | G | T | -0.3447 | 0.0165 | 5.84E-97 | -0.00319 | 0.004833 | 0.509614 |
| rs7563433 | 2 | 231095678 | C | T | 0.1525 | 0.02 | 2.14E-14 | 0.00213 | 0.006135 | 0.72843 |
| rs9482770 | 6 | 127443092 | C | T | 0.0987 | 0.0162 | 1.01E-09 | 0.004473 | 0.004833 | 0.354722 |

CD, Crohn's disease; GERD, Gastroesophageal reflux disease; MR Mendelian randomization

Table S6 Summary information on the SNPs used as genetic instruments for the CD in MR study in Cholelithiasis

| SNP | chr | pos | A1 | A2 | Exposure (CD) | | | Outcome (Cholelithiasis) | | |
| --- | --- | --- | --- | --- | --- | --- | --- | --- | --- | --- |
|  |  |  |  |  | β | Se | *P* | β | Se | *P* |
| rs10052709 | 5 | 158760477 | G | C | -0.141 | 0.0242 | 5.76E-09 | 0.0019 | 0.0219 | 0.9303 |
| rs10055349 | 5 | 40441718 | A | G | 0.1734 | 0.019 | 5.59E-20 | -0.0099 | 0.0143 | 0.4876 |
| rs10114470 | 9 | 117547772 | C | T | 0.1687 | 0.0177 | 1.76E-21 | 0.0087 | 0.0143 | 0.5433 |
| rs1012636 | 6 | 20674811 | T | G | 0.1291 | 0.0198 | 7.01E-11 | -0.0026 | 0.0136 | 0.8467 |
| rs10822050 | 10 | 64438771 | C | T | 0.1827 | 0.0162 | 2.35E-29 | 0.0072 | 0.0123 | 0.5574 |
| rs10884966 | 10 | 112185596 | A | G | 0.1131 | 0.0171 | 4.13E-11 | -0.0136 | 0.0125 | 0.2766 |
| rs11236797 | 11 | 76299649 | A | C | 0.176 | 0.0161 | 8.51E-28 | -0.0008 | 0.0122 | 0.946 |
| rs114802258 | 1 | 160831855 | T | C | -0.2245 | 0.0384 | 5.11E-09 | -0.0442 | 0.0251 | 0.07826 |
| rs1148246 | 10 | 35496626 | T | C | -0.1323 | 0.0167 | 2.09E-15 | 0.0094 | 0.0124 | 0.4478 |
| rs1157509 | 5 | 158786110 | G | A | 0.1519 | 0.0224 | 1.26E-11 | -0.0006 | 0.0165 | 0.9715 |
| rs11677002 | 2 | 28614401 | C | T | -0.1124 | 0.0163 | 4.57E-12 | 0.0295 | 0.0121 | 0.01456 |
| rs11683692 | 2 | 145509615 | C | T | -0.2144 | 0.038 | 1.75E-08 | 0.0084 | 0.0233 | 0.717599 |
| rs11965964 | 6 | 33020604 | T | C | 0.3044 | 0.0529 | 8.83E-09 | 0.0959 | 0.0735 | 0.1921 |
| rs1250573 | 10 | 81042475 | A | G | -0.1522 | 0.0179 | 1.92E-17 | -0.0112 | 0.0125 | 0.3707 |
| rs1260326 | 2 | 27730940 | C | T | -0.1053 | 0.0161 | 6.32E-11 | 0.0793 | 0.0126 | 3.46E-10 |
| rs12936409 | 17 | 38043649 | T | C | 0.1426 | 0.016 | 4.31E-19 | -0.0413 | 0.012 | 0.000604 |
| rs1297264 | 21 | 16816017 | G | A | -0.1769 | 0.0163 | 1.59E-27 | -0.0041 | 0.0122 | 0.736999 |
| rs13107325 | 4 | 103188709 | T | C | 0.2006 | 0.0284 | 1.66E-12 | -0.0004 | 0.0507 | 0.9944 |
| rs1321859 | 6 | 91011673 | T | C | -0.1049 | 0.0172 | 1.18E-09 | 0.0279 | 0.0141 | 0.04703 |
| rs1373904 | 13 | 44475398 | G | A | 0.141 | 0.0189 | 9.11E-14 | -0.0014 | 0.015 | 0.9264 |
| rs142770866 | 19 | 10525372 | A | G | 0.1753 | 0.0292 | 1.99E-09 | -0.0003 | 0.0221 | 0.9896 |
| rs144309607 | 19 | 10492274 | T | C | -0.3712 | 0.047 | 2.69E-15 | 0.0258 | 0.035 | 0.4607 |
| rs145126485 | 16 | 50918662 | C | A | 0.5236 | 0.0431 | 6.4E-34 | -0.0189 | 0.0381 | 0.6193 |
| rs145568234 | 6 | 32247045 | G | T | 0.8602 | 0.0633 | 4.31E-42 | 0.1403 | 0.1058 | 0.1849 |
| rs151175749 | 2 | 173424407 | G | C | 0.2483 | 0.0428 | 6.72E-09 | -0.0525 | 0.0359 | 0.1438 |
| rs1583792 | 2 | 198900288 | T | C | -0.0882 | 0.016 | 3.26E-08 | -0.0219 | 0.0122 | 0.072709 |
| rs1633043 | 6 | 29732394 | G | A | -0.1266 | 0.0219 | 7.11E-09 | 0.0463 | 0.015 | 0.00202 |
| rs181826 | 5 | 141526057 | A | C | 0.1162 | 0.0167 | 3.24E-12 | 0.032 | 0.0126 | 0.01137 |
| rs1870148 | 10 | 82271341 | A | G | 0.1351 | 0.0206 | 5.44E-11 | 0.0255 | 0.0143 | 0.07365 |
| rs1887428 | 9 | 4984530 | C | G | -0.166 | 0.0169 | 8.54E-23 | -0.0109 | 0.0124 | 0.3772 |
| rs194746 | 14 | 69282887 | T | C | 0.0975 | 0.0161 | 1.24E-09 | 0.0042 | 0.012 | 0.7286 |
| rs1990684 | 16 | 50427554 | T | C | -0.1556 | 0.028 | 2.73E-08 | -0.0024 | 0.0167 | 0.8882 |
| rs2002695 | 10 | 30805480 | G | A | -0.1293 | 0.0189 | 8.31E-12 | 0.0011 | 0.0132 | 0.9316 |
| rs2021511 | 16 | 11344903 | T | C | -0.1082 | 0.0182 | 2.63E-09 | -0.0191 | 0.0136 | 0.1612 |
| rs2076756 | 16 | 50756881 | G | A | 0.385 | 0.0174 | 1.8E-108 | 0.0002 | 0.0167 | 0.9898 |
| rs2110735 | 2 | 103050925 | G | A | -0.1372 | 0.0185 | 1.2E-13 | -0.0248 | 0.0152 | 0.102 |
| rs212408 | 6 | 159470242 | T | G | -0.1136 | 0.0167 | 9.12E-12 | 0.0162 | 0.0131 | 0.2165 |
| rs2143178 | 22 | 39660829 | C | T | -0.2087 | 0.0223 | 6.84E-21 | 0.002 | 0.0187 | 0.9131 |
| rs2188962 | 5 | 131770805 | T | C | 0.2004 | 0.016 | 5.59E-36 | -0.0181 | 0.0129 | 0.1587 |
| rs2284553 | 21 | 34776695 | G | A | 0.1277 | 0.0165 | 1.14E-14 | -0.0068 | 0.0124 | 0.585901 |
| rs2476601 | 1 | 114377568 | G | A | 0.2312 | 0.0286 | 6.44E-16 | -0.0303 | 0.0168 | 0.07249 |
| rs2523989 | 6 | 30078275 | T | C | -0.1693 | 0.0254 | 2.46E-11 | 0.0036 | 0.0171 | 0.8356 |
| rs2581828 | 3 | 53133149 | G | C | -0.0941 | 0.0162 | 6.46E-09 | 0.0167 | 0.0124 | 0.178 |
| rs2675670 | 10 | 75655628 | C | G | 0.1074 | 0.0161 | 2.89E-11 | 0.0263 | 0.012 | 0.02908 |
| rs28999107 | 12 | 6493100 | T | G | 0.1083 | 0.0178 | 1.06E-09 | 0.0076 | 0.0122 | 0.5313 |
| rs2948542 | 17 | 25856486 | G | A | 0.1016 | 0.0163 | 5.15E-10 | 0.009 | 0.0124 | 0.4703 |
| rs3091315 | 17 | 32593665 | G | A | -0.1579 | 0.0182 | 3.76E-18 | 0.0154 | 0.0127 | 0.2243 |
| rs3122605 | 1 | 206955041 | A | G | -0.1748 | 0.0227 | 1.24E-14 | 0.0171 | 0.0166 | 0.3027 |
| rs3132558 | 6 | 31105466 | G | C | -0.1296 | 0.0186 | 3.31E-12 | -0.0205 | 0.0136 | 0.1315 |
| rs34635748 | 12 | 40824663 | T | C | 0.4794 | 0.0504 | 1.95E-21 | 0.0452 | 0.0373 | 0.2251 |
| rs34687326 | 1 | 159799910 | A | G | -0.1649 | 0.0288 | 1.06E-08 | -0.0375 | 0.0186 | 0.04414 |
| rs35171809 | 6 | 167432766 | G | A | 0.1566 | 0.0159 | 9.07E-23 | 0.0075 | 0.0121 | 0.5343 |
| rs35730213 | 1 | 200874229 | C | G | -0.1166 | 0.0181 | 1.17E-10 | -0.0216 | 0.0146 | 0.1397 |
| rs3761158 | 20 | 44634912 | A | G | -0.1098 | 0.0165 | 2.65E-11 | 0.0097 | 0.0121 | 0.4234 |
| rs3812609 | 9 | 139408892 | T | C | -0.1443 | 0.0247 | 4.96E-09 | 0.0042 | 0.0191 | 0.8236 |
| rs3816234 | 2 | 234185999 | A | G | 0.2704 | 0.0162 | 1.51E-62 | -0.0404 | 0.0121 | 0.00083 |
| rs401775 | 6 | 31931137 | C | T | 0.2023 | 0.0203 | 2.17E-23 | 0.051 | 0.02 | 0.0107 |
| rs4077515 | 9 | 139266496 | T | C | 0.1848 | 0.0162 | 3.14E-30 | -0.0043 | 0.0121 | 0.719999 |
| rs42861 | 16 | 28494421 | G | A | 0.1243 | 0.0167 | 8.87E-14 | 0.0121 | 0.0121 | 0.3159 |
| rs4343432 | 2 | 25161236 | G | A | 0.1123 | 0.0162 | 3.5E-12 | -0.0035 | 0.0123 | 0.773599 |
| rs4380956 | 8 | 126529074 | A | G | 0.132 | 0.0165 | 1.15E-15 | 0.0075 | 0.0134 | 0.573499 |
| rs4486887 | 16 | 50677571 | T | C | -0.1686 | 0.0172 | 1.37E-22 | -0.0029 | 0.0146 | 0.8407 |
| rs4655709 | 1 | 67903316 | A | G | 0.1224 | 0.0183 | 2.46E-11 | -0.0191 | 0.0163 | 0.243 |
| rs4705885 | 5 | 130423559 | A | G | 0.1598 | 0.0174 | 4.21E-20 | -0.0147 | 0.0137 | 0.2825 |
| rs4807570 | 19 | 1123652 | A | G | 0.1811 | 0.0193 | 6.03E-21 | 0.0165 | 0.0143 | 0.2466 |
| rs4821544 | 22 | 37258503 | C | T | 0.0966 | 0.0171 | 1.76E-08 | -0.0365 | 0.0147 | 0.01311 |
| rs492602 | 19 | 49206417 | G | A | 0.1084 | 0.0162 | 2.33E-11 | 0.0421 | 0.0124 | 0.000667 |
| rs55946629 | 2 | 43851246 | A | C | 0.1755 | 0.0231 | 2.85E-14 | -0.0007 | 0.0221 | 0.975 |
| rs56116661 | 3 | 188401160 | T | C | -0.1312 | 0.0212 | 5.67E-10 | 0.0028 | 0.0144 | 0.8472 |
| rs5754100 | 22 | 21916166 | C | T | 0.1687 | 0.0206 | 3.02E-16 | 0.0464 | 0.0132 | 0.000432 |
| rs59145923 | 16 | 50849720 | C | G | -0.1717 | 0.0309 | 2.72E-08 | -0.0386 | 0.0238 | 0.1045 |
| rs59926756 | 16 | 82879772 | A | G | 0.1062 | 0.0176 | 1.74E-09 | 0.0137 | 0.0126 | 0.279 |
| rs6062496 | 20 | 62329099 | A | G | 0.1223 | 0.0167 | 2.62E-13 | -0.0246 | 0.0125 | 0.049741 |
| rs61839660 | 10 | 6094697 | T | C | 0.1468 | 0.0261 | 1.98E-08 | -0.0351 | 0.0301 | 0.2425 |
| rs62126620 | 19 | 33753200 | A | G | 0.144 | 0.0201 | 8.61E-13 | 0.0014 | 0.0167 | 0.9314 |
| rs62228374 | 22 | 39698078 | A | G | 0.3164 | 0.0557 | 1.36E-08 | 0.0374 | 0.0328 | 0.2529 |
| rs62324212 | 4 | 123560939 | A | C | 0.106 | 0.0163 | 8.02E-11 | 0.0124 | 0.0122 | 0.3124 |
| rs6416647 | 16 | 10965597 | C | T | 0.1007 | 0.0178 | 1.46E-08 | 0.0015 | 0.0128 | 0.9059 |
| rs6451494 | 5 | 40411291 | C | T | 0.2605 | 0.0166 | 8.26E-56 | -0.0168 | 0.0121 | 0.1665 |
| rs6584282 | 10 | 101286495 | G | A | -0.1658 | 0.016 | 3.44E-25 | 0.008 | 0.012 | 0.5036 |
| rs6704109 | 1 | 172857050 | T | C | 0.1748 | 0.0181 | 5.1E-22 | 0.0021 | 0.0133 | 0.8765 |
| rs6740847 | 2 | 182308352 | G | A | -0.104 | 0.0161 | 9.72E-11 | 0.0011 | 0.0122 | 0.9262 |
| rs6808936 | 3 | 141109321 | G | A | 0.0904 | 0.0161 | 1.93E-08 | 0.0118 | 0.0121 | 0.3291 |
| rs7198678 | 16 | 50922656 | T | A | -0.1398 | 0.0229 | 1.08E-09 | -0.0046 | 0.0146 | 0.751799 |
| rs7206852 | 16 | 50395168 | A | T | -0.1287 | 0.0223 | 7.71E-09 | 0.0059 | 0.0181 | 0.744201 |
| rs72743461 | 15 | 67441750 | A | C | 0.1684 | 0.0187 | 2.26E-19 | 0.006 | 0.0137 | 0.6624 |
| rs72748445 | 5 | 40245696 | A | C | -0.1369 | 0.0181 | 4.31E-14 | -0.031 | 0.0145 | 0.03292 |
| rs72798422 | 16 | 50866917 | C | T | 0.5495 | 0.0382 | 6.05E-47 | 0.0503 | 0.0331 | 0.1286 |
| rs73243877 | 4 | 26047616 | G | A | 0.1164 | 0.0212 | 4.12E-08 | 0.0163 | 0.0172 | 0.3414 |
| rs744166 | 17 | 40514201 | G | A | -0.1142 | 0.0162 | 1.8E-12 | -0.0035 | 0.0121 | 0.771999 |
| rs7517847 | 1 | 67681669 | G | T | -0.3447 | 0.0165 | 5.84E-97 | 0.0093 | 0.0121 | 0.4392 |
| rs755374 | 5 | 158829294 | T | C | 0.1969 | 0.0174 | 1.38E-29 | 0.0112 | 0.0131 | 0.3923 |
| rs7563433 | 2 | 231095678 | C | T | 0.1525 | 0.02 | 2.14E-14 | 0.0158 | 0.0177 | 0.3713 |
| rs7608697 | 2 | 61204641 | C | A | 0.1229 | 0.0163 | 4.03E-14 | -0.0149 | 0.0124 | 0.2292 |
| rs76532080 | 16 | 50488249 | T | C | 0.2939 | 0.0352 | 6.96E-17 | -0.0107 | 0.035 | 0.7591 |
| rs7753014 | 6 | 21441035 | G | C | -0.0989 | 0.0163 | 1.39E-09 | -0.016 | 0.012 | 0.1822 |
| rs77566919 | 12 | 113163656 | A | G | -0.1089 | 0.0185 | 4.13E-09 | -0.0172 | 0.0141 | 0.2219 |
| rs79832570 | 8 | 145097720 | C | T | 0.2234 | 0.0344 | 8.9E-11 | -0.0205 | 0.022 | 0.3499 |
| rs80244186 | 13 | 42917861 | C | T | 0.1246 | 0.0226 | 3.66E-08 | -0.0044 | 0.0189 | 0.8173 |
| rs80262450 | 18 | 12818922 | A | G | 0.2268 | 0.0244 | 1.34E-20 | 0 | 0.0184 | 0.9984 |
| rs9276772 | 6 | 32774291 | G | C | -0.1857 | 0.0269 | 5.15E-12 | -0.0049 | 0.0223 | 0.8257 |
| rs938650 | 8 | 129552540 | A | G | -0.1747 | 0.0247 | 1.65E-12 | 0.0136 | 0.0188 | 0.4693 |
| rs9482770 | 6 | 127443092 | C | T | 0.0987 | 0.0162 | 1.01E-09 | 0.0325 | 0.0121 | 0.007082 |
| rs9501109 | 6 | 31392118 | G | A | 0.1381 | 0.0209 | 3.84E-11 | 0.0089 | 0.0172 | 0.6052 |
| rs9501641 | 6 | 32450319 | T | C | 0.3027 | 0.0432 | 2.57E-12 | 0.0443 | 0.033 | 0.1788 |
| rs9637870 | 5 | 150227615 | A | G | 0.2558 | 0.0275 | 1.33E-20 | 0.0605 | 0.0216 | 0.004997 |
| rs9656588 | 7 | 50306780 | C | T | 0.1183 | 0.0173 | 8.73E-12 | 0.0142 | 0.0132 | 0.284 |
| rs9836291 | 3 | 49697459 | A | G | 0.1722 | 0.017 | 3.77E-24 | -0.0076 | 0.0122 | 0.5328 |

CD, Crohn's disease; MR, Mendelian randomization

Table S7 Summary information on the SNPs used as genetic instruments for the CD in MR study in celiac disease

| SNP | chr | pos | A1 | A2 | Exposure (UC) | | | Outcome (CeD) | | |
| --- | --- | --- | --- | --- | --- | --- | --- | --- | --- | --- |
|  |  |  |  |  | β | Se | *P* | β | Se | *P* |
| rs10114470 | 9 | 117547772 | C | T | 0.1687 | 0.0177 | 1.76E-21 | 0.0452066 | 0.0197851 | 0.0223198 |
| rs11236797 | 11 | 76299649 | A | C | 0.176 | 0.0161 | 8.51E-28 | 0.0440169 | 0.018348 | 0.0164399 |
| rs12936409 | 17 | 38043649 | T | C | 0.1426 | 0.016 | 4.31E-19 | -0.00702462 | 0.0184371 | 0.7032 |
| rs1297264 | 21 | 16816017 | G | A | -0.1769 | 0.0163 | 1.59E-27 | -0.0395728 | 0.0187074 | 0.0344001 |
| rs1321859 | 6 | 91011673 | T | C | -0.1049 | 0.0172 | 1.18E-09 | 0.0880109 | 0.0194776 | 6.23E-06 |
| rs1373904 | 13 | 44475398 | G | A | 0.141 | 0.0189 | 9.11E-14 | 0.0256677 | 0.022502 | 0.254 |
| rs194746 | 14 | 69282887 | T | C | 0.0975 | 0.0161 | 1.24E-09 | 0.0760347 | 0.0185968 | 4.34E-05 |
| rs2076756 | 16 | 50756881 | G | A | 0.385 | 0.0174 | 1.80E-108 | 0.0478373 | 0.0214579 | 0.0257899 |
| rs212409 | 6 | 159470058 | A | G | -0.1096 | 0.0162 | 1.49E-11 | -0.1441 | 0.0186154 | 9.87E-15 |
| rs2143178 | 22 | 39660829 | C | T | -0.2087 | 0.0223 | 6.84E-21 | 0.0217615 | 0.0247986 | 0.3802 |
| rs2188962 | 5 | 131770805 | T | C | 0.2004 | 0.016 | 5.59E-36 | 0.0582689 | 0.0189431 | 0.00209802 |
| rs2284553 | 21 | 34776695 | G | A | 0.1277 | 0.0165 | 1.14E-14 | -0.0009995 | 0.0155957 | 0.9489 |
| rs2838517 | 21 | 45613825 | C | T | -0.1456 | 0.0162 | 2.03E-19 | -0.0188218 | 0.0186703 | 0.3134 |
| rs3122605 | 1 | 206955041 | A | G | -0.1748 | 0.0227 | 1.24E-14 | 0.0277824 | 0.0264529 | 0.2936 |
| rs3816234 | 2 | 234185999 | A | G | 0.2704 | 0.0162 | 1.51E-62 | -0.0217615 | 0.0186232 | 0.2426 |
| rs4380956 | 8 | 126529074 | A | G | 0.132 | 0.0165 | 1.15E-15 | 0.0355236 | 0.0189082 | 0.0602795 |
| rs6062496 | 20 | 62329099 | A | G | 0.1223 | 0.0167 | 2.62E-13 | 0.00531409 | 0.0187711 | 0.7771 |
| rs61839660 | 10 | 6094697 | T | C | 0.1468 | 0.0261 | 1.98E-08 | 0.0601539 | 0.0309783 | 0.0521603 |
| rs6451494 | 5 | 40411291 | C | T | 0.2605 | 0.0166 | 8.26E-56 | 0.0282966 | 0.0188588 | 0.1335 |
| rs6579807 | 5 | 150286845 | T | C | 0.1993 | 0.0244 | 3.44E-16 | 0.0314987 | 0.0267792 | 0.2395 |
| rs6584282 | 10 | 101286495 | G | A | -0.1658 | 0.016 | 3.44E-25 | 0.00120072 | 0.0186988 | 0.9488 |
| rs714910 | 17 | 32617265 | C | A | -0.1531 | 0.0181 | 2.49E-17 | 0.0109399 | 0.0202756 | 0.5895 |
| rs72743461 | 15 | 67441750 | A | C | 0.1684 | 0.0187 | 2.26E-19 | 0.08158 | 0.0214539 | 0.000143199 |
| rs73243877 | 4 | 26047616 | G | A | 0.1164 | 0.0212 | 4.12E-08 | 0.0198026 | 0.0243618 | 0.4163 |
| rs73516754 | 6 | 106459738 | C | A | 0.1423 | 0.0169 | 4.04E-17 | 0.0363319 | 0.019451 | 0.0617803 |
| rs744166 | 17 | 40514201 | G | A | -0.1142 | 0.0162 | 1.80E-12 | -0.00803217 | 0.0186857 | 0.6673 |
| rs755374 | 5 | 158829294 | T | C | 0.1969 | 0.0174 | 1.38E-29 | 0.0129162 | 0.0201975 | 0.5225 |
| rs9656588 | 7 | 50306780 | C | T | 0.1183 | 0.0173 | 8.73E-12 | 0.0296348 | 0.0200097 | 0.1386 |

CeD celiac disease

Table S8 Summary information on the SNPs used as genetic instruments for the UC in MR study in acute pancreatitis

| SNP | chr | pos | A1 | A2 | Exposure (UC) | | | Outcome (AP) | | |
| --- | --- | --- | --- | --- | --- | --- | --- | --- | --- | --- |
|  |  |  |  |  | β | Se | *P* | β | Se | *P* |
| rs10272963 | 7 | 107486902 | T | C | -0.1512 | 0.016 | 1.07E+08 | -0.0221 | 0.0266 | 0.4061 |
| rs10408351 | 19 | 33754044 | A | G | 0.1548 | 0.0204 | 3.38E+07 | 0.0288 | 0.0344 | 0.4034 |
| rs10737481 | 1 | 20171514 | G | T | 0.2173 | 0.0159 | 2.02E+07 | 0.0248 | 0.0265 | 0.349 |
| rs10761659 | 10 | 64445564 | G | A | 0.1276 | 0.016 | 6.44E+07 | -0.0045 | 0.0265 | 0.8642 |
| rs10817678 | 9 | 117579457 | A | G | 0.1332 | 0.017 | 1.18E+08 | 0.047 | 0.0284 | 0.097969 |
| rs11209026 | 1 | 67705958 | A | G | -0.483 | 0.0358 | 6.77E+07 | -0.048 | 0.0631 | 0.4474 |
| rs1131095 | 3 | 49714225 | C | T | 0.1593 | 0.0168 | 4.97E+07 | -0.0034 | 0.0269 | 0.8997 |
| rs113986290 | 6 | 19781009 | T | C | -0.3066 | 0.0531 | 1.98E+07 | -0.1536 | 0.0991 | 0.1213 |
| rs1157509 | 5 | 158786110 | G | A | 0.1311 | 0.0217 | 1.59E+08 | -0.0042 | 0.0368 | 0.9098 |
| rs11645239 | 16 | 23847062 | G | C | -0.1174 | 0.02 | 2.38E+07 | -0.0092 | 0.0361 | 0.799 |
| rs11651246 | 17 | 40759937 | G | T | 0.147 | 0.0219 | 4.08E+07 | 0.0193 | 0.0341 | 0.5716 |
| rs116724447 | 5 | 158581640 | A | G | -0.339 | 0.0572 | 1.59E+08 | -0.0453 | 0.0776 | 0.5595 |
| rs117292830 | 6 | 31218268 | A | G | 0.4948 | 0.0495 | 3.12E+07 | 0.1063 | 0.0704 | 0.131 |
| rs1265098 | 6 | 31106177 | C | T | -0.1646 | 0.0203 | 3.11E+07 | -0.0787 | 0.0304 | 0.009514 |
| rs12825700 | 12 | 68492980 | A | G | 0.1889 | 0.0161 | 6.85E+07 | -0.0004 | 0.0284 | 0.9887 |
| rs12936409 | 17 | 38043649 | T | C | 0.1365 | 0.0158 | 3.80E+07 | 0.0073 | 0.0265 | 0.7831 |
| rs1317209 | 1 | 20140036 | A | G | 0.1818 | 0.0203 | 2.01E+07 | 0.0494 | 0.0319 | 0.1213 |
| rs13200059 | 6 | 111943234 | A | G | 0.2944 | 0.0436 | 1.12E+08 | -0.1024 | 0.0617 | 0.09736 |
| rs1336900 | 1 | 150679033 | A | G | -0.0887 | 0.0163 | 1.51E+08 | -0.051 | 0.027 | 0.05851 |
| rs1359946 | 13 | 27536972 | A | G | 0.1571 | 0.0202 | 2.75E+07 | -0.0213 | 0.0348 | 0.5407 |
| rs137845 | 22 | 50439430 | G | A | 0.1011 | 0.0158 | 5.04E+07 | -0.0398 | 0.0267 | 0.1363 |
| rs138788 | 22 | 35729721 | A | G | 0.0896 | 0.0162 | 3.57E+07 | 0.0166 | 0.0267 | 0.5359 |
| rs1411262 | 9 | 5459419 | T | C | 0.101 | 0.0179 | 5.46E+06 | 0.0477 | 0.0297 | 0.1085 |
| rs141725002 | 6 | 30971976 | A | G | 0.4885 | 0.0883 | 3.10E+07 | -0.7237 | 0.4158 | 0.08175 |
| rs16830407 | 2 | 199625153 | A | G | 0.1078 | 0.0166 | 2.00E+08 | -0.0093 | 0.0269 | 0.728299 |
| rs17190351 | 6 | 31047448 | A | G | 0.4301 | 0.0561 | 3.10E+07 | -0.0122 | 0.1305 | 0.9255 |
| rs17202899 | 6 | 32434481 | C | T | 0.2211 | 0.0312 | 3.24E+07 | -0.0008 | 0.0556 | 0.9884 |
| rs1736161 | 21 | 16833222 | A | G | -0.1227 | 0.0161 | 1.68E+07 | 0.0235 | 0.0271 | 0.3856 |
| rs17656349 | 5 | 149605994 | T | C | 0.09 | 0.0159 | 1.50E+08 | 0.0313 | 0.0273 | 0.2516 |
| rs17715902 | 5 | 134451465 | A | G | 0.0974 | 0.0166 | 1.34E+08 | 0.0083 | 0.0278 | 0.766399 |
| rs1811711 | 2 | 228670476 | G | C | -0.1299 | 0.0223 | 2.29E+08 | 0.0082 | 0.0376 | 0.8277 |
| rs1846190 | 6 | 32583813 | A | G | -0.2267 | 0.019 | 3.26E+07 | 0.005 | 0.03 | 0.8689 |
| rs1887428 | 9 | 4984530 | C | G | -0.167 | 0.0166 | 4.98E+06 | 0.0019 | 0.0273 | 0.9435 |
| rs2045241 | 11 | 114428783 | A | G | -0.1063 | 0.0169 | 1.14E+08 | 0.01 | 0.0271 | 0.7114 |
| rs2212434 | 11 | 76281593 | T | C | 0.1252 | 0.0159 | 7.63E+07 | 0.0491 | 0.027 | 0.068309 |
| rs2294633 | 1 | 20090235 | T | C | -0.0985 | 0.0177 | 2.01E+07 | 0.0098 | 0.0279 | 0.726599 |
| rs2301989 | 7 | 107443871 | A | G | -0.1294 | 0.0161 | 1.07E+08 | -0.0455 | 0.0265 | 0.085961 |
| rs2816980 | 1 | 200078035 | T | G | 0.1941 | 0.026 | 2.00E+08 | 0.0335 | 0.0434 | 0.4401 |
| rs2836881 | 21 | 40466299 | T | G | -0.2217 | 0.0186 | 4.05E+07 | -0.0451 | 0.0308 | 0.1441 |
| rs3024493 | 1 | 206943968 | A | C | 0.21 | 0.0209 | 2.07E+08 | 0.0003 | 0.0364 | 0.9934 |
| rs3097666 | 6 | 33022788 | C | G | -0.2154 | 0.0304 | 3.30E+07 | 0.0733 | 0.053 | 0.1664 |
| rs3129299 | 6 | 32900787 | T | C | -0.14 | 0.0236 | 3.29E+07 | -0.0149 | 0.0459 | 0.746 |
| rs3130500 | 6 | 31119976 | A | T | -0.1184 | 0.0183 | 3.11E+07 | 0.0297 | 0.0308 | 0.3349 |
| rs34638686 | 3 | 48682658 | T | C | 0.1795 | 0.0262 | 4.87E+07 | -0.0254 | 0.04 | 0.5256 |
| rs34920465 | 1 | 22700351 | G | A | -0.1708 | 0.0213 | 2.27E+07 | 0.0427 | 0.0467 | 0.3604 |
| rs3812565 | 9 | 139272502 | C | T | 0.1335 | 0.016 | 1.39E+08 | -0.0076 | 0.0273 | 0.7807 |
| rs3820328 | 1 | 20142447 | G | A | -0.1662 | 0.0164 | 2.01E+07 | -0.013 | 0.0268 | 0.6282 |
| rs3823377 | 6 | 29944253 | A | C | 0.1482 | 0.0196 | 2.99E+07 | -0.0242 | 0.0274 | 0.3772 |
| rs41291790 | 6 | 31572664 | A | G | 0.9494 | 0.0806 | 3.16E+07 | 0.0272 | 0.0972 | 0.7798 |
| rs45593732 | 6 | 31583725 | T | C | 0.4903 | 0.0523 | 3.16E+07 | 0.0169 | 0.0607 | 0.780301 |
| rs4676408 | 2 | 241574401 | A | G | 0.1433 | 0.0167 | 2.42E+08 | 0.0047 | 0.0276 | 0.8642 |
| rs4711173 | 6 | 28514583 | C | T | 0.1166 | 0.0201 | 2.85E+07 | -0.0142 | 0.0303 | 0.6402 |
| rs4728142 | 7 | 128573967 | A | G | 0.0995 | 0.0158 | 1.29E+08 | -0.0337 | 0.0269 | 0.2109 |
| rs4993442 | 22 | 30253256 | T | G | -0.0988 | 0.0179 | 3.03E+07 | -0.0313 | 0.0293 | 0.2862 |
| rs56062135 | 15 | 67455630 | T | C | 0.1078 | 0.0184 | 6.75E+07 | 0.0516 | 0.0301 | 0.08609 |
| rs6017342 | 20 | 43065028 | C | A | 0.1944 | 0.017 | 4.31E+07 | 0.0139 | 0.0268 | 0.6033 |
| rs6062496 | 20 | 62329099 | A | G | 0.1359 | 0.0163 | 6.23E+07 | -0.0009 | 0.0278 | 0.9743 |
| rs62180181 | 2 | 199852010 | T | C | 0.1226 | 0.0171 | 2.00E+08 | -0.01 | 0.0274 | 0.7153 |
| rs6658353 | 1 | 161469054 | C | G | -0.1569 | 0.016 | 1.61E+08 | -0.0052 | 0.0264 | 0.8428 |
| rs67111717 | 5 | 176790162 | G | A | 0.0944 | 0.0171 | 1.77E+08 | -0.0099 | 0.0276 | 0.7193 |
| rs6889364 | 5 | 40347469 | A | G | 0.1318 | 0.0228 | 4.03E+07 | -0.0031 | 0.0335 | 0.9272 |
| rs6915986 | 6 | 32683898 | C | T | 0.404 | 0.0616 | 3.27E+07 | 0.5346 | 0.2811 | 0.05718 |
| rs6933404 | 6 | 137959235 | C | T | 0.1486 | 0.0188 | 1.38E+08 | 0.0465 | 0.0338 | 0.169 |
| rs7203363 | 16 | 68587692 | A | T | 0.1071 | 0.0189 | 6.86E+07 | 0.019 | 0.0346 | 0.5822 |
| rs72704802 | 5 | 554211 | T | C | -0.1223 | 0.0206 | 5.54E+05 | -0.0427 | 0.04 | 0.2857 |
| rs7523335 | 1 | 8180210 | A | G | -0.1389 | 0.021 | 8.18E+06 | 0 | 0.0299 | 1 |
| rs7544646 | 1 | 2496649 | G | C | -0.1168 | 0.016 | 2.50E+06 | 0.0159 | 0.0265 | 0.5497 |
| rs755374 | 5 | 158829294 | T | C | 0.1714 | 0.0171 | 1.59E+08 | 0.0267 | 0.029 | 0.3575 |
| rs7554511 | 1 | 200877562 | A | C | -0.1448 | 0.0178 | 2.01E+08 | -0.0435 | 0.0324 | 0.1794 |
| rs7608697 | 2 | 61204641 | C | A | 0.1597 | 0.0161 | 6.12E+07 | -0.0065 | 0.0274 | 0.8113 |
| rs77108272 | 6 | 31455675 | A | C | 0.5479 | 0.062 | 3.15E+07 | 0.0849 | 0.0802 | 0.2895 |
| rs78064630 | 19 | 10562802 | A | G | 0.1759 | 0.0308 | 1.06E+07 | 0.0322 | 0.0567 | 0.5706 |
| rs79051659 | 1 | 151757843 | A | G | 0.1605 | 0.0264 | 1.52E+08 | -0.0075 | 0.0521 | 0.8859 |
| rs7911117 | 10 | 27179596 | G | T | -0.1342 | 0.0239 | 2.72E+07 | -0.0162 | 0.0385 | 0.673899 |
| rs7911680 | 10 | 101293468 | C | A | -0.1525 | 0.0159 | 1.01E+08 | -0.0115 | 0.0265 | 0.664099 |
| rs798506 | 7 | 2788912 | C | T | -0.1206 | 0.0179 | 2.79E+06 | 0.0051 | 0.0279 | 0.8545 |
| rs8073117 | 17 | 70639396 | A | G | -0.1548 | 0.0225 | 7.06E+07 | 0.0274 | 0.0393 | 0.4864 |
| rs872956 | 6 | 33076090 | A | T | -0.1378 | 0.021 | 3.31E+07 | -0.0643 | 0.0329 | 0.05037 |
| rs9271176 | 6 | 32578127 | G | A | -0.3495 | 0.0173 | 3.26E+07 | -0.0248 | 0.0295 | 0.4013 |
| rs9611131 | 22 | 39662480 | C | T | -0.1494 | 0.0227 | 3.97E+07 | -0.0071 | 0.0413 | 0.8626 |

UC, Ulcerative colitis; AP, Acute pancreatitis; MR, Mendelian randomization

Table S9 Summary information on the SNPs used as genetic instruments for the UC in MR study in irritable bowel syndrome

| SNP | chr | pos | A1 | A2 | Exposure (UC) | | | Outcome (IBS) | | |
| --- | --- | --- | --- | --- | --- | --- | --- | --- | --- | --- |
|  |  |  |  |  | β | Se | *P* | β | Se | *P* |
| rs10272963 | 7 | 107486902 | T | C | -0.1512 | 0.016 | 1.07E+08 | 0.0036 | 0.0216 | 0.8671 |
| rs10408351 | 19 | 33754044 | A | G | 0.1548 | 0.0204 | 3.38E+07 | 0.0163 | 0.028 | 0.5604 |
| rs10737481 | 1 | 20171514 | G | T | 0.2173 | 0.0159 | 2.02E+07 | 0.0225 | 0.0215 | 0.2965 |
| rs10761659 | 10 | 64445564 | G | A | 0.1276 | 0.016 | 6.44E+07 | 0.0272 | 0.0215 | 0.2075 |
| rs10817678 | 9 | 117579457 | A | G | 0.1332 | 0.017 | 1.18E+08 | 0.0053 | 0.0231 | 0.8186 |
| rs11209026 | 1 | 67705958 | A | G | -0.483 | 0.0358 | 6.77E+07 | -0.0436 | 0.0519 | 0.4008 |
| rs1131095 | 3 | 49714225 | C | T | 0.1593 | 0.0168 | 4.97E+07 | -0.0233 | 0.022 | 0.2897 |
| rs113986290 | 6 | 19781009 | T | C | -0.3066 | 0.0531 | 1.98E+07 | 0.0019 | 0.0804 | 0.9811 |
| rs1157509 | 5 | 158786110 | G | A | 0.1311 | 0.0217 | 1.59E+08 | 0.0038 | 0.0298 | 0.8981 |
| rs11645239 | 16 | 23847062 | G | C | -0.1174 | 0.02 | 2.38E+07 | -0.0161 | 0.0293 | 0.581599 |
| rs11651246 | 17 | 40759937 | G | T | 0.147 | 0.0219 | 4.08E+07 | -0.0004 | 0.0277 | 0.9898 |
| rs116724447 | 5 | 158581640 | A | G | -0.339 | 0.0572 | 1.59E+08 | -0.0759 | 0.0636 | 0.2331 |
| rs117292830 | 6 | 31218268 | A | G | 0.4948 | 0.0495 | 3.12E+07 | 0.0854 | 0.057 | 0.1342 |
| rs1265098 | 6 | 31106177 | C | T | -0.1646 | 0.0203 | 3.11E+07 | -0.0228 | 0.0246 | 0.3534 |
| rs12825700 | 12 | 68492980 | A | G | 0.1889 | 0.0161 | 6.85E+07 | 0.0303 | 0.023 | 0.1872 |
| rs12936409 | 17 | 38043649 | T | C | 0.1365 | 0.0158 | 3.80E+07 | -0.0223 | 0.0216 | 0.3005 |
| rs1317209 | 1 | 20140036 | A | G | 0.1818 | 0.0203 | 2.01E+07 | 0.0199 | 0.0259 | 0.4424 |
| rs13200059 | 6 | 111943234 | A | G | 0.2944 | 0.0436 | 1.12E+08 | -0.0327 | 0.0504 | 0.5165 |
| rs1336900 | 1 | 150679033 | A | G | -0.0887 | 0.0163 | 1.51E+08 | 0.0112 | 0.0219 | 0.6096 |
| rs1359946 | 13 | 27536972 | A | G | 0.1571 | 0.0202 | 2.75E+07 | 0.0113 | 0.0282 | 0.6896 |
| rs137845 | 22 | 50439430 | G | A | 0.1011 | 0.0158 | 5.04E+07 | -0.0257 | 0.0217 | 0.2378 |
| rs138788 | 22 | 35729721 | A | G | 0.0896 | 0.0162 | 3.57E+07 | 0.0445 | 0.0217 | 0.04044 |
| rs1411262 | 9 | 5459419 | T | C | 0.101 | 0.0179 | 5.46E+06 | 0.0182 | 0.0242 | 0.4519 |
| rs141725002 | 6 | 30971976 | A | G | 0.4885 | 0.0883 | 3.10E+07 | 0.0187 | 0.3162 | 0.9529 |
| rs16830407 | 2 | 199625153 | A | G | 0.1078 | 0.0166 | 2.00E+08 | 0.0311 | 0.0218 | 0.1541 |
| rs17190351 | 6 | 31047448 | A | G | 0.4301 | 0.0561 | 3.10E+07 | 0.0129 | 0.1047 | 0.9022 |
| rs17202899 | 6 | 32434481 | C | T | 0.2211 | 0.0312 | 3.24E+07 | 0.0469 | 0.045 | 0.2974 |
| rs1736161 | 21 | 16833222 | A | G | -0.1227 | 0.0161 | 1.68E+07 | -0.0126 | 0.022 | 0.5654 |
| rs17656349 | 5 | 149605994 | T | C | 0.09 | 0.0159 | 1.50E+08 | 0.0141 | 0.0222 | 0.5251 |
| rs17715902 | 5 | 134451465 | A | G | 0.0974 | 0.0166 | 1.34E+08 | -0.0112 | 0.0226 | 0.619901 |
| rs1811711 | 2 | 228670476 | G | C | -0.1299 | 0.0223 | 2.29E+08 | -0.0509 | 0.0304 | 0.094511 |
| rs1846190 | 6 | 32583813 | A | G | -0.2267 | 0.019 | 3.26E+07 | -0.0357 | 0.0244 | 0.1434 |
| rs1887428 | 9 | 4984530 | C | G | -0.167 | 0.0166 | 4.98E+06 | 0.0235 | 0.0222 | 0.2892 |
| rs2045241 | 11 | 114428783 | A | G | -0.1063 | 0.0169 | 1.14E+08 | 0.0488 | 0.0221 | 0.02701 |
| rs2212434 | 11 | 76281593 | T | C | 0.1252 | 0.0159 | 7.63E+07 | 0.0248 | 0.0219 | 0.257 |
| rs2294633 | 1 | 20090235 | T | C | -0.0985 | 0.0177 | 2.01E+07 | 0.0188 | 0.0227 | 0.4082 |
| rs2301989 | 7 | 107443871 | A | G | -0.1294 | 0.0161 | 1.07E+08 | -0.0211 | 0.0216 | 0.3286 |
| rs2816980 | 1 | 200078035 | T | G | 0.1941 | 0.026 | 2.00E+08 | -0.0263 | 0.0353 | 0.4574 |
| rs2836881 | 21 | 40466299 | T | G | -0.2217 | 0.0186 | 4.05E+07 | -0.0029 | 0.0249 | 0.9082 |
| rs3024493 | 1 | 206943968 | A | C | 0.21 | 0.0209 | 2.07E+08 | -0.0216 | 0.0298 | 0.469 |
| rs3097666 | 6 | 33022788 | C | G | -0.2154 | 0.0304 | 3.30E+07 | -0.0672 | 0.043 | 0.1184 |
| rs3129299 | 6 | 32900787 | T | C | -0.14 | 0.0236 | 3.29E+07 | 0.0362 | 0.0372 | 0.3306 |
| rs3130500 | 6 | 31119976 | A | T | -0.1184 | 0.0183 | 3.11E+07 | -0.0023 | 0.025 | 0.9265 |
| rs34638686 | 3 | 48682658 | T | C | 0.1795 | 0.0262 | 4.87E+07 | 0.0905 | 0.0328 | 0.005788 |
| rs34920465 | 1 | 22700351 | G | A | -0.1708 | 0.0213 | 2.27E+07 | -0.0351 | 0.0375 | 0.3485 |
| rs3812565 | 9 | 139272502 | C | T | 0.1335 | 0.016 | 1.39E+08 | -0.0059 | 0.0222 | 0.7906 |
| rs3820328 | 1 | 20142447 | G | A | -0.1662 | 0.0164 | 2.01E+07 | -0.0048 | 0.0218 | 0.8268 |
| rs3823377 | 6 | 29944253 | A | C | 0.1482 | 0.0196 | 2.99E+07 | 0.0073 | 0.0223 | 0.7431 |
| rs41291790 | 6 | 31572664 | A | G | 0.9494 | 0.0806 | 3.16E+07 | 0.0656 | 0.0814 | 0.4199 |
| rs45593732 | 6 | 31583725 | T | C | 0.4903 | 0.0523 | 3.16E+07 | 0.0368 | 0.0493 | 0.455 |
| rs4676408 | 2 | 241574401 | A | G | 0.1433 | 0.0167 | 2.42E+08 | -0.0023 | 0.0224 | 0.9199 |
| rs4711173 | 6 | 28514583 | C | T | 0.1166 | 0.0201 | 2.85E+07 | 0.0281 | 0.0247 | 0.2557 |
| rs4728142 | 7 | 128573967 | A | G | 0.0995 | 0.0158 | 1.29E+08 | 0.0171 | 0.0219 | 0.4342 |
| rs4993442 | 22 | 30253256 | T | G | -0.0988 | 0.0179 | 3.03E+07 | -0.0394 | 0.0239 | 0.09957 |
| rs56062135 | 15 | 67455630 | T | C | 0.1078 | 0.0184 | 6.75E+07 | -0.0102 | 0.0244 | 0.6766 |
| rs6017342 | 20 | 43065028 | C | A | 0.1944 | 0.017 | 4.31E+07 | -0.0121 | 0.0217 | 0.576901 |
| rs6062496 | 20 | 62329099 | A | G | 0.1359 | 0.0163 | 6.23E+07 | 0.0266 | 0.0225 | 0.2376 |
| rs62180181 | 2 | 199852010 | T | C | 0.1226 | 0.0171 | 2.00E+08 | 0.0038 | 0.0222 | 0.8654 |
| rs6658353 | 1 | 161469054 | C | G | -0.1569 | 0.016 | 1.61E+08 | -0.0184 | 0.0215 | 0.3929 |
| rs67111717 | 5 | 176790162 | G | A | 0.0944 | 0.0171 | 1.77E+08 | 0.0052 | 0.0225 | 0.8173 |
| rs6889364 | 5 | 40347469 | A | G | 0.1318 | 0.0228 | 4.03E+07 | 0.0037 | 0.0273 | 0.8923 |
| rs6915986 | 6 | 32683898 | C | T | 0.404 | 0.0616 | 3.27E+07 | -0.0761 | 0.2174 | 0.7263 |
| rs6933404 | 6 | 137959235 | C | T | 0.1486 | 0.0188 | 1.38E+08 | -0.0146 | 0.0274 | 0.5955 |
| rs7203363 | 16 | 68587692 | A | T | 0.1071 | 0.0189 | 6.86E+07 | 0.022 | 0.028 | 0.4319 |
| rs72704802 | 5 | 554211 | T | C | -0.1223 | 0.0206 | 5.54E+05 | -0.0404 | 0.0325 | 0.2133 |
| rs7523335 | 1 | 8180210 | A | G | -0.1389 | 0.021 | 8.18E+06 | -0.0044 | 0.0243 | 0.8559 |
| rs7544646 | 1 | 2496649 | G | C | -0.1168 | 0.016 | 2.50E+06 | 0.0276 | 0.0216 | 0.201 |
| rs755374 | 5 | 158829294 | T | C | 0.1714 | 0.0171 | 1.59E+08 | -0.0145 | 0.0236 | 0.5373 |
| rs7554511 | 1 | 200877562 | A | C | -0.1448 | 0.0178 | 2.01E+08 | -0.028 | 0.0262 | 0.286 |
| rs7608697 | 2 | 61204641 | C | A | 0.1597 | 0.0161 | 6.12E+07 | 0.0235 | 0.0223 | 0.2911 |
| rs77108272 | 6 | 31455675 | A | C | 0.5479 | 0.062 | 3.15E+07 | 0.1227 | 0.0655 | 0.06103 |
| rs78064630 | 19 | 10562802 | A | G | 0.1759 | 0.0308 | 1.06E+07 | 0.0991 | 0.0458 | 0.03044 |
| rs79051659 | 1 | 151757843 | A | G | 0.1605 | 0.0264 | 1.52E+08 | 0.0015 | 0.0424 | 0.9715 |
| rs7911117 | 10 | 27179596 | G | T | -0.1342 | 0.0239 | 2.72E+07 | -0.0588 | 0.0313 | 0.060439 |
| rs7911680 | 10 | 101293468 | C | A | -0.1525 | 0.0159 | 1.01E+08 | -0.0145 | 0.0215 | 0.5012 |
| rs798506 | 7 | 2788912 | C | T | -0.1206 | 0.0179 | 2.79E+06 | -0.0287 | 0.0228 | 0.2077 |
| rs8073117 | 17 | 70639396 | A | G | -0.1548 | 0.0225 | 7.06E+07 | -0.0068 | 0.0319 | 0.8302 |
| rs872956 | 6 | 33076090 | A | T | -0.1378 | 0.021 | 3.31E+07 | 0.0268 | 0.0267 | 0.3154 |
| rs9271176 | 6 | 32578127 | G | A | -0.3495 | 0.0173 | 3.26E+07 | 0.0214 | 0.024 | 0.3715 |
| rs9611131 | 22 | 39662480 | C | T | -0.1494 | 0.0227 | 3.97E+07 | -0.0271 | 0.0334 | 0.4174 |

UC, Ulcerative colitis; IBS Irritable, bowel syndrome; MR Mendelian randomization

Table S10 Summary information on the SNPs used as genetic instruments for the UC in MR study in gastroesophageal reflux disease

| SNP | chr | pos | A1 | A2 | Exposure (UC) | | | Outcome (GERD) | | |
| --- | --- | --- | --- | --- | --- | --- | --- | --- | --- | --- |
|  |  |  |  |  | β | Se | *P* | β | Se | *P* |
| rs10408351 | 19 | 33754044 | A | G | 0.1548 | 0.0204 | 3.38E+07 | 0.003943 | 0.005838 | 0.499435 |
| rs10737481 | 1 | 20171514 | G | T | 0.2173 | 0.0159 | 2.02E+07 | 0.004099 | 0.004818 | 0.394874 |
| rs10761659 | 10 | 64445564 | G | A | 0.1276 | 0.016 | 6.44E+07 | -0.00582 | 0.004826 | 0.228054 |
| rs10817678 | 9 | 117579457 | A | G | 0.1332 | 0.017 | 1.18E+08 | 0.010777 | 0.005105 | 0.034775 |
| rs11209026 | 1 | 67705958 | A | G | -0.483 | 0.0358 | 6.77E+07 | -0.00283 | 0.009601 | 0.767867 |
| rs1157509 | 5 | 158786110 | G | A | 0.1311 | 0.0217 | 1.59E+08 | -0.00174 | 0.006399 | 0.785315 |
| rs11651246 | 17 | 40759937 | G | T | 0.147 | 0.0219 | 4.08E+07 | -0.00537 | 0.006457 | 0.405355 |
| rs12825700 | 12 | 68492980 | A | G | 0.1889 | 0.0161 | 6.85E+07 | -0.00383 | 0.004939 | 0.438533 |
| rs1317209 | 1 | 20140036 | A | G | 0.1818 | 0.0203 | 2.01E+07 | -0.00832 | 0.006428 | 0.195569 |
| rs1336900 | 1 | 150679033 | A | G | -0.0887 | 0.0163 | 1.51E+08 | -0.00966 | 0.004927 | 0.050001 |
| rs137845 | 22 | 50439430 | G | A | 0.1011 | 0.0158 | 5.04E+07 | 0.004031 | 0.004833 | 0.404341 |
| rs138788 | 22 | 35729721 | A | G | 0.0896 | 0.0162 | 3.57E+07 | 0.001094 | 0.00489 | 0.82293 |
| rs1411262 | 9 | 5459419 | T | C | 0.101 | 0.0179 | 5.46E+06 | 0.000563 | 0.005497 | 0.91844 |
| rs16830407 | 2 | 199625153 | A | G | 0.1078 | 0.0166 | 2.00E+08 | 0.011643 | 0.005063 | 0.021476 |
| rs16940186 | 16 | 86009740 | C | T | 0.1357 | 0.0214 | 8.60E+07 | 0.010532 | 0.006819 | 0.12247 |
| rs1736161 | 21 | 16833222 | A | G | -0.1227 | 0.0161 | 1.68E+07 | -0.00328 | 0.004846 | 0.498198 |
| rs17656349 | 5 | 149605994 | T | C | 0.09 | 0.0159 | 1.50E+08 | -0.00265 | 0.004853 | 0.58533 |
| rs17715902 | 5 | 134451465 | A | G | 0.0974 | 0.0166 | 1.34E+08 | -0.00099 | 0.005125 | 0.847018 |
| rs2045241 | 11 | 114428783 | A | G | -0.1063 | 0.0169 | 1.14E+08 | -0.00109 | 0.005137 | 0.831377 |
| rs2301989 | 7 | 107443871 | A | G | -0.1294 | 0.0161 | 1.07E+08 | -0.00598 | 0.004909 | 0.222835 |
| rs2836881 | 21 | 40466299 | T | G | -0.2217 | 0.0186 | 4.05E+07 | -0.01078 | 0.005463 | 0.048449 |
| rs2838517 | 21 | 45613825 | C | T | -0.1177 | 0.016 | 4.56E+07 | -0.00369 | 0.004959 | 0.456487 |
| rs3024493 | 1 | 206943968 | A | C | 0.21 | 0.0209 | 2.07E+08 | 0.015725 | 0.006644 | 0.01795 |
| rs4728142 | 7 | 128573967 | A | G | 0.0995 | 0.0158 | 1.29E+08 | 0.004012 | 0.004834 | 0.406548 |
| rs4845604 | 1 | 151801680 | A | G | -0.1608 | 0.0239 | 1.52E+08 | -0.00316 | 0.00688 | 0.645946 |
| rs4993442 | 22 | 30253256 | T | G | -0.0988 | 0.0179 | 3.03E+07 | 0.01006 | 0.005579 | 0.071338 |
| rs6017342 | 20 | 43065028 | C | A | 0.1944 | 0.017 | 4.31E+07 | -0.00382 | 0.004829 | 0.428773 |
| rs6933404 | 6 | 137959235 | C | T | 0.1486 | 0.0188 | 1.38E+08 | 0.003704 | 0.005814 | 0.523991 |
| rs7523335 | 1 | 8180210 | A | G | -0.1389 | 0.021 | 8.18E+06 | -0.00663 | 0.006188 | 0.284014 |
| rs7554511 | 1 | 200877562 | A | C | -0.1448 | 0.0178 | 2.01E+08 | 0.007108 | 0.00526 | 0.176598 |
| rs7911117 | 10 | 27179596 | G | T | -0.1342 | 0.0239 | 2.72E+07 | -0.00154 | 0.007095 | 0.827626 |
| rs9611131 | 22 | 39662480 | C | T | -0.1494 | 0.0227 | 3.97E+07 | 0.004951 | 0.006842 | 0.469283 |

UC, Ulcerative colitis; GERD, Gastroesophageal reflux disease; MR, Mendelian randomization

Table S11 Summary information on the SNPs used as genetic instruments for the UC in MR study in Cholelithiasis

| SNP | chr | pos | A1 | A2 | Exposure (UC) | | | Outcome (Cholelithiasis) | | |
| --- | --- | --- | --- | --- | --- | --- | --- | --- | --- | --- |
|  |  |  |  |  | β | Se | *P* | β | Se | *P* |
| rs10272963 | 7 | 107486902 | T | C | -0.1512 | 0.016 | 1.07E+08 | -0.024 | 0.012 | 0.04656 |
| rs10408351 | 19 | 33754044 | A | G | 0.1548 | 0.0204 | 3.38E+07 | -0.011 | 0.0156 | 0.4798 |
| rs10737481 | 1 | 20171514 | G | T | 0.2173 | 0.0159 | 2.02E+07 | 0.0108 | 0.012 | 0.3672 |
| rs10761659 | 10 | 64445564 | G | A | 0.1276 | 0.016 | 6.44E+07 | 0.0217 | 0.012 | 0.06986 |
| rs10817678 | 9 | 117579457 | A | G | 0.1332 | 0.017 | 1.18E+08 | 0.0074 | 0.0129 | 0.565999 |
| rs11209026 | 1 | 67705958 | A | G | -0.483 | 0.0358 | 6.77E+07 | -0.0118 | 0.0289 | 0.6838 |
| rs1131095 | 3 | 49714225 | C | T | 0.1593 | 0.0168 | 4.97E+07 | -0.0077 | 0.0122 | 0.5287 |
| rs113986290 | 6 | 19781009 | T | C | -0.3066 | 0.0531 | 1.98E+07 | 0.0436 | 0.0446 | 0.3281 |
| rs1157509 | 5 | 158786110 | G | A | 0.1311 | 0.0217 | 1.59E+08 | -0.0006 | 0.0165 | 0.9715 |
| rs11645239 | 16 | 23847062 | G | C | -0.1174 | 0.02 | 2.38E+07 | 0.0117 | 0.0163 | 0.4722 |
| rs11651246 | 17 | 40759937 | G | T | 0.147 | 0.0219 | 4.08E+07 | 0.0188 | 0.0154 | 0.2214 |
| rs116724447 | 5 | 158581640 | A | G | -0.339 | 0.0572 | 1.59E+08 | -0.036 | 0.0353 | 0.3082 |
| rs117292830 | 6 | 31218268 | A | G | 0.4948 | 0.0495 | 3.12E+07 | 0.0167 | 0.0316 | 0.598199 |
| rs1265098 | 6 | 31106177 | C | T | -0.1646 | 0.0203 | 3.11E+07 | 0.0054 | 0.0137 | 0.6948 |
| rs12825700 | 12 | 68492980 | A | G | 0.1889 | 0.0161 | 6.85E+07 | 0.0103 | 0.0128 | 0.4206 |
| rs12936409 | 17 | 38043649 | T | C | 0.1365 | 0.0158 | 3.80E+07 | -0.0413 | 0.012 | 0.000604 |
| rs1317209 | 1 | 20140036 | A | G | 0.1818 | 0.0203 | 2.01E+07 | 0.0026 | 0.0144 | 0.8552 |
| rs13200059 | 6 | 111943234 | A | G | 0.2944 | 0.0436 | 1.12E+08 | -0.037 | 0.0281 | 0.1879 |
| rs1336900 | 1 | 150679033 | A | G | -0.0887 | 0.0163 | 1.51E+08 | -0.0196 | 0.0122 | 0.1078 |
| rs1359946 | 13 | 27536972 | A | G | 0.1571 | 0.0202 | 2.75E+07 | 0.007 | 0.0157 | 0.6541 |
| rs137845 | 22 | 50439430 | G | A | 0.1011 | 0.0158 | 5.04E+07 | -0.0069 | 0.0121 | 0.5695 |
| rs138788 | 22 | 35729721 | A | G | 0.0896 | 0.0162 | 3.57E+07 | 0.0133 | 0.0121 | 0.2718 |
| rs1411262 | 9 | 5459419 | T | C | 0.101 | 0.0179 | 5.46E+06 | -0.0069 | 0.0135 | 0.6087 |
| rs141725002 | 6 | 30971976 | A | G | 0.4885 | 0.0883 | 3.10E+07 | -0.1976 | 0.1797 | 0.2715 |
| rs16830407 | 2 | 199625153 | A | G | 0.1078 | 0.0166 | 2.00E+08 | 0.0002 | 0.0122 | 0.9886 |
| rs17190351 | 6 | 31047448 | A | G | 0.4301 | 0.0561 | 3.10E+07 | 0.0089 | 0.0583 | 0.8792 |
| rs17202899 | 6 | 32434481 | C | T | 0.2211 | 0.0312 | 3.24E+07 | -0.0021 | 0.025 | 0.9317 |
| rs1736161 | 21 | 16833222 | A | G | -0.1227 | 0.0161 | 1.68E+07 | -0.0037 | 0.0122 | 0.7596 |
| rs17656349 | 5 | 149605994 | T | C | 0.09 | 0.0159 | 1.50E+08 | 0.0239 | 0.0124 | 0.05309 |
| rs17715902 | 5 | 134451465 | A | G | 0.0974 | 0.0166 | 1.34E+08 | 0.0208 | 0.0126 | 0.098431 |
| rs1811711 | 2 | 228670476 | G | C | -0.1299 | 0.0223 | 2.29E+08 | 0.0025 | 0.017 | 0.8819 |
| rs1846190 | 6 | 32583813 | A | G | -0.2267 | 0.019 | 3.26E+07 | -0.0124 | 0.0136 | 0.3607 |
| rs1887428 | 9 | 4984530 | C | G | -0.167 | 0.0166 | 4.98E+06 | -0.0109 | 0.0124 | 0.3772 |
| rs2045241 | 11 | 114428783 | A | G | -0.1063 | 0.0169 | 1.14E+08 | 0.0017 | 0.0123 | 0.8885 |
| rs2212434 | 11 | 76281593 | T | C | 0.1252 | 0.0159 | 7.63E+07 | -0.0016 | 0.0122 | 0.8937 |
| rs2294633 | 1 | 20090235 | T | C | -0.0985 | 0.0177 | 2.01E+07 | -0.0081 | 0.0126 | 0.522701 |
| rs2301989 | 7 | 107443871 | A | G | -0.1294 | 0.0161 | 1.07E+08 | -0.0228 | 0.012 | 0.057439 |
| rs2816980 | 1 | 200078035 | T | G | 0.1941 | 0.026 | 2.00E+08 | 0.0206 | 0.0197 | 0.2957 |
| rs2836881 | 21 | 40466299 | T | G | -0.2217 | 0.0186 | 4.05E+07 | -0.0016 | 0.0139 | 0.9058 |
| rs3024493 | 1 | 206943968 | A | C | 0.21 | 0.0209 | 2.07E+08 | -0.027 | 0.0166 | 0.103 |
| rs3097666 | 6 | 33022788 | C | G | -0.2154 | 0.0304 | 3.30E+07 | 0.0095 | 0.024 | 0.691 |
| rs3129299 | 6 | 32900787 | T | C | -0.14 | 0.0236 | 3.29E+07 | -0.0235 | 0.0207 | 0.2565 |
| rs3130500 | 6 | 31119976 | A | T | -0.1184 | 0.0183 | 3.11E+07 | 0.0139 | 0.0139 | 0.3203 |
| rs34638686 | 3 | 48682658 | T | C | 0.1795 | 0.0262 | 4.87E+07 | -0.0131 | 0.0182 | 0.4717 |
| rs34920465 | 1 | 22700351 | G | A | -0.1708 | 0.0213 | 2.27E+07 | 0.0033 | 0.0209 | 0.8751 |
| rs3812565 | 9 | 139272502 | C | T | 0.1335 | 0.016 | 1.39E+08 | -0.0048 | 0.0124 | 0.697199 |
| rs3820328 | 1 | 20142447 | G | A | -0.1662 | 0.0164 | 2.01E+07 | 0.0076 | 0.0122 | 0.5315 |
| rs3823377 | 6 | 29944253 | A | C | 0.1482 | 0.0196 | 2.99E+07 | 0.0318 | 0.0124 | 0.0106 |
| rs41291790 | 6 | 31572664 | A | G | 0.9494 | 0.0806 | 3.16E+07 | -0.0008 | 0.0451 | 0.9854 |
| rs45593732 | 6 | 31583725 | T | C | 0.4903 | 0.0523 | 3.16E+07 | 0.0231 | 0.0274 | 0.3993 |
| rs4676408 | 2 | 241574401 | A | G | 0.1433 | 0.0167 | 2.42E+08 | 0.0242 | 0.0125 | 0.05288 |
| rs4711173 | 6 | 28514583 | C | T | 0.1166 | 0.0201 | 2.85E+07 | 0.0121 | 0.0137 | 0.3786 |
| rs4728142 | 7 | 128573967 | A | G | 0.0995 | 0.0158 | 1.29E+08 | -0.0105 | 0.0122 | 0.3868 |
| rs4993442 | 22 | 30253256 | T | G | -0.0988 | 0.0179 | 3.03E+07 | -0.0011 | 0.0133 | 0.9326 |
| rs56062135 | 15 | 67455630 | T | C | 0.1078 | 0.0184 | 6.75E+07 | 0.0058 | 0.0136 | 0.672 |
| rs6017342 | 20 | 43065028 | C | A | 0.1944 | 0.017 | 4.31E+07 | -0.0487 | 0.0121 | 5.93E-05 |
| rs6062496 | 20 | 62329099 | A | G | 0.1359 | 0.0163 | 6.23E+07 | -0.0246 | 0.0125 | 0.049741 |
| rs62180181 | 2 | 199852010 | T | C | 0.1226 | 0.0171 | 2.00E+08 | 0.0281 | 0.0124 | 0.02282 |
| rs6658353 | 1 | 161469054 | C | G | -0.1569 | 0.016 | 1.61E+08 | -0.004 | 0.012 | 0.7391 |
| rs67111717 | 5 | 176790162 | G | A | 0.0944 | 0.0171 | 1.77E+08 | 0.0131 | 0.0125 | 0.2942 |
| rs6889364 | 5 | 40347469 | A | G | 0.1318 | 0.0228 | 4.03E+07 | 0.0006 | 0.0152 | 0.9662 |
| rs6915986 | 6 | 32683898 | C | T | 0.404 | 0.0616 | 3.27E+07 | 0.0582 | 0.1195 | 0.626299 |
| rs6933404 | 6 | 137959235 | C | T | 0.1486 | 0.0188 | 1.38E+08 | 0.0308 | 0.0153 | 0.04377 |
| rs7203363 | 16 | 68587692 | A | T | 0.1071 | 0.0189 | 6.86E+07 | 0.0097 | 0.0156 | 0.5358 |
| rs72704802 | 5 | 554211 | T | C | -0.1223 | 0.0206 | 5.54E+05 | -0.0148 | 0.0181 | 0.412 |
| rs7523335 | 1 | 8180210 | A | G | -0.1389 | 0.021 | 8.18E+06 | 0.0096 | 0.0135 | 0.4771 |
| rs7544646 | 1 | 2496649 | G | C | -0.1168 | 0.016 | 2.50E+06 | -0.0005 | 0.012 | 0.9687 |
| rs755374 | 5 | 158829294 | T | C | 0.1714 | 0.0171 | 1.59E+08 | 0.0112 | 0.0131 | 0.3923 |
| rs7554511 | 1 | 200877562 | A | C | -0.1448 | 0.0178 | 2.01E+08 | -0.0207 | 0.0146 | 0.1561 |
| rs7608697 | 2 | 61204641 | C | A | 0.1597 | 0.0161 | 6.12E+07 | -0.0149 | 0.0124 | 0.2292 |
| rs77108272 | 6 | 31455675 | A | C | 0.5479 | 0.062 | 3.15E+07 | -0.0485 | 0.0367 | 0.1864 |
| rs78064630 | 19 | 10562802 | A | G | 0.1759 | 0.0308 | 1.06E+07 | 0.0097 | 0.0255 | 0.704399 |
| rs79051659 | 1 | 151757843 | A | G | 0.1605 | 0.0264 | 1.52E+08 | -0.0042 | 0.0235 | 0.8568 |
| rs7911117 | 10 | 27179596 | G | T | -0.1342 | 0.0239 | 2.72E+07 | 0.0063 | 0.0174 | 0.7193 |
| rs7911680 | 10 | 101293468 | C | A | -0.1525 | 0.0159 | 1.01E+08 | 0.0078 | 0.012 | 0.5156 |
| rs798506 | 7 | 2788912 | C | T | -0.1206 | 0.0179 | 2.79E+06 | -0.0208 | 0.0127 | 0.1002 |
| rs8073117 | 17 | 70639396 | A | G | -0.1548 | 0.0225 | 7.06E+07 | 0.0085 | 0.0177 | 0.631499 |
| rs872956 | 6 | 33076090 | A | T | -0.1378 | 0.021 | 3.31E+07 | 0.0076 | 0.0149 | 0.6097 |
| rs9271176 | 6 | 32578127 | G | A | -0.3495 | 0.0173 | 3.26E+07 | 0.0145 | 0.0133 | 0.2754 |
| rs9611131 | 22 | 39662480 | C | T | -0.1494 | 0.0227 | 3.97E+07 | 0.0054 | 0.0186 | 0.770999 |

UC, Ulcerative colitis; MR, Mendelian randomization

Table S12 Summary information on the SNPs used as genetic instruments for the UC in MR study in Interleukin-6

| SNP | chr | pos | A1 | A2 | Exposure (UC) | | | Outcome (IL-6) | | |
| --- | --- | --- | --- | --- | --- | --- | --- | --- | --- | --- |
|  |  |  |  |  | β | Se | *P* | β | Se | *P* |
| rs10272963 | 1 | 107486902 | T | C | -0.1512 | 0.016 | 1.07E+08 | -0.0068 | 0.0119 | 0.5671 |
| rs10408351 | 2 | 33754044 | A | G | 0.1548 | 0.0204 | 3.38E+07 | 0.013 | 0.0162 | 0.4202 |
| rs10737481 | 3 | 20171514 | G | T | 0.2173 | 0.0159 | 2.02E+07 | -0.0018 | 0.0118 | 0.878 |
| rs10761659 | 4 | 64445564 | G | A | 0.1276 | 0.016 | 6.44E+07 | -0.0102 | 0.0117 | 0.3862 |
| rs10817678 | 5 | 117579457 | A | G | 0.1332 | 0.017 | 1.18E+08 | 0.0125 | 0.0125 | 0.32 |
| rs1131095 | 6 | 49714225 | C | T | 0.1593 | 0.0168 | 4.97E+07 | 0.0034 | 0.0131 | 0.7973 |
| rs113986290 | 7 | 19781009 | T | C | -0.3066 | 0.0531 | 1.98E+07 | 0.0427 | 0.0437 | 0.3284 |
| rs1157509 | 8 | 158786110 | G | A | 0.1311 | 0.0217 | 1.59E+08 | 0.0412 | 0.016 | 0.009849 |
| rs11645239 | 9 | 23847062 | G | C | -0.1174 | 0.02 | 2.38E+07 | -0.0112 | 0.0157 | 0.4768 |
| rs11651246 | 10 | 40759937 | G | T | 0.147 | 0.0219 | 4.08E+07 | 0.0079 | 0.0162 | 0.6257 |
| rs116724447 | 11 | 158581640 | A | G | -0.339 | 0.0572 | 1.59E+08 | -0.0271 | 0.0382 | 0.4782 |
| rs117292830 | 12 | 31218268 | A | G | 0.4948 | 0.0495 | 3.12E+07 | 0.0221 | 0.0366 | 0.546799 |
| rs1265098 | 13 | 31106177 | C | T | -0.1646 | 0.0203 | 3.11E+07 | 0.0055 | 0.0145 | 0.7061 |
| rs12825700 | 14 | 68492980 | A | G | 0.1889 | 0.0161 | 6.85E+07 | 0.0072 | 0.0122 | 0.5581 |
| rs12936409 | 15 | 38043649 | T | C | 0.1365 | 0.0158 | 3.80E+07 | 0.0086 | 0.0118 | 0.4622 |
| rs1317209 | 16 | 20140036 | A | G | 0.1818 | 0.0203 | 2.01E+07 | 0.0034 | 0.0165 | 0.8377 |
| rs13200059 | 17 | 111943234 | A | G | 0.2944 | 0.0436 | 1.12E+08 | 0.0408 | 0.0331 | 0.2174 |
| rs1336900 | 18 | 150679033 | A | G | -0.0887 | 0.0163 | 1.51E+08 | 0.005 | 0.012 | 0.6754 |
| rs1359946 | 19 | 27536972 | A | G | 0.1571 | 0.0202 | 2.75E+07 | 0.0079 | 0.0152 | 0.604301 |
| rs137845 | 20 | 50439430 | G | A | 0.1011 | 0.0158 | 5.04E+07 | 0.0037 | 0.012 | 0.757 |
| rs138788 | 21 | 35729721 | A | G | 0.0896 | 0.0162 | 3.57E+07 | 0.0149 | 0.0134 | 0.2689 |
| rs1411262 | 22 | 5459419 | T | C | 0.101 | 0.0179 | 5.46E+06 | -0.0198 | 0.014 | 0.1591 |
| rs141725002 | 23 | 30971976 | A | G | 0.4885 | 0.0883 | 3.10E+07 | -0.0226 | 0.0764 | 0.7673 |
| rs16830407 | 24 | 199625153 | A | G | 0.1078 | 0.0166 | 2.00E+08 | -0.0046 | 0.0123 | 0.7057 |
| rs16940186 | 25 | 86009740 | C | T | 0.1357 | 0.0214 | 8.60E+07 | 0.0055 | 0.0164 | 0.7402 |
| rs17190351 | 26 | 31047448 | A | G | 0.4301 | 0.0561 | 3.10E+07 | 0.0259 | 0.0431 | 0.5483 |
| rs17202899 | 27 | 32434481 | C | T | 0.2211 | 0.0312 | 3.24E+07 | -0.036 | 0.0219 | 0.1004 |
| rs1736161 | 28 | 16833222 | A | G | -0.1227 | 0.0161 | 1.68E+07 | 0.012 | 0.0123 | 0.3291 |
| rs17656349 | 29 | 149605994 | T | C | 0.09 | 0.0159 | 1.50E+08 | 0.001 | 0.012 | 0.9325 |
| rs17715902 | 30 | 134451465 | A | G | 0.0974 | 0.0166 | 1.34E+08 | 0.0047 | 0.0124 | 0.7052 |
| rs1811711 | 31 | 228670476 | G | C | -0.1299 | 0.0223 | 2.29E+08 | -0.0053 | 0.018 | 0.7662 |
| rs1846190 | 32 | 32583813 | A | G | -0.2267 | 0.019 | 3.26E+07 | 0.0558 | 0.0148 | 0.000153 |
| rs1887428 | 33 | 4984530 | C | G | -0.167 | 0.0166 | 4.98E+06 | -0.0278 | 0.0127 | 0.02792 |
| rs2045241 | 34 | 114428783 | A | G | -0.1063 | 0.0169 | 1.14E+08 | 0.0205 | 0.0123 | 0.094809 |
| rs2212434 | 35 | 76281593 | T | C | 0.1252 | 0.0159 | 7.63E+07 | 0.0031 | 0.0119 | 0.7948 |
| rs2294633 | 36 | 20090235 | T | C | -0.0985 | 0.0177 | 2.01E+07 | 0.0026 | 0.0155 | 0.8648 |
| rs2301989 | 37 | 107443871 | A | G | -0.1294 | 0.0161 | 1.07E+08 | 0.0093 | 0.0125 | 0.4561 |
| rs2816980 | 38 | 200078035 | T | G | 0.1941 | 0.026 | 2.00E+08 | 0.0139 | 0.0194 | 0.4738 |
| rs2836881 | 39 | 40466299 | T | G | -0.2217 | 0.0186 | 4.05E+07 | -0.0506 | 0.0138 | 0.000241 |
| rs2838517 | 40 | 45613825 | C | T | -0.1177 | 0.016 | 4.56E+07 | 0.0083 | 0.012 | 0.4854 |
| rs3024493 | 41 | 206943968 | A | C | 0.21 | 0.0209 | 2.07E+08 | 0.0391 | 0.0164 | 0.01671 |
| rs3097666 | 42 | 33022788 | C | G | -0.2154 | 0.0304 | 3.30E+07 | -0.0501 | 0.0279 | 0.072719 |
| rs3129299 | 43 | 32900787 | T | C | -0.14 | 0.0236 | 3.29E+07 | 0.0032 | 0.0173 | 0.8528 |
| rs3130500 | 44 | 31119976 | A | T | -0.1184 | 0.0183 | 3.11E+07 | -0.0057 | 0.0139 | 0.680899 |
| rs34638686 | 45 | 48682658 | T | C | 0.1795 | 0.0262 | 4.87E+07 | 0.014 | 0.0194 | 0.4705 |
| rs34920465 | 46 | 22700351 | G | A | -0.1708 | 0.0213 | 2.27E+07 | -0.0343 | 0.0165 | 0.03735 |
| rs3812565 | 47 | 139272502 | C | T | 0.1335 | 0.016 | 1.39E+08 | -0.0036 | 0.0137 | 0.793201 |
| rs3820328 | 48 | 20142447 | G | A | -0.1662 | 0.0164 | 2.01E+07 | 0.002 | 0.0122 | 0.8667 |
| rs3823377 | 49 | 29944253 | A | C | 0.1482 | 0.0196 | 2.99E+07 | 0.0271 | 0.0123 | 0.02731 |
| rs41291790 | 50 | 31572664 | A | G | 0.9494 | 0.0806 | 3.16E+07 | 0.0158 | 0.1038 | 0.8786 |
| rs45593732 | 51 | 31583725 | T | C | 0.4903 | 0.0523 | 3.16E+07 | 0.0324 | 0.0436 | 0.4568 |
| rs4676408 | 52 | 241574401 | A | G | 0.1433 | 0.0167 | 2.42E+08 | 0.0099 | 0.0123 | 0.4178 |
| rs4711173 | 53 | 28514583 | C | T | 0.1166 | 0.0201 | 2.85E+07 | 0.0133 | 0.0154 | 0.3885 |
| rs4728142 | 54 | 128573967 | A | G | 0.0995 | 0.0158 | 1.29E+08 | 0.0271 | 0.0133 | 0.042211 |
| rs4845604 | 55 | 151801680 | A | G | -0.1608 | 0.0239 | 1.52E+08 | 0.0053 | 0.0174 | 0.760801 |
| rs4993442 | 56 | 30253256 | T | G | -0.0988 | 0.0179 | 3.03E+07 | 0.0106 | 0.0132 | 0.4234 |
| rs56062135 | 57 | 67455630 | T | C | 0.1078 | 0.0184 | 6.75E+07 | 0.0183 | 0.0139 | 0.1899 |
| rs6017342 | 58 | 43065028 | C | A | 0.1944 | 0.017 | 4.31E+07 | -0.0078 | 0.012 | 0.5147 |
| rs6062496 | 59 | 62329099 | A | G | 0.1359 | 0.0163 | 6.23E+07 | -0.001 | 0.012 | 0.9359 |
| rs62180181 | 60 | 199852010 | T | C | 0.1226 | 0.0171 | 2.00E+08 | -0.0199 | 0.0133 | 0.1335 |
| rs6658353 | 61 | 161469054 | C | G | -0.1569 | 0.016 | 1.61E+08 | -0.008 | 0.0118 | 0.4971 |
| rs67111717 | 62 | 176790162 | G | A | 0.0944 | 0.0171 | 1.77E+08 | -0.0064 | 0.0128 | 0.619699 |
| rs6889364 | 63 | 40347469 | A | G | 0.1318 | 0.0228 | 4.03E+07 | 0.0211 | 0.0169 | 0.2119 |
| rs6915986 | 64 | 32683898 | C | T | 0.404 | 0.0616 | 3.27E+07 | -0.0201 | 0.0595 | 0.734999 |
| rs6933404 | 65 | 137959235 | C | T | 0.1486 | 0.0188 | 1.38E+08 | 0.0108 | 0.0153 | 0.4805 |
| rs7203363 | 66 | 68587692 | A | T | 0.1071 | 0.0189 | 6.86E+07 | 0.0091 | 0.0153 | 0.554501 |
| rs72704802 | 67 | 554211 | T | C | -0.1223 | 0.0206 | 5.54E+05 | -0.0041 | 0.0163 | 0.7991 |
| rs7523335 | 68 | 8180210 | A | G | -0.1389 | 0.021 | 8.18E+06 | 0.0005 | 0.0161 | 0.9776 |
| rs7544646 | 69 | 2496649 | G | C | -0.1168 | 0.016 | 2.50E+06 | 0.0023 | 0.0127 | 0.8558 |
| rs755374 | 70 | 158829294 | T | C | 0.1714 | 0.0171 | 1.59E+08 | 0.0122 | 0.0127 | 0.3378 |
| rs7554511 | 71 | 200877562 | A | C | -0.1448 | 0.0178 | 2.01E+08 | -0.021 | 0.0135 | 0.1185 |
| rs7608697 | 72 | 61204641 | C | A | 0.1597 | 0.0161 | 6.12E+07 | 0.0147 | 0.0121 | 0.225 |
| rs77108272 | 73 | 31455675 | A | C | 0.5479 | 0.062 | 3.15E+07 | 0.0102 | 0.0525 | 0.8454 |
| rs78064630 | 74 | 10562802 | A | G | 0.1759 | 0.0308 | 1.06E+07 | 0.0178 | 0.0235 | 0.4483 |
| rs79051659 | 75 | 151757843 | A | G | 0.1605 | 0.0264 | 1.52E+08 | 0.025 | 0.0219 | 0.2543 |
| rs7911117 | 76 | 27179596 | G | T | -0.1342 | 0.0239 | 2.72E+07 | 0.0409 | 0.0197 | 0.03744 |
| rs7911680 | 77 | 101293468 | C | A | -0.1525 | 0.0159 | 1.01E+08 | -0.0041 | 0.012 | 0.731601 |
| rs798506 | 78 | 2788912 | C | T | -0.1206 | 0.0179 | 2.79E+06 | -0.0037 | 0.014 | 0.7922 |
| rs8073117 | 79 | 70639396 | A | G | -0.1548 | 0.0225 | 7.06E+07 | 0.0114 | 0.0163 | 0.4826 |
| rs872956 | 80 | 33076090 | A | T | -0.1378 | 0.021 | 3.31E+07 | -0.006 | 0.0163 | 0.7121 |

UC, Ulcerative colitis; IL-6, Interleukin 6; MR, Mendelian randomization

Table S13 Summary information on the SNPs used as genetic instruments for the UC in MR study in c-reactive protein

| SNP | chr | pos | A1 | A2 | Exposure (UC) | | | Outcome (CRP) | | |
| --- | --- | --- | --- | --- | --- | --- | --- | --- | --- | --- |
|  |  |  |  |  | β | Se | *P* | β | Se | *P* |
| rs10408351 | 19 | 33754044 | A | G | 0.1548 | 0.0204 | 3.38E+07 | -0.0061 | 0.00556 | 0.272999 |
| rs10737481 | 1 | 20171514 | G | T | 0.2173 | 0.0159 | 2.02E+07 | 0.001737 | 0.003569 | 0.626457 |
| rs10761659 | 10 | 64445564 | G | A | 0.1276 | 0.016 | 6.44E+07 | -0.00774 | 0.003837 | 0.043818 |
| rs10817678 | 9 | 117579457 | A | G | 0.1332 | 0.017 | 1.18E+08 | -0.00586 | 0.003946 | 0.137634 |
| rs11209026 | 1 | 67705958 | A | G | -0.483 | 0.0358 | 6.77E+07 | -0.00209 | 0.007529 | 0.781644 |
| rs1157509 | 5 | 158786110 | G | A | 0.1311 | 0.0217 | 1.59E+08 | 0.010274 | 0.004852 | 0.034221 |
| rs11651246 | 17 | 40759937 | G | T | 0.147 | 0.0219 | 4.08E+07 | -0.00461 | 0.005376 | 0.391086 |
| rs1265098 | 6 | 31106177 | C | T | -0.1646 | 0.0203 | 3.11E+07 | 0.018403 | 0.004679 | 8.45E-05 |
| rs12825700 | 12 | 68492980 | A | G | 0.1889 | 0.0161 | 6.85E+07 | 0.002827 | 0.003711 | 0.446215 |
| rs1317209 | 1 | 20140036 | A | G | 0.1818 | 0.0203 | 2.01E+07 | 0.012671 | 0.004914 | 0.009933 |
| rs137845 | 22 | 50439430 | G | A | 0.1011 | 0.0158 | 5.04E+07 | -0.00602 | 0.003619 | 0.096514 |
| rs138788 | 22 | 35729721 | A | G | 0.0896 | 0.0162 | 3.57E+07 | 0.001869 | 0.003705 | 0.614031 |
| rs1411262 | 9 | 5459419 | T | C | 0.101 | 0.0179 | 5.46E+06 | 0.002572 | 0.004102 | 0.530708 |
| rs16830407 | 2 | 199625153 | A | G | 0.1078 | 0.0166 | 2.00E+08 | -0.00151 | 0.003795 | 0.690427 |
| rs16940186 | 16 | 86009740 | C | T | 0.1357 | 0.0214 | 8.60E+07 | -0.00416 | 0.005592 | 0.457237 |
| rs1736161 | 21 | 16833222 | A | G | -0.1227 | 0.0161 | 1.68E+07 | -0.00267 | 0.003733 | 0.47384 |
| rs17656349 | 5 | 149605994 | T | C | 0.09 | 0.0159 | 1.50E+08 | 0.004608 | 0.00357 | 0.196744 |
| rs17715902 | 5 | 134451465 | A | G | 0.0974 | 0.0166 | 1.34E+08 | 0.005769 | 0.003968 | 0.145953 |
| rs2045241 | 11 | 114428783 | A | G | -0.1063 | 0.0169 | 1.14E+08 | 0.0007 | 0.003727 | 0.850922 |
| rs2301989 | 7 | 107443871 | A | G | -0.1294 | 0.0161 | 1.07E+08 | 0.003257 | 0.003922 | 0.406218 |
| rs2836881 | 21 | 40466299 | T | G | -0.2217 | 0.0186 | 4.05E+07 | -0.04074 | 0.004287 | 2.17E-21 |
| rs2838517 | 21 | 45613825 | C | T | -0.1177 | 0.016 | 4.56E+07 | -0.00211 | 0.003735 | 0.571498 |
| rs3024493 | 1 | 206943968 | A | C | 0.21 | 0.0209 | 2.07E+08 | 0.012563 | 0.00504 | 0.012698 |
| rs3129299 | 6 | 32900787 | T | C | -0.14 | 0.0236 | 3.29E+07 | 0.002519 | 0.005485 | 0.646045 |
| rs4711173 | 6 | 28514583 | C | T | 0.1166 | 0.0201 | 2.85E+07 | 0.001326 | 0.004376 | 0.761835 |
| rs4728142 | 7 | 128573967 | A | G | 0.0995 | 0.0158 | 1.29E+08 | 0.006855 | 0.00382 | 0.072713 |
| rs4845604 | 1 | 151801680 | A | G | -0.1608 | 0.0239 | 1.52E+08 | -0.00176 | 0.005486 | 0.747877 |
| rs4993442 | 22 | 30253256 | T | G | -0.0988 | 0.0179 | 3.03E+07 | -0.00762 | 0.004007 | 0.057338 |
| rs6017342 | 20 | 43065028 | C | A | 0.1944 | 0.017 | 4.31E+07 | 0.01232 | 0.005393 | 0.022354 |
| rs6933404 | 6 | 137959235 | C | T | 0.1486 | 0.0188 | 1.38E+08 | 0.021476 | 0.004443 | 1.37E-06 |
| rs7523335 | 1 | 8180210 | A | G | -0.1389 | 0.021 | 8.18E+06 | -0.01321 | 0.004741 | 0.005362 |
| rs7554511 | 1 | 200877562 | A | C | -0.1448 | 0.0178 | 2.01E+08 | -0.01069 | 0.00433 | 0.013587 |
| rs7911117 | 10 | 27179596 | G | T | -0.1342 | 0.0239 | 2.72E+07 | 0.004019 | 0.00544 | 0.459964 |
| rs872956 | 6 | 33076090 | A | T | -0.1378 | 0.021 | 3.31E+07 | 0.003236 | 0.00436 | 0.457923 |
| rs9611131 | 22 | 39662480 | C | T | -0.1494 | 0.0227 | 3.97E+07 | 0.001916 | 0.00536 | 0.720719 |

UC, Ulcerative colitis; CRP, C-reactive protein; MR, Mendelian randomization

Table S14 Summary information on the SNPs used as genetic instruments for the UC in MR study in Tumor necrosis factor-α

| SNP | chr | pos | A1 | A2 | Exposure (UC) | | | Outcome(TNF-α) | | |
| --- | --- | --- | --- | --- | --- | --- | --- | --- | --- | --- |
|  |  |  |  |  | β | Se | *P* | β | Se | *P* |
| rs10272963 | 7 | 107486902 | T | C | -0.1512 | 0.016 | 1.07E+08 | -0.0029 | 0.0245 | 0.9087 |
| rs10408351 | 19 | 33754044 | A | G | 0.1548 | 0.0204 | 3.38E+07 | 0.0133 | 0.0328 | 0.6988 |
| rs10737481 | 1 | 20171514 | G | T | 0.2173 | 0.0159 | 2.02E+07 | 0.002 | 0.0242 | 0.9347 |
| rs10761659 | 10 | 64445564 | G | A | 0.1276 | 0.016 | 6.44E+07 | -0.0225 | 0.0246 | 0.3559 |
| rs10817678 | 9 | 117579457 | A | G | 0.1332 | 0.017 | 1.18E+08 | 0.004 | 0.026 | 0.8791 |
| rs11209026 | 1 | 67705958 | A | G | -0.483 | 0.0358 | 6.77E+07 | 0.0197 | 0.0559 | 0.7602 |
| rs1131095 | 3 | 49714225 | C | T | 0.1593 | 0.0168 | 4.97E+07 | 0.0018 | 0.0242 | 0.9492 |
| rs113986290 | 6 | 19781009 | T | C | -0.3066 | 0.0531 | 1.98E+07 | -0.0538 | 0.0992 | 0.637699 |
| rs1157509 | 5 | 158786110 | G | A | 0.1311 | 0.0217 | 1.59E+08 | -0.017 | 0.0335 | 0.610401 |
| rs11645239 | 16 | 23847062 | G | C | -0.1174 | 0.02 | 2.38E+07 | 0.0417 | 0.0376 | 0.2684 |
| rs11651246 | 17 | 40759937 | G | T | 0.147 | 0.0219 | 4.08E+07 | -0.0087 | 0.0313 | 0.7828 |
| rs116724447 | 5 | 158581640 | A | G | -0.339 | 0.0572 | 1.59E+08 | -0.001 | 0.0702 | 0.9694 |
| rs117292830 | 6 | 31218268 | A | G | 0.4948 | 0.0495 | 3.12E+07 | 0.0086 | 0.0637 | 0.9031 |
| rs1265098 | 6 | 31106177 | C | T | -0.1646 | 0.0203 | 3.11E+07 | 0.0209 | 0.0253 | 0.4105 |
| rs12825700 | 12 | 68492980 | A | G | 0.1889 | 0.0161 | 6.85E+07 | -0.0004 | 0.0257 | 0.9912 |
| rs12936409 | 17 | 38043649 | T | C | 0.1365 | 0.0158 | 3.80E+07 | -0.0021 | 0.0241 | 0.9247 |
| rs1317209 | 1 | 20140036 | A | G | 0.1818 | 0.0203 | 2.01E+07 | -0.0089 | 0.029 | 0.7519 |
| rs13200059 | 6 | 111943234 | A | G | 0.2944 | 0.0436 | 1.12E+08 | -0.0252 | 0.0543 | 0.6624 |
| rs1336900 | 1 | 150679033 | A | G | -0.0887 | 0.0163 | 1.51E+08 | -0.0448 | 0.0247 | 0.06995 |
| rs1359946 | 13 | 27536972 | A | G | 0.1571 | 0.0202 | 2.75E+07 | 0.0023 | 0.0318 | 0.9614 |
| rs137845 | 22 | 50439430 | G | A | 0.1011 | 0.0158 | 5.04E+07 | -0.0716 | 0.0244 | 0.003475 |
| rs138788 | 22 | 35729721 | A | G | 0.0896 | 0.0162 | 3.57E+07 | 0.0193 | 0.0242 | 0.4246 |
| rs1411262 | 9 | 5459419 | T | C | 0.101 | 0.0179 | 5.46E+06 | 0.062 | 0.0275 | 0.02391 |
| rs16830407 | 2 | 199625153 | A | G | 0.1078 | 0.0166 | 2.00E+08 | -0.0589 | 0.0241 | 0.01449 |
| rs16940186 | 16 | 86009740 | C | T | 0.1357 | 0.0214 | 8.60E+07 | -0.0013 | 0.0354 | 0.9469 |
| rs17202899 | 6 | 32434481 | C | T | 0.2211 | 0.0312 | 3.24E+07 | -0.1171 | 0.052 | 0.02411 |
| rs1736161 | 21 | 16833222 | A | G | -0.1227 | 0.0161 | 1.68E+07 | 0.0015 | 0.0247 | 0.9545 |
| rs17656349 | 5 | 149605994 | T | C | 0.09 | 0.0159 | 1.50E+08 | 0.0162 | 0.0249 | 0.5078 |
| rs17715902 | 5 | 134451465 | A | G | 0.0974 | 0.0166 | 1.34E+08 | 0.0065 | 0.0256 | 0.8009 |
| rs1811711 | 2 | 228670476 | G | C | -0.1299 | 0.0223 | 2.29E+08 | -0.0145 | 0.0361 | 0.6949 |
| rs1846190 | 6 | 32583813 | A | G | -0.2267 | 0.019 | 3.26E+07 | 0.019 | 0.027 | 0.4815 |
| rs1887428 | 9 | 4984530 | C | G | -0.167 | 0.0166 | 4.98E+06 | 0.0098 | 0.0258 | 0.7039 |
| rs2045241 | 11 | 114428783 | A | G | -0.1063 | 0.0169 | 1.14E+08 | 0.0171 | 0.0248 | 0.482 |
| rs2212434 | 11 | 76281593 | T | C | 0.1252 | 0.0159 | 7.63E+07 | 0.0178 | 0.0244 | 0.4666 |
| rs2294633 | 1 | 20090235 | T | C | -0.0985 | 0.0177 | 2.01E+07 | 0.0188 | 0.0266 | 0.4752 |
| rs2301989 | 7 | 107443871 | A | G | -0.1294 | 0.0161 | 1.07E+08 | 0.0018 | 0.0241 | 0.9429 |
| rs2816980 | 1 | 200078035 | T | G | 0.1941 | 0.026 | 2.00E+08 | -0.0451 | 0.0396 | 0.2861 |
| rs2836881 | 21 | 40466299 | T | G | -0.2217 | 0.0186 | 4.05E+07 | -0.0132 | 0.0288 | 0.643501 |
| rs2838517 | 21 | 45613825 | C | T | -0.1177 | 0.016 | 4.56E+07 | 0.0217 | 0.0244 | 0.3711 |
| rs3024493 | 1 | 206943968 | A | C | 0.21 | 0.0209 | 2.07E+08 | -0.0069 | 0.0336 | 0.8259 |
| rs3097666 | 6 | 33022788 | C | G | -0.2154 | 0.0304 | 3.30E+07 | 0.0463 | 0.05 | 0.36 |
| rs3129299 | 6 | 32900787 | T | C | -0.14 | 0.0236 | 3.29E+07 | -0.0574 | 0.043 | 0.1814 |
| rs3130500 | 6 | 31119976 | A | T | -0.1184 | 0.0183 | 3.11E+07 | -0.0065 | 0.0271 | 0.8128 |
| rs34638686 | 3 | 48682658 | T | C | 0.1795 | 0.0262 | 4.87E+07 | 0.0266 | 0.036 | 0.4515 |
| rs34920465 | 1 | 22700351 | G | A | -0.1708 | 0.0213 | 2.27E+07 | -0.0205 | 0.0409 | 0.5798 |
| rs3812565 | 9 | 139272502 | C | T | 0.1335 | 0.016 | 1.39E+08 | 0.0229 | 0.025 | 0.3632 |
| rs3820328 | 1 | 20142447 | G | A | -0.1662 | 0.0164 | 2.01E+07 | 0.0198 | 0.0247 | 0.4247 |
| rs3823377 | 6 | 29944253 | A | C | 0.1482 | 0.0196 | 2.99E+07 | -0.0225 | 0.0243 | 0.3542 |
| rs41291790 | 6 | 31572664 | A | G | 0.9494 | 0.0806 | 3.16E+07 | -0.0737 | 0.0912 | 0.4133 |
| rs45593732 | 6 | 31583725 | T | C | 0.4903 | 0.0523 | 3.16E+07 | 0.0589 | 0.056 | 0.2848 |
| rs4676408 | 2 | 241574401 | A | G | 0.1433 | 0.0167 | 2.42E+08 | -0.0261 | 0.025 | 0.2976 |
| rs4711173 | 6 | 28514583 | C | T | 0.1166 | 0.0201 | 2.85E+07 | 0.0213 | 0.0277 | 0.4413 |
| rs4728142 | 7 | 128573967 | A | G | 0.0995 | 0.0158 | 1.29E+08 | 0.0032 | 0.0242 | 0.8941 |
| rs4845604 | 1 | 151801680 | A | G | -0.1608 | 0.0239 | 1.52E+08 | -0.0292 | 0.0378 | 0.4455 |
| rs4993442 | 22 | 30253256 | T | G | -0.0988 | 0.0179 | 3.03E+07 | 0.036 | 0.0266 | 0.1706 |
| rs56062135 | 15 | 67455630 | T | C | 0.1078 | 0.0184 | 6.75E+07 | 0.0002 | 0.0274 | 0.9954 |
| rs6017342 | 20 | 43065028 | C | A | 0.1944 | 0.017 | 4.31E+07 | -0.021 | 0.026 | 0.3982 |
| rs6062496 | 20 | 62329099 | A | G | 0.1359 | 0.0163 | 6.23E+07 | -0.044 | 0.0257 | 0.08806 |
| rs62180181 | 2 | 199852010 | T | C | 0.1226 | 0.0171 | 2.00E+08 | 0.0057 | 0.0253 | 0.8239 |
| rs6658353 | 1 | 161469054 | C | G | -0.1569 | 0.016 | 1.61E+08 | -0.0442 | 0.0241 | 0.06613 |
| rs67111717 | 5 | 176790162 | G | A | 0.0944 | 0.0171 | 1.77E+08 | 0.0383 | 0.0257 | 0.1361 |
| rs6889364 | 5 | 40347469 | A | G | 0.1318 | 0.0228 | 4.03E+07 | 0.0316 | 0.031 | 0.3043 |
| rs6933404 | 6 | 137959235 | C | T | 0.1486 | 0.0188 | 1.38E+08 | 0.0321 | 0.0305 | 0.3004 |
| rs7203363 | 16 | 68587692 | A | T | 0.1071 | 0.0189 | 6.86E+07 | -0.012 | 0.0321 | 0.707801 |
| rs72704802 | 5 | 554211 | T | C | -0.1223 | 0.0206 | 5.54E+05 | -0.0374 | 0.0366 | 0.3076 |
| rs7523335 | 1 | 8180210 | A | G | -0.1389 | 0.021 | 8.18E+06 | 0.0108 | 0.0271 | 0.6911 |
| rs7544646 | 1 | 2496649 | G | C | -0.1168 | 0.016 | 2.50E+06 | 0.0124 | 0.0243 | 0.6077 |
| rs755374 | 5 | 158829294 | T | C | 0.1714 | 0.0171 | 1.59E+08 | 0.0017 | 0.0271 | 0.9863 |
| rs7554511 | 1 | 200877562 | A | C | -0.1448 | 0.0178 | 2.01E+08 | 0.0247 | 0.0302 | 0.412 |
| rs7608697 | 2 | 61204641 | C | A | 0.1597 | 0.0161 | 6.12E+07 | 0.0212 | 0.025 | 0.4005 |
| rs77108272 | 6 | 31455675 | A | C | 0.5479 | 0.062 | 3.15E+07 | -0.1003 | 0.0707 | 0.1443 |
| rs78064630 | 19 | 10562802 | A | G | 0.1759 | 0.0308 | 1.06E+07 | 0.038 | 0.0517 | 0.457999 |
| rs79051659 | 1 | 151757843 | A | G | 0.1605 | 0.0264 | 1.52E+08 | 0.0062 | 0.049 | 0.9062 |
| rs7911117 | 10 | 27179596 | G | T | -0.1342 | 0.0239 | 2.72E+07 | -0.0181 | 0.0356 | 0.6045 |
| rs7911680 | 10 | 101293468 | C | A | -0.1525 | 0.0159 | 1.01E+08 | -0.0199 | 0.0242 | 0.4067 |
| rs798506 | 7 | 2788912 | C | T | -0.1206 | 0.0179 | 2.79E+06 | -0.0266 | 0.0255 | 0.2989 |
| rs8073117 | 17 | 70639396 | A | G | -0.1548 | 0.0225 | 7.06E+07 | 0.04 | 0.0362 | 0.2687 |
| rs872956 | 6 | 33076090 | A | T | -0.1378 | 0.021 | 3.31E+07 | -0.0242 | 0.0295 | 0.4145 |
| rs9271176 | 6 | 32578127 | G | A | -0.3495 | 0.0173 | 3.26E+07 | 0.0354 | 0.0244 | 0.1542 |
| rs9611131 | 22 | 39662480 | C | T | -0.1494 | 0.0227 | 3.97E+07 | 0.0505 | 0.0374 | 0.1819 |

UC, Ulcerative colitis; TNF-α, Tumor Necrosis Factor-α; MR, Mendelian randomization

Table S15 Summary information on the SNPs used as genetic instruments for the UC in MR study in celiac disease

| SNP | chr | pos | A1 | A2 | Exposure (UC) | | | Outcome (CeD) | | |
| --- | --- | --- | --- | --- | --- | --- | --- | --- | --- | --- |
|  |  |  |  |  | β | Se | *P* | β | Se | *P* |
| rs10761659 | 64445564 | 10 | G | A | 0.1276 | 0.016 | 1.33E-15 | -0.0207825 | 0.0187661 | 0.2681 |
| rs11209026 | 67705958 | 1 | A | G | -0.483 | 0.0358 | 1.99E-41 | 0.00796817 | 0.0368486 | 0.8288 |
| rs1131095 | 49714225 | 3 | C | T | 0.1593 | 0.0168 | 2.18E-21 | 0.0601539 | 0.0201602 | 0.00284702 |
| rs11651246 | 40759937 | 17 | G | T | 0.147 | 0.0219 | 2.01E-11 | -0.00652122 | 0.0261136 | 0.8028 |
| rs12825700 | 68492980 | 12 | A | G | 0.1889 | 0.0161 | 7.33E-32 | 0.0139029 | 0.018859 | 0.461 |
| rs12936409 | 38043649 | 17 | T | C | 0.1365 | 0.0158 | 5.62E-18 | -0.00702462 | 0.0184371 | 0.7032 |
| rs13200059 | 111943234 | 6 | A | G | 0.2944 | 0.0436 | 1.48E-11 | -0.00823381 | 0.0539506 | 0.8787 |
| rs1359946 | 27536972 | 13 | A | G | 0.1571 | 0.0202 | 6.58E-15 | 0.0246926 | 0.0237311 | 0.2981 |
| rs1736161 | 16833222 | 21 | A | G | -0.1227 | 0.0161 | 2.22E-14 | -0.0425944 | 0.0186738 | 0.0225502 |
| rs2045241 | 114428783 | 11 | A | G | -0.1063 | 0.0169 | 2.83E-10 | -0.0125788 | 0.019585 | 0.520701 |
| rs2836881 | 40466299 | 21 | T | G | -0.2217 | 0.0186 | 1.11E-32 | -0.0163327 | 0.0209306 | 0.4352 |
| rs2838517 | 45613825 | 21 | C | T | -0.1177 | 0.016 | 1.78E-13 | -0.0188218 | 0.0186703 | 0.3134 |
| rs3024493 | 206943968 | 1 | A | C | 0.21 | 0.0209 | 7.46E-24 | -0.0456252 | 0.0256738 | 0.0755492 |
| rs4728142 | 128573967 | 7 | A | G | 0.0995 | 0.0158 | 3.23E-10 | 0.00697561 | 0.0191526 | 0.7157 |
| rs55905347 | 199638507 | 2 | A | G | 0.1054 | 0.0166 | 2.09E-10 | 0.00299551 | 0.0197094 | 0.8792 |
| rs6017342 | 43065028 | 20 | C | A | 0.1944 | 0.017 | 3.95E-30 | -0.0188218 | 0.0190286 | 0.3226 |
| rs6062496 | 62329099 | 20 | A | G | 0.1359 | 0.0163 | 8.97E-17 | 0.00531409 | 0.0187711 | 0.7771 |
| rs6889364 | 40347469 | 5 | A | G | 0.1318 | 0.0228 | 7.87E-09 | 0.0256677 | 0.0276933 | 0.354 |
| rs6933404 | 137959235 | 6 | C | T | 0.1486 | 0.0188 | 2.69E-15 | 0.244514 | 0.0219375 | 7.50E-29 |
| rs72704802 | 554211 | 5 | T | C | -0.1223 | 0.0206 | 2.89E-09 | -0.0101514 | 0.0236762 | 0.668099 |
| rs7523335 | 8180210 | 1 | A | G | -0.1389 | 0.021 | 3.42E-11 | 0.0962189 | 0.0238852 | 5.62E-05 |
| rs755374 | 158829294 | 5 | T | C | 0.1714 | 0.0171 | 9.73E-24 | 0.0129162 | 0.0201975 | 0.5225 |
| rs7554511 | 200877562 | 1 | A | C | -0.1448 | 0.0178 | 4.27E-16 | -0.111602 | 0.0208706 | 8.93E-08 |
| rs78064630 | 10562802 | 19 | A | G | 0.1759 | 0.0308 | 1.08E-08 | -0.0506619 | 0.0362205 | 0.1619 |

UC, Ulcerative colitis; Ced, celiac disease; MR, Mendelian randomization
