# Supplementary material for: The Role of Inflammatory Biomarkers in Mediating the Effect of Inflammatory Bowel Disease on nonmalignant Digestive System Diseases: A Multivariable Mendelian Randomized Study
Source: Can J Gastroenterol Hepatol. 2024 Mar 18;2024:1266139. doi: 10.1155/2024/1266139 (PMC10963109; doi:10.1155/2024/1266139)
Supplement: Supplementary Materials — Additional file 1: Table S1. The differences between the various methods. Table S2. The F-statistic in instrumental variable strength analysis. Table S3. Summary information on the SNPs used as genetic instruments for the CD in MR study in acute pancreatitis. Table S4. Summary information on the SNPs used as genetic instruments for the CD in MR study in irritable bowel syndrome. Table S5. Summary information on the SNPs used as genetic instruments for the CD in MR study in gastroesophageal reflux disease. Table S6. Summary information on the SNPs used as genetic instruments for the CD in MR study in cholelithiasis. Table S7. Summary information on the SNPs used as genetic instruments for the CD in MR study in celiac disease. Table S8. Summary information on the SNPs used as genetic instruments for the UC in MR study in acute pancreatitis. Table S9. Summary information on the SNPs used as genetic instruments for the UC in MR study in irritable bowel syndrome. Table S10. Summary information on the SNPs used as genetic instruments for the UC in MR study in gastroesophageal reflux disease. Table S11. Summary information on the SNPs used as genetic instruments for the UC in MR study in Cholelithiasis. Table S12. Summary information on the SNPs used as genetic instruments for the UC in MR study in Interleukin-6. Table S13. Summary information on the SNPs used as genetic instruments for the UC in MR study in c-reactive protein. Table S14. Summary information on the SNPs used as genetic instruments for the UC in MR study in Tumor necrosis factor-α. Table S15. Summary information on the SNPs used as genetic instruments for the UC in MR study in celiac disease. Additional file 2: Figure S1 Leave-one-out analysis and forest plots for causal effect of Crohn's disease on outcome. Figure S2. Leave-one-out analysis and forest plots for causal effect of ulcerative colitis on outcome. Figure S3. Leave-one-out analysis and forest plots for causal effect of ulcerative colitis on inter [file 1266139.f1.zip › Additional files2 (1).docx]

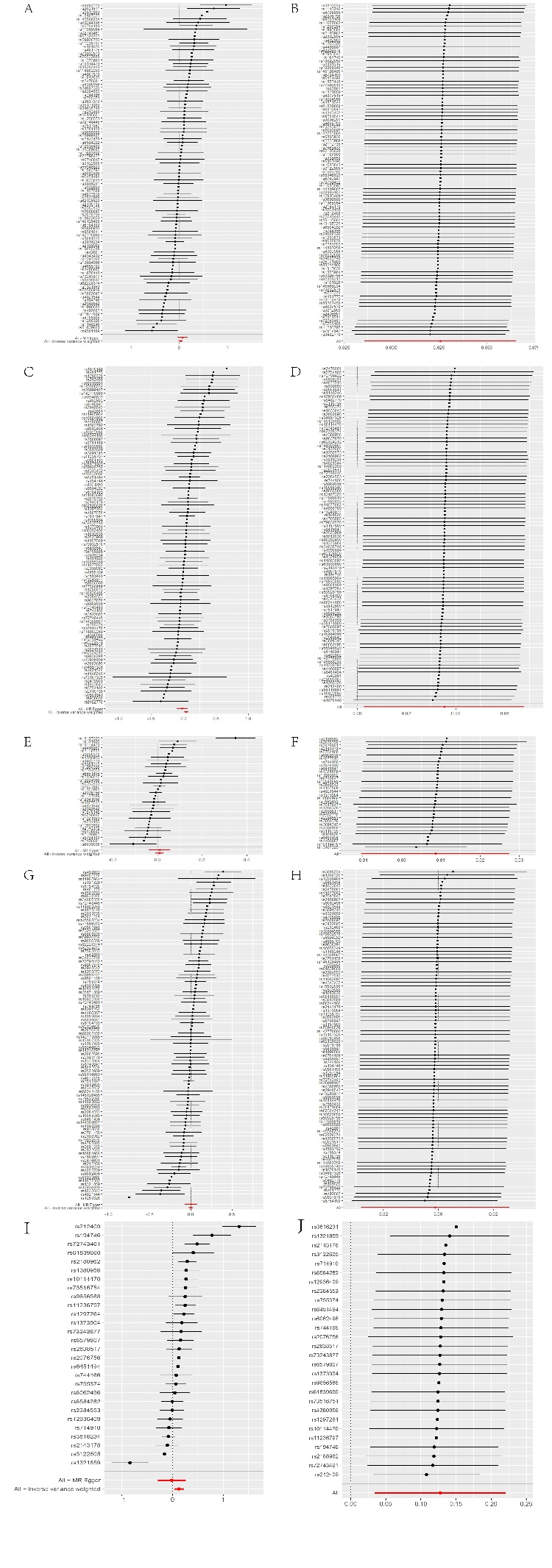


Figure S1. Leave-one-out analysis and forest plots for causal effect of Crohn’s disease on outcome (A) Forest plot: Crohn’s disease and acute pancreatitis; (B) Leave-one-out: Crohn’s disease and acute pancreatitis; (C) Forest plot: Crohn’s disease and irritable bowel syndrome; (D) Leave-one-out: Crohn’s disease and irritable bowel syndrome; (E) Forest plot: Crohn’s disease and gastroesophageal reflux disease; (F) Leave-one-out: Crohn’s disease and gastroesophageal reflux disease; (G) Forest plot: Crohn’s disease and cholelithiasis; (H) Leave-one-out: Crohn’s disease and cholelithiasis; (I) Forest plot: Crohn’s disease and celiac disease; (J) Leave-one-out: Crohn’s disease and celiac disease


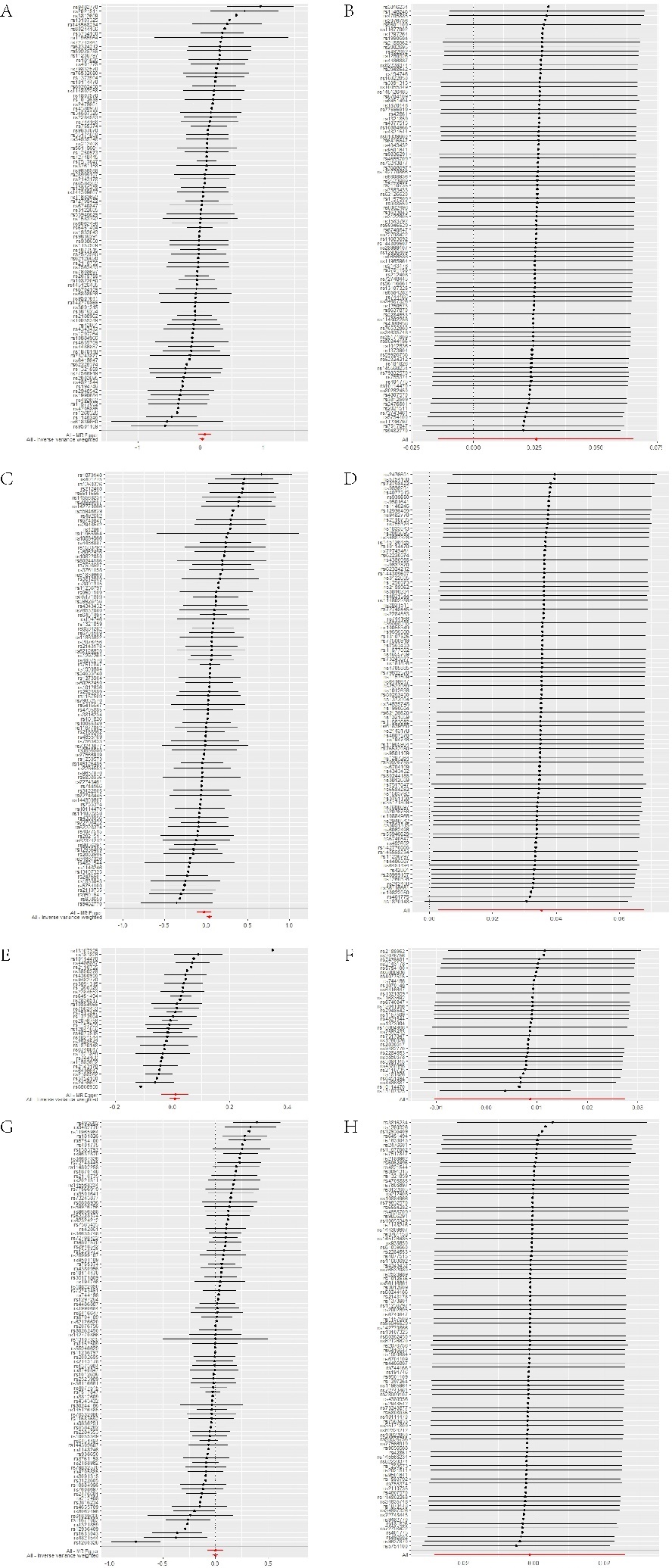


Figure S2 Leave-one-out analysis and forest plots for causal effect of ulcerative colitis on outcome (A) Forest plot: ulcerative colitis and acute pancreatitis; (B) Leave-one-out: ulcerative colitis and acute pancreatitis; (C) Forest plot: ulcerative colitis and irritable bowel syndrome; (D) Leave-one-out: ulcerative colitis and irritable bowel syndrome; (E) Forest plot: ulcerative colitis and gastroesophageal reflux disease; (F) Leave-one-out: ulcerative colitis and gastroesophageal reflux disease; (G) Forest plot: ulcerative colitis and cholelithiasis; (H) Leave-one-out: ulcerative colitis and cholelithiasis (I) Forest plot: ulcerative colitis and celiac disease; (J) Leave-one-out: ulcerative colitis and celiac disease


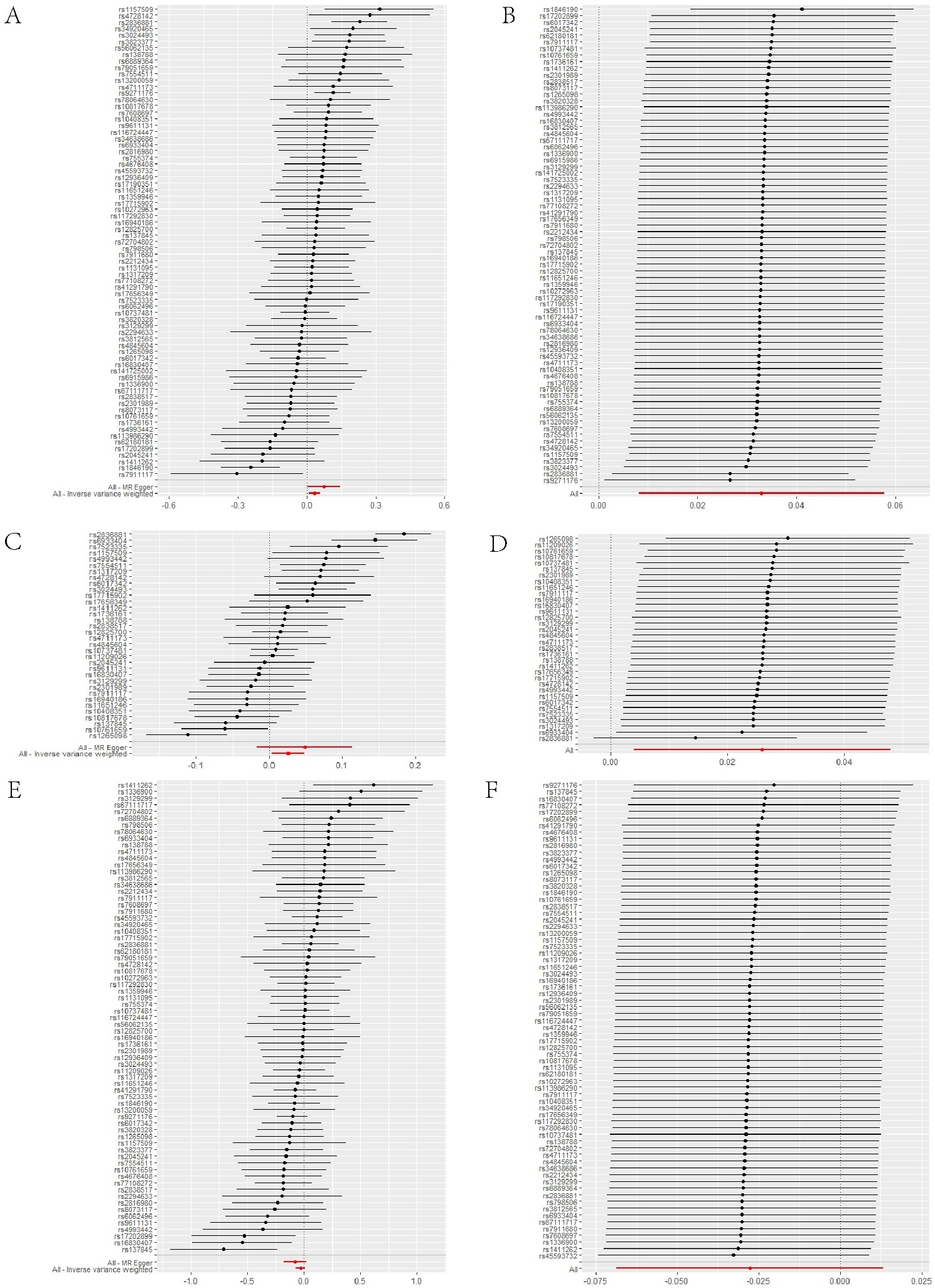


Figure S3 Leave-one-out analysis and forest plots for causal effect of ulcerative colitis on interleukin-6, c-reactive protein and Tumor necrosis factor-α (A) Forest plot: ulcerative colitis and interleukin-6; (B) Leave-one-out: ulcerative colitis and interleukin-6; (C) Forest plot: ulcerative colitis and c-reactive protein; (D) Leave-one-out: ulcerative colitis and c-reactive protein; (E) Forest plot: ulcerative colitis and Tumor necrosis factor-α; (F) Leave-one-out: ulcerative colitis and Tumor necrosis factor-α;


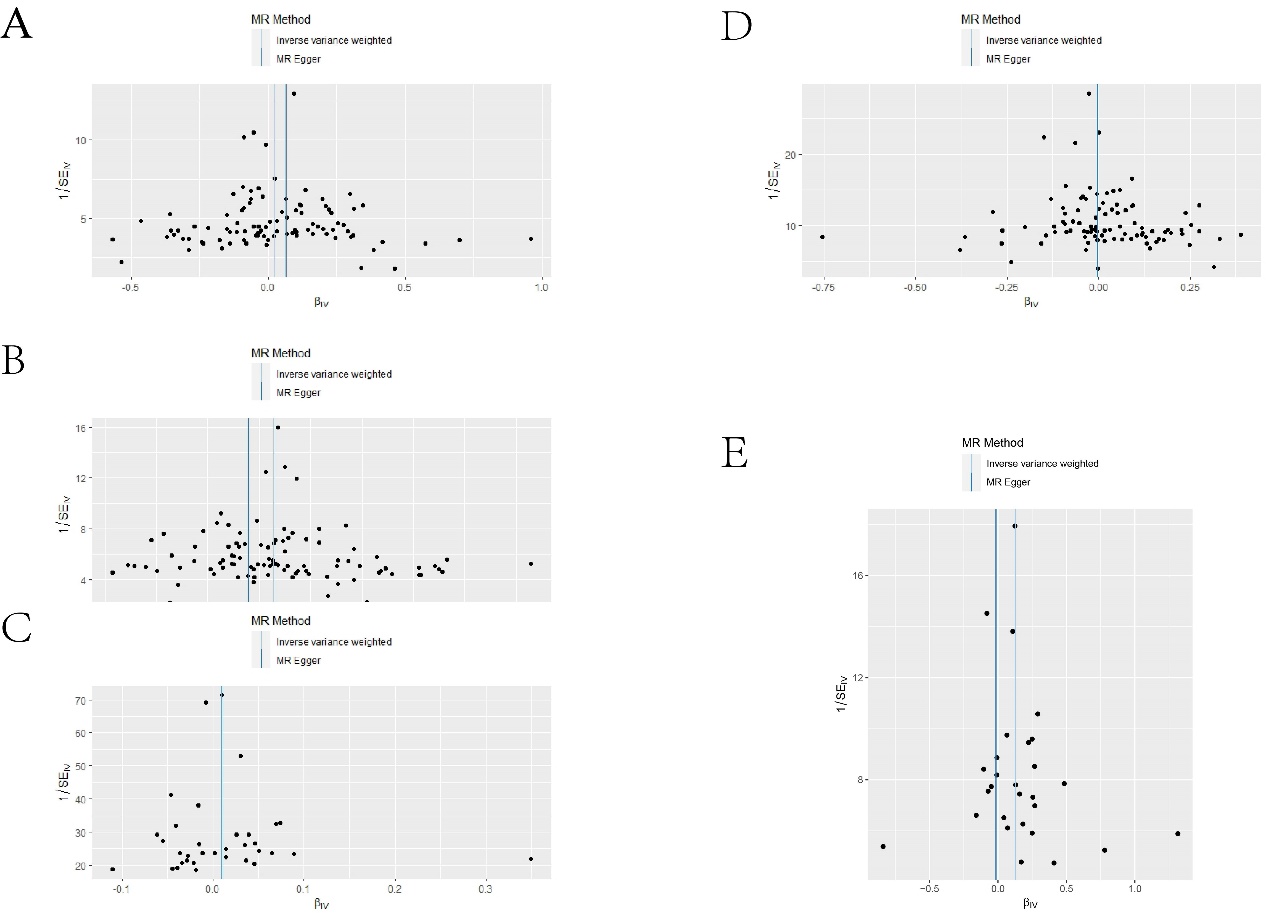


Figure S4. Funnel plot for causal effect of Crohn’s disease on outcome (A) Crohn’s disease and acute pancreatitis; (B) Crohn’s disease and irritable bowel syndrome; (C) Crohn’s disease and gastroesophageal reflux disease; (D) Crohn’s disease and cholelithiasis (E) Crohn’s disease and celiac disease


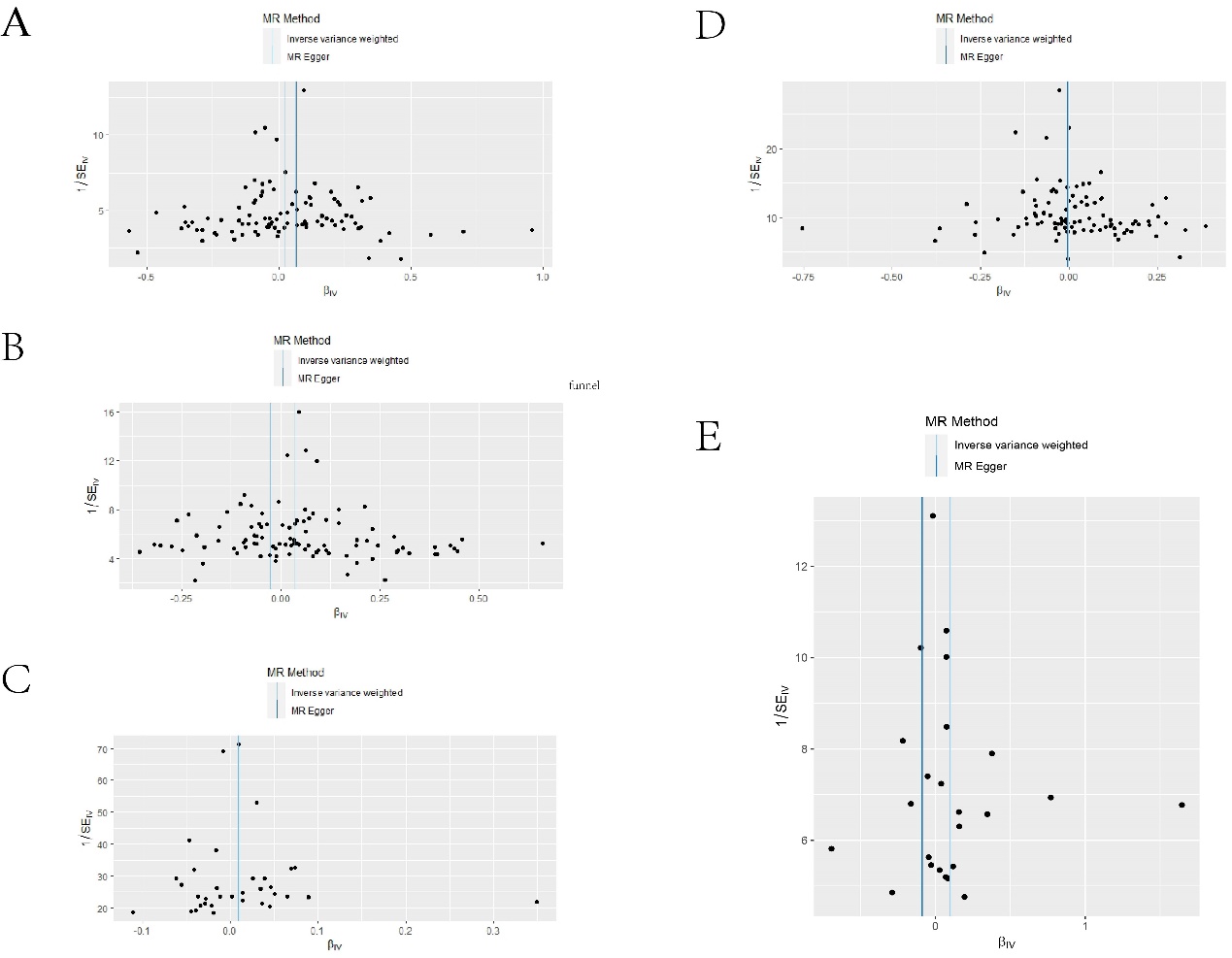


Figure S5 Funnel plot for causal effect of ulcerative colitis on outcome (A) ulcerative colitis and acute pancreatitis; (B) ulcerative colitis and irritable bowel syndrome; (C) ulcerative colitis and gastroesophageal reflux disease; (D) ulcerative colitis and cholelithiasis (E) Crohn’s disease and celiac disease


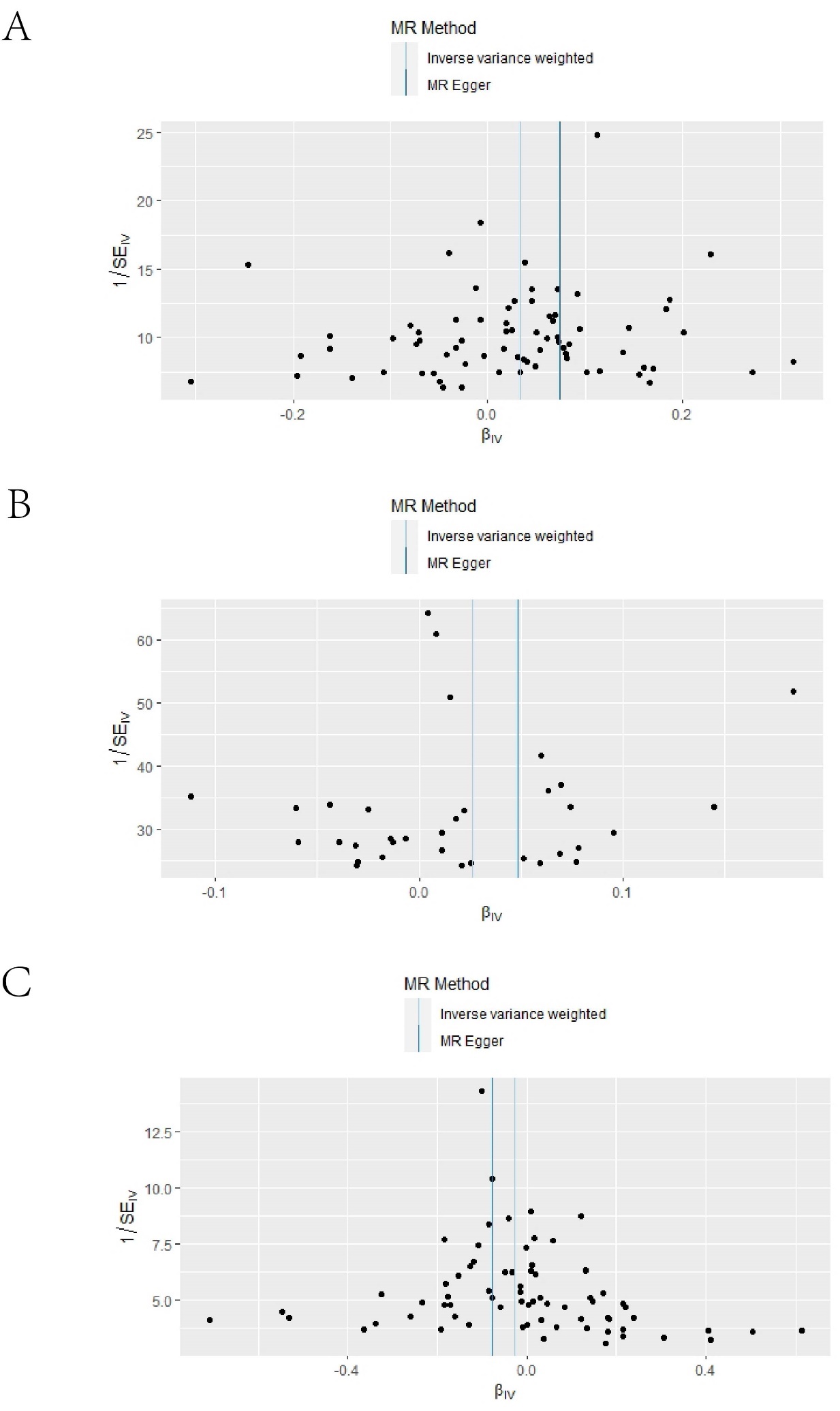


Figure S6 Funnel plot for causal effect of ulcerative colitis on interleukin-6, c-reactive protein and Tumor necrosis factor-α (A) ulcerative colitis and interleukin-6; (B) ulcerative colitis and c-reactive protein; (C) ulcerative colitis and necrosis factor-α
